# Sex-specific chromatin landscapes in an ultra-compact chordate genome

**DOI:** 10.1186/s13072-016-0110-4

**Published:** 2017-01-17

**Authors:** Pavla Navratilova, Gemma Barbara Danks, Abby Long, Stephen Butcher, John Robert Manak, Eric M. Thompson

**Affiliations:** 1 Sars International Centre for Marine Molecular Biology, University of Bergen, 5008 Bergen, Norway; 2 Departments of Biology and Pediatrics and the Roy J. Carver Center for Genomics, 459 Biology Building, University of Iowa, Iowa City, IA 52242 USA; 3 Department of Biology, University of Bergen, 5020 Bergen, Norway

**Keywords:** Histone, Enhancer, Spermatogenesis, Polycomb, Dosage compensation, Heterochromatin, Transposable elements, Endocycle

## Abstract

**Background:**

In multicellular organisms, epigenome dynamics are associated with transitions in the cell cycle, development, germline specification, gametogenesis and inheritance. Evolutionarily, regulatory space has increased in complex metazoans to accommodate these functions. In tunicates, the sister lineage to vertebrates, we examine epigenome adaptations to strong secondary genome compaction, sex chromosome evolution and cell cycle modes.

**Results:**

Across the 70 MB *Oikopleura dioica* genome, we profiled 19 histone modifications, and RNA polymerase II, CTCF and p300 occupancies, to define chromatin states within two homogeneous tissues with distinct cell cycle modes: ovarian endocycling nurse nuclei and mitotically proliferating germ nuclei in testes. Nurse nuclei had active chromatin states similar to other metazoan epigenomes, with large domains of operon-associated transcription, a general lack of heterochromatin, and a possible role of Polycomb PRC2 in dosage compensation. Testis chromatin states reflected transcriptional activity linked to spermatogenesis and epigenetic marks that have been associated with establishment of transgenerational inheritance in other organisms. We also uncovered an unusual chromatin state specific to the Y-chromosome, which combined active and heterochromatic histone modifications on specific transposable elements classes, perhaps involved in regulating their activity.

**Conclusions:**

Compacted regulatory space in this tunicate genome is accompanied by reduced heterochromatin and chromatin state domain widths. Enhancers, promoters and protein-coding genes have conserved epigenomic features, with adaptations to the organization of a proportion of genes in operon units. We further identified features specific to sex chromosomes, cell cycle modes, germline identity and dosage compensation, and unusual combinations of histone PTMs with opposing consensus functions.

**Electronic supplementary material:**

The online version of this article (doi:10.1186/s13072-016-0110-4) contains supplementary material, which is available to authorized users.

## Background

Histone proteins, which package genomic DNA, provide multiple sites for covalent posttranslational modifications (PTMs) by evolutionarily conserved histone modifiers that form multimeric complexes and cooperate with nonhistone proteins [1, 2]. Histone PTMs are associated with chromatin dynamics linked to transcription, replication, DNA repair, recombination, chromosome segregation and other mitotic and meiotic processes [3, 4]. Importantly, in the germ line, they help to secure correct transgenerational inheritance, setting the stage for early embryonic development [5, 6]. Combinatorics of histone PTMs, proposed to constitute a “histone code” [7], are part of the mechanism through which a single genome generates a variety of cell types and states that respond to developmental and environmental cues. Prevalent combinations of modifications, referred to as “chromatin states,” correlate with specific functional regions of the genome, and many appear to be conserved among eukaryotes [8–12]. To date, however, only a few metazoan epigenomes have been studied in detail [10, 13–15], often using cell lines or heterogeneous cell/tissue populations from organisms, with the exception of in vivo cell population studies that focused on a few histone PTMs [16–19]. Here, we present the germline epigenomes of the chordate *Oikopleura dioica*, a member of the lineage that comprises the closest living relatives to vertebrates [20].

*Oikopleura dioica* is a semelparous pelagic tunicate (Urochordate, Appendicularian) with a simple chordate body plan and short, 6-day life cycle [21]. Several major developmental transitions are accompanied by switches between mitotic and endocycling cell cycle modes in both somatic tissues and the ovary [22–24]. *O. dioica* has the smallest metazoan genome sequenced to date, organized in a haploid complement of 3 chromosomes. At 70 Mb, it is ~44-fold smaller than the human genome despite maintaining >18,000 protein-coding genes [25] compared to ~20,000 in humans [26, 27]. Introns are frequently very small (peak at 47 bp; only 2.4% >1 kb), as are intergenic spaces (53% <1 kb). One quarter of the gene complement is organized into operons [28], and *trans*-splicing of a short spliced-leader (SL) RNA occurs at the 5′ ends of 39% of protein-coding genes [29]. Transposable elements (TEs) form a significant proportion of vertebrate genomes, but most vertebrate TE families are absent in *O. dioica* and the density of TEs is low, with most concentrated on the gene-poor Y-chromosome [25]. Major clades of non-LTR (long terminal repeat) retrotransposons are missing from the *O. dioica* genome, but it has variety of LTR retrotransposons from the *Ty3/gypsy* group, divergent from those found in other organisms, as well as *Dictyostelium* intermediate repeat sequence 1 (DIRS1) and Penelope-like elements [30]. These autonomous elements carry an *env* gene and are expressed in a variety of *Oikopleura* tissues including germline-associated cells [31]. A comprehensive developmental transcriptome for *O. dioica* has been assembled and includes ovary and testes samples [32]. The full histone complement and associated PTMs have also been characterized, showing conservation of histone variants and a histone modification repertoire comparable to vertebrates [33].

Regions of the *O. dioica* genome that have potential regulatory function (introns and intergenic regions) have been reduced, often to the order of one nucleosome in size. How this compaction affects long-range enhancer-mediated gene regulation [34] and the epigenetic inheritance of chromatin domains through replication, mitosis and trans-generationally [35] is unknown. Polycomb complexes (PRC 1 and 2), via the tri-methylation of histone 3 on lysine 27 (H3K27me3), govern core mechanisms of metazoan epigenome heritability, organization and developmental dynamics [36]. Polycomb complexes also function in sex chromosome inactivation during dosage compensation [37]. PRC1 is an ancient complex and a determinant of cellular stemness [38], but its canonical composition has been reduced in *O. dioica* and nematodes, possibly correlating with limited cellular plasticity and lack of regeneration [39]. A number of Polycomb complexes and modes of recruitment and function exist, but these vary in the extent to which they have been characterized [40, 41]. *O. dioica* is an interesting model in which to investigate non-canonical Polycomb complexes and their functions in a rapidly evolving lineage.

*Oikopleura dioica* is unusual among tunicates in that it has genetically determined separate sexes with heterogametic (XY) males and homogametic (XX) females. Organisms with heterogametic sex chromosomes have evolved dosage compensation mechanisms to equalize the abundance of transcripts produced by the single X-chromosome in males and the double X-chromosome in females [42, 43]. In male mammals, flies and worms, transcription of genes on the X-chromosome is upregulated. In female mammals one X-chromosome is inactivated, and in hermaphrodite worms, expression from X-chromosomes is downregulated. Different underlying components and molecular mechanisms behind the recruitment and targeting of dosage compensation complexes as well as the resulting changes in chromatin have been well documented [44]. A common feature is that complexes with other functions in the organism, such as Polycomb in mammals, DCC in worms, or fly MSL, have been recruited for domain regulation of X-linked genes. It has thus far not been established that dosage compensation occurs in *O. dioica*, nor through what mechanism it might be achieved, if it does occur.

Here, we sampled *O. dioica* testis, at early day 6 (a few hours before germ-cell release), when it is a syncytium of mitotically proliferating, transcriptionally active, spermatogonia nuclei (Additional file 2: Fig. S1). A proportion of active somatic genes including housekeeping, self-renewal and proliferation genes are required for mitosis and germline reprogramming. At the same time, a testis-specific transcriptional program is required for initiating spermatogenic gene transcription, setting up the transmission of epigenetic memory and poising developmental genes for expression following fertilization. Transitioning between these processes is rapid in the *O. dioica* male germ line, but meiosis itself occurs only in late day 6, about 2 h before spawning. The day 6 *O. dioica* ovary consists of one single giant cell (the coenocyst), where endocycling nurse nuclei share a common cytoplasm with meiotic nuclei arrested in prophase I [24, 45] (Additional file 2: Fig. S1). These two populations of nuclei occur in equivalent numbers, but the ploidy of nurse nuclei (200C) compared to that of the prophase I meiotic nuclei (4C) [22] means that the nurse nuclei dominate (98% contribution) the chromatin content of the ovary. Nurse nuclei are terminally differentiated and help direct oocyte maturation and cellularization. A large portion of their transcriptional output is maternal mRNA that is subsequently stocked in the oocytes. Unlike testis or oocyte meiotic nuclei, nurse nuclei do not traverse mitosis and do not need to re-establish post-mitotic epigenetic landscapes or undergo germline-specific gene repression.

We extracted homogeneous nuclear populations from testes and ovaries and profiled key histone PTMs and nonhistone chromatin-associated proteins to explore chromatin state landscapes in *O. dioica* germ lines and their relationship to genome compaction, sex chromosomes and autosomes. We found RNAPII activity-linked signatures known from other metazoans. Chromatin domains were generally reduced in size, but we did identify regions with histone PTMs typical of enhancers. The ovarian, nurse nuclear epigenome consisted of large domains of active transcription and a general lack of repressive heterochromatin. The male germline epigenome contained chromatin states specific to the spermatogenic program and the X-chromosome and included an intriguing combination of histone PTMs on the Y-chromosome, which may be involved in regulating the activity of transposable elements. This work provides the first comprehensive view of a protochordate epigenome, providing insight into its organization in two sex-specific tissue samples.

## Results

### *Oikopleura* histone PTMs and their combinations

**Fig. 1:**
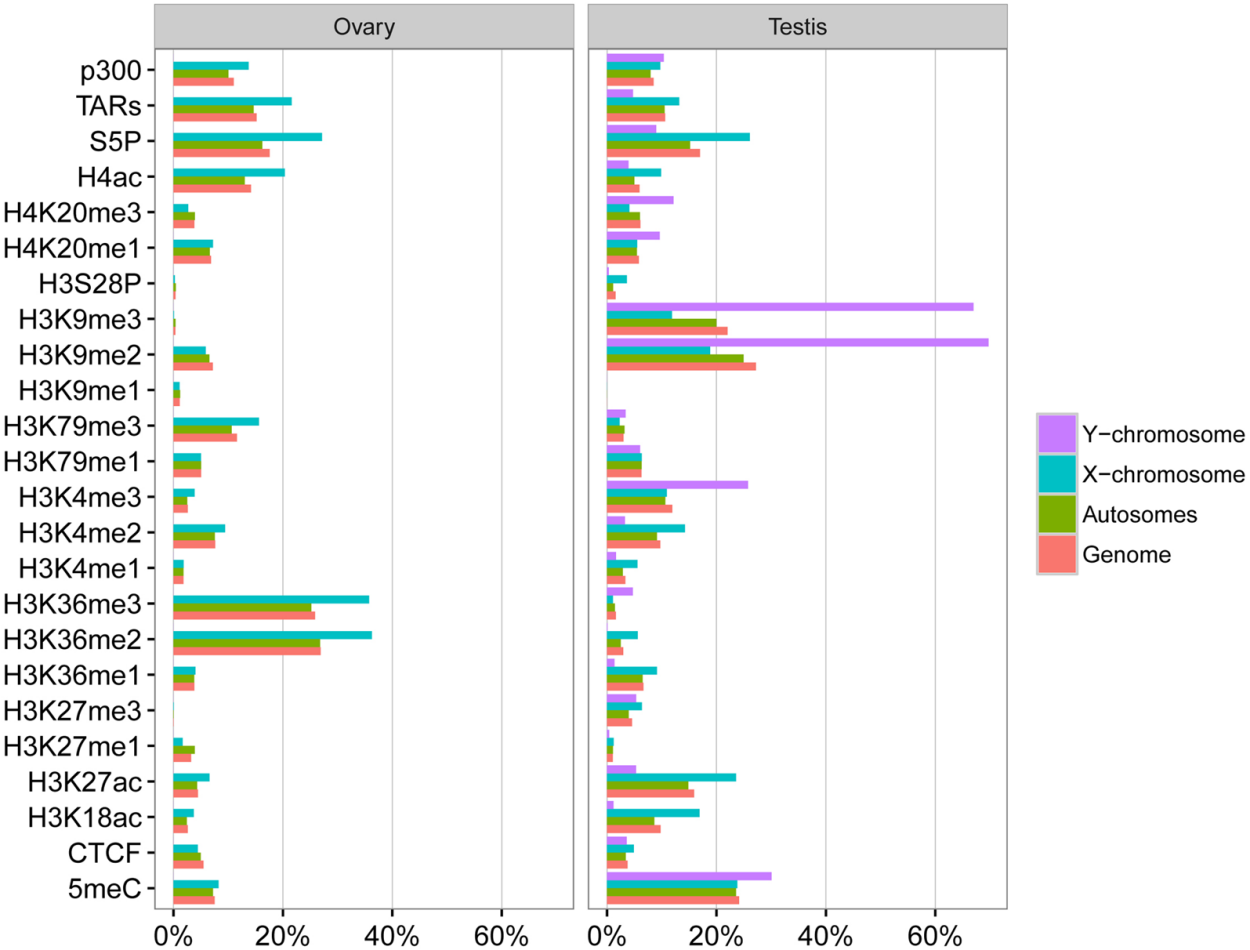
Percentage of genome, autosomes, X- and Y-chromosomes covered by ChIP-enriched regions for each ChIP sample, as well as percentages of the genome covered by transcriptionally active regions (TARs) (using previously published tiling array data from [32], and their definition of a TAR as “any stretch of consecutive positive probes in a particular sample”), in the *O. dioica* ovary and testis

We profiled the following in maturing *O. dioica* testes and ovaries: 19 histone PTMs (Additional file 3: Table S1), using native ChIP-chip; CTCF, p300 and RNA polymerase II (RNAPII) occupancy, using cross-linked ChIP-chip; and 5-methylcytosine DNA methylation (5 mC), using meDIP-chip. Sampled testes were in the mitotically dividing pre-meiotic (spermatogonia) stage, whereas ovaries were dominated by endocycling, transcriptionally active nurse nuclei. We focused on histone H3 and H4 PTMs and related these profiles to gene expression levels [32], *trans*-splicing status [28], chromosomal location, and GC content of promoters (Fig. 1; Additional file 1: Supplemental Results; Additional file 2: Fig. S2). We compared our results to those in human cells, *Saccharomyces cerevisiae, Drosophila melanogaster* and *C. elegans* (Table 1) [8, 10, 12–15, 46–52].

**Table 1:**
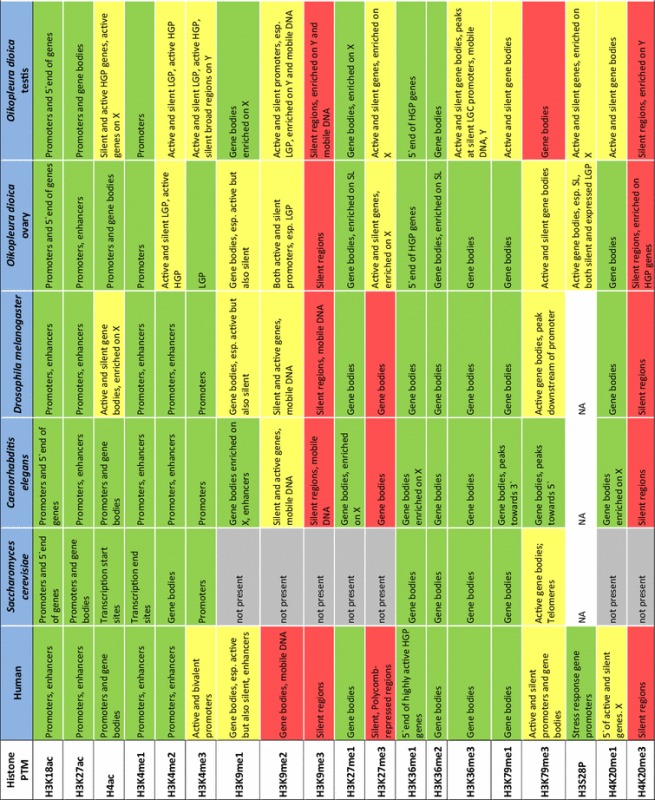
Comparative residency of histone PTMs on genomic features of diverse eukaryotes

Assignments of features marked by histone PTMs for species other than *Oikopleura dioica* were based on reference literature as follows: human [12, 13, 46–49]; *Saccharomyces cerevisiae* [50–52]; *Caenorhabditis elegans* [12, 14, 15]; *Drosophila melanogaster* [8, 10, 12]. Associations of individual histone modification *Oikopleura dioica* were assigned based on assessment of enrichment plots shown in Supplemental Figure S1. These plots show the mean signal intensity around gene start and end sites with genes grouped according to their expression level, the GC content of their promoter, their trans-splicing status and chromosomal locations. We used the error bars indicating 95% confidence intervals on the mean to define (by the lack of their overlap) relative enrichments between compared gene sets as significant. Colors indicate transcriptional activity of features marked by PTMs: green = transcribed; red = repressed; yellow = both active and silent. Esp. = especially; X = X-chromosome; Y = Y-chromosome; HGP/LGP = high/low GC content promoter; SL genes = genes whose transcripts are subject to trans-splicing of the splice leader (SL) sequence; N = not analyzed

**Fig. 2:**
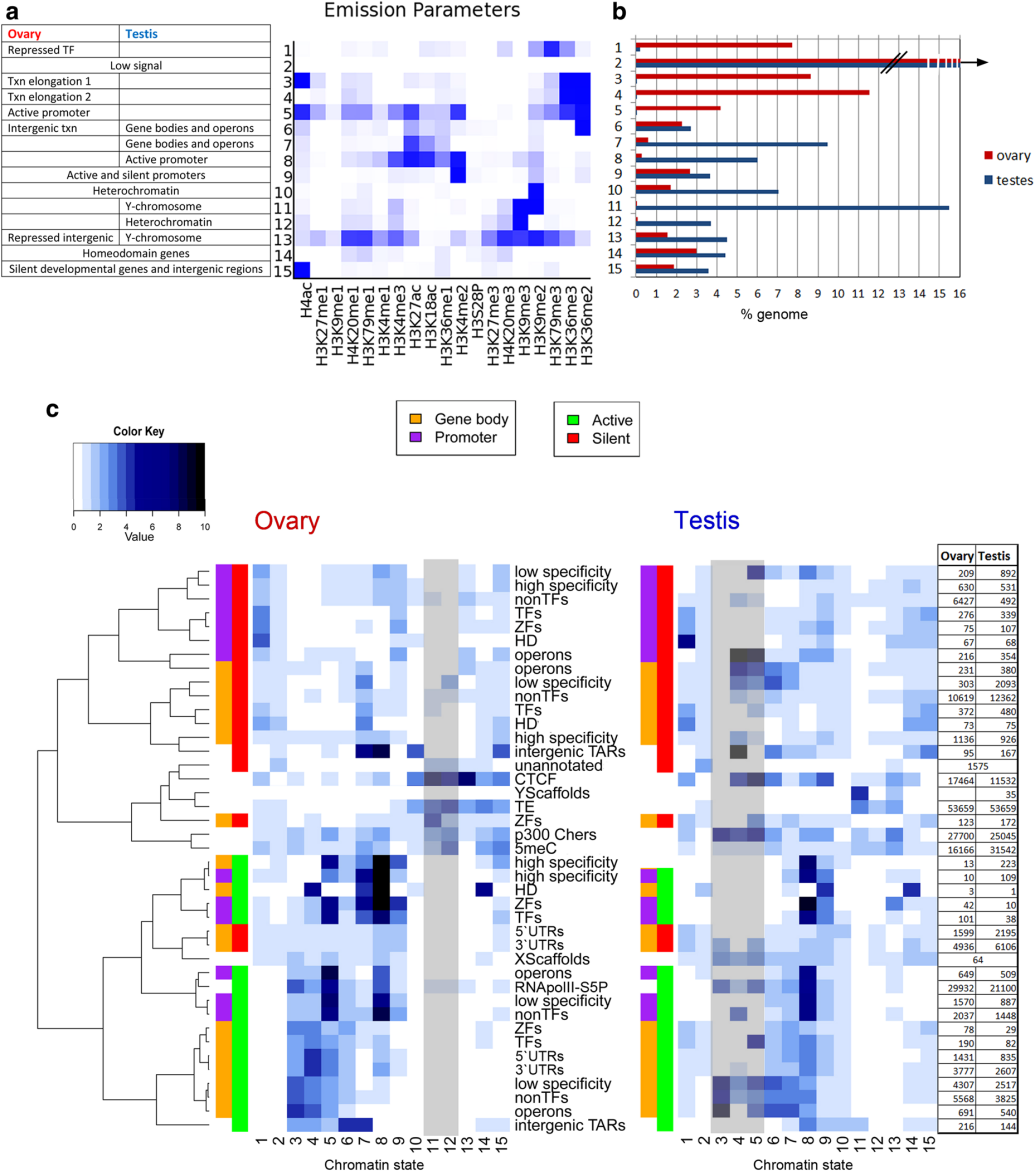
Chromatin states in the *O. dioica* ovary and testis reveal two distinct epigenetic landscapes. Heatmap of model emission parameters (**a**) for 15 chromatin states learned across the genome, using both samples (see also Additional file 3: Table S3). Proportions of the genome in the ovary and testis covered by each chromatin state (**b**) show sex/tissue specificity of chromatin states. Heatmaps (**c**) visualize the fold enrichments of each chromatin state (*columns*) for a set of genomic features (*rows*), as listed, in the ovary and testis. This facilitates assignment of putative biological function(s) of each chromatin state in each tissue. *Gray shading* indicates states that cover below 0.1% of the genome (70,000 bp) in each tissue. These low-coverage states nevertheless have large fold enrichments over certain genomic features, e.g., state 3 has low coverage in the testis but is found more often than expected by chance at active operons. Features are clustered according to their correlation in the ovary, and this ordering is used for the testis heatmap to allow comparison. The table gives the numbers of each feature in each sample. Gene bodies (*orange*) and promoter regions (*purple*) are split into active (*green*) and silent (*red*) states in the respective ovary and testis tissues. Txn, transcription; TF, transcription factor; ZF, zinc finger protein; HD, homeodomain protein, high- and low-specificity genes according to breadth of expression across development (see “Methods” section); TAR, transcriptionally active region; TE, transposable element; unannotated, regions lacking annotation or transcription; 5meC, methylcytosine; RNApolII-S5P, serine 5 phosphorylated RNA polymerase II

Combinatorial deposition of chromatin marks was analyzed by classifying testis and ovary chromatin into 15 states (Fig. 2a; Additional file 3: Tables S2 and S3), learnt jointly across both cell types, using a multivariate hidden Markov model (chromHMM) [53]. These 15 states were reproducible when processing ovary and testes datasets independently (Additional file 2: Fig. S3). Functions were assigned to jointly learned states according to their enrichments in an array of transcriptionally repressed and active genomic features (Fig. 2c). Feature annotation of the separately learnt 15-state models underscored some different uses of individual modifications in the testis versus ovary (Additional file 2: Figs. S3 and S4). We were also able to resolve 50 biologically meaningful chromatin sub-states (Additional file 1: Supplemental Results; Additional file 2: Fig. S5; and Additional file 3: Table S4) including a Polycomb-repressed state (state 46: enriched for H3K27me3 and marks promoters of silent developmental genes) that was less pronounced in the 15-state models. Unless stated otherwise, all subsequent analyses were based on the main functional chromatin states captured by the jointly learnt 15-state model.

**Fig. 3:**
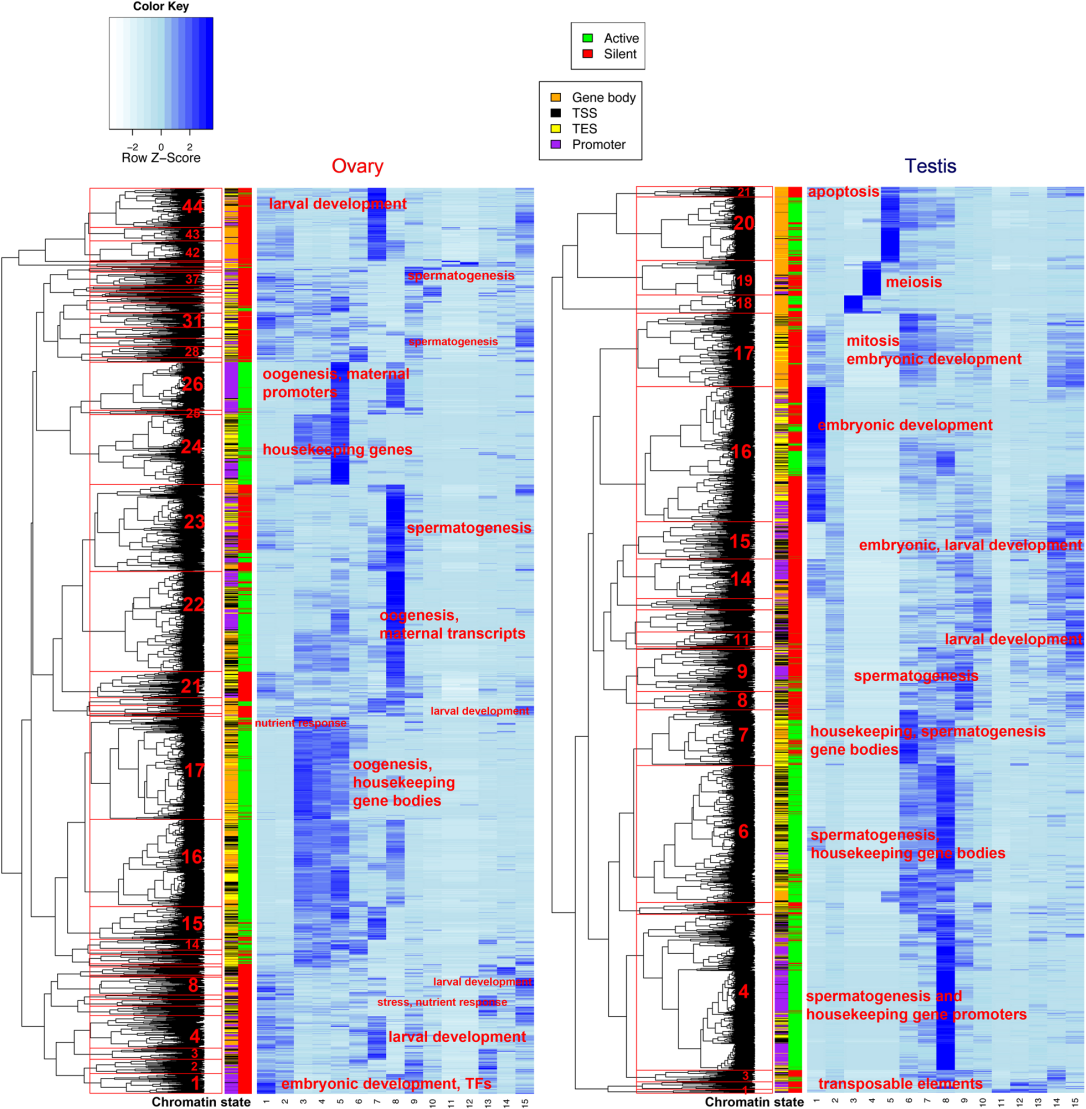
Gene ontology chromatin state enrichments (*light to dark blue* = low to high enrichment) in promoters (*purple*), gene bodies (*orange*), TSSs (*yellow*) and TESs (*black*) of active (*green*) and silent (*red*) genes grouped by GO term and clustered. Selected clusters are labeled with representative functions and numbered, to facilitate reference in “Results” section and in Additional file 3: Table S5

Four chromatin states were specific to the ovary (1, 3, 4, 5), and four were specific to the testis (7, 8, 11, 12). Specific chromatin states were associated with active promoters (states 5, 8 and 9), transcription elongation (states 3, 4, 6, 7) and silent regions (states 1, 10–12, 14–15), which included states specific to the Y-chromosome (state 11) and silent transcription factors (TFs) (state 1). State 2, which had no enrichment of any profiled modifications, covered 54% of the ovary and 40% of the testis genomes (Fig. 2b), similar to the sum of “weak signal” states calculated for human (45%), fly (35%) and worm (45%) [12]. We grouped active and silent genes by GO terms and calculated chromatin state enrichments on their promoters and gene bodies to reveal differential use of chromatin states on genes with different biological functions (Fig. 3; Additional file 3: Table S5).

**Fig. 4:**
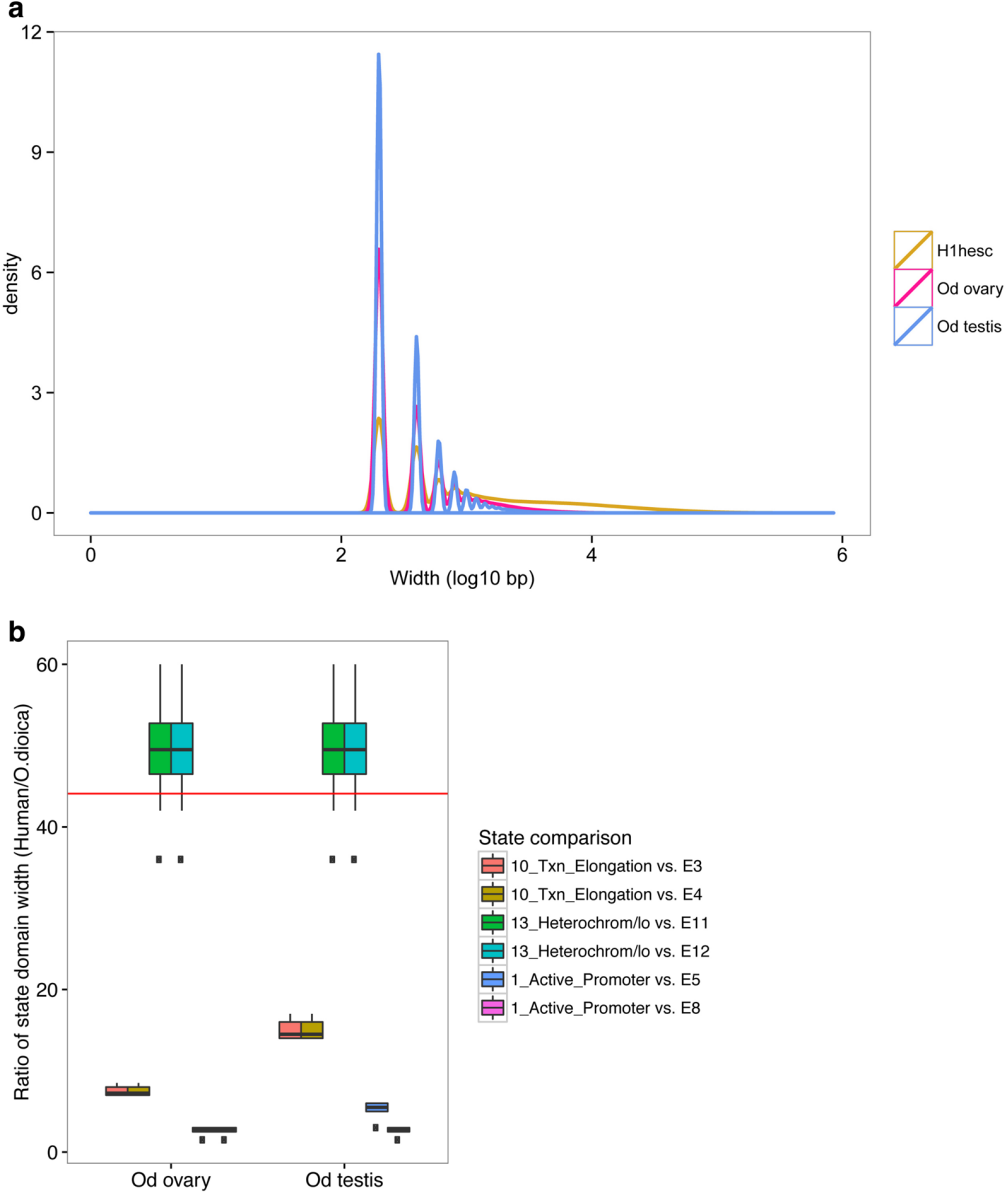
Compact chromatin state domains in the *O. dioica* epigenome. **a** Distributions of all chromatin state domain widths for each cell type in *O. dioica* compared to those in human embryonic stem cells (H1-hESC) [92]. *O. dioica* domain widths were adjusted for the difference in resolution (50 vs. 200 bp) by rounding *O. dioica* widths up to the nearest 200 bp. **b** Comparison of the ratios of human state domain widths for active promoters, transcriptional elongation and heterochromatin (see Additional file 2: Fig. S6) to corresponding state domains in *O. dioica*. *Each box* summarizes the ratios of median domain widths (*y*-axis) for the human cell lines relative to the similar domains in each *O. dioica* tissue (*x*-axis). The *red line* indicates the ratio of the human genome size to that of *O. dioica* (44-fold smaller). Relative to the genome compaction, heterochromatin domains in *O. dioica* are disproportionately more compact

We compared chromatin state domain widths in *O. dioica* to those found in nine human cell lines [9] and found that both activating and repressive domains were significantly narrower in *O. dioica* (Mann–Whitney test: *W* = 1.189401e+12, *p* value <2.2e−16; Additional file 2: Fig. S6). Domains spanning over 7 nucleosomes were largely absent (Fig. 4a). The absence of large repressive regions in *O. dioica* was notable (Fig. 4b; Additional file 2: Fig. S6) and supports previous observations of a decline in heterochromatin coverage with decreasing genome size [12].

**Fig. 5:**
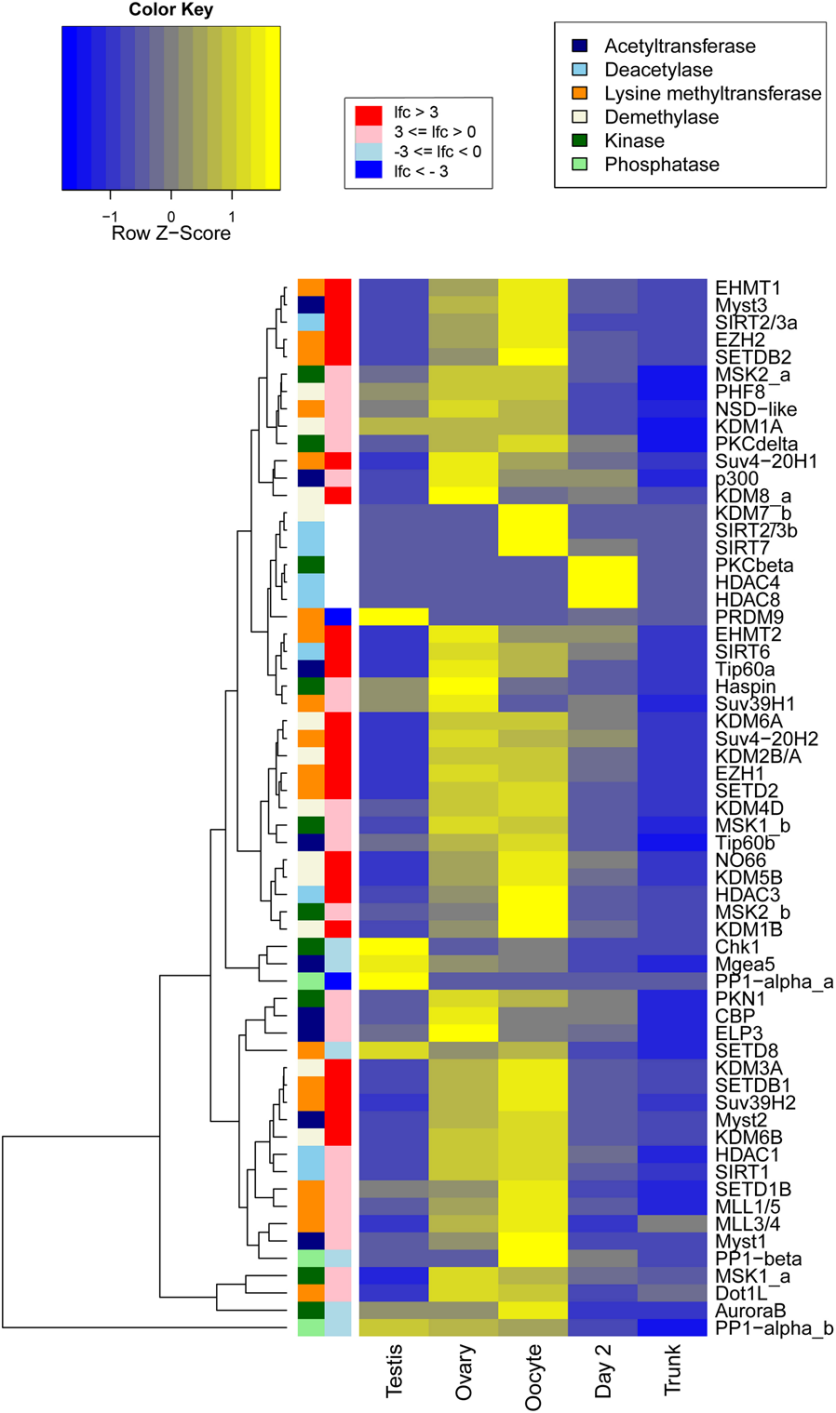
Expression profiles of *O. dioica* chromatin modifier genes showing the main histone methyltransferases, demethylases, acetyltransferases, deacetylases, kinases and phosphatases (indicated by *colored bars*) at relevant stages of development, where *blue* is low and *yellow* is high expression. Log_2_ fold expression difference between ovary and testis is indicated by a *graded bar* where *darkest blue* is testis specific and *darkest red* is ovary specific (see also Additional file 3: Table S6)

We found homologs of human histone modifier proteins and extracted gene expression values for these homologs from a previously published transcriptomic dataset [32]. The expression patterns of the complement of *O. dioica* histone modifiers in testes and ovaries corresponded to the presence or absence of their associated histone PTMs. Gene duplications of some modifiers and losses of others, particularly those related to DNA repair, hormone response, Hox gene activating, RA response and histone methyltransferases, reflected *O. dioica’s* reduced NHEJ DNA repair toolkit, altered Polycomb complex complement and dispersion of developmental gene clusters (Fig. 5; Additional file 3: Table S6; examples and details in Additional file 1).

### Transcriptionally permissive chromatin in the ovary

The ovarian chromatin landscape was dominated by ovary-specific states 3–5 (Fig. 2a, b). These states were enriched for H3K36me2 and H3K36me3 (typical of transcribed gene bodies in metazoans [46]), covered 25% of the genome (Fig. 2b), overlapped RNAPII-occupied regions and correlated with high levels of transcription. Active genes in the ovary were mostly related to housekeeping functions, maternal transcription and oogenesis (Fig. 3; Additional file 3: Table S5). *Trans*-spliced genes, despite no significant differences in mean expression levels compared to non-*trans*-spliced genes, had higher enrichment for these two methylation marks (Additional file 2: Fig. S2A). States 3–5 were also more enriched on operons (a subset of *trans*-spliced genes) (Fig. 2c). State 3 was distinct from state 4 in its higher enrichment of H4ac, higher prevalence in UTRs compared to gene bodies and higher enrichment in regions of RNAPII occupancy and transcribed operons. Interestingly, state 3 was also enriched at the TSS in a subset of silent genes annotated with GO terms related to nutrient response (cluster 17, Fig. 3). This may reflect RNAPII pausing in genes that regulate oocyte production in a nutrient-dependent manner.

Promoters of operon genes were enriched for promoter state 5, characterized by H4ac, H3K27ac, H3K4me2/3 and H3K36 methylations. The typically active gene body marks (H3K36me and H4ac) in these promoters may reflect the uncertainty of TSS annotations for a subset of *trans*-spliced operon transcripts (since a stretch of 5′ sequence is removed from mRNAs and replaced by the SL RNA). Enrichment of H3K4me3, the hallmark of active promoters [54], was overrepresented at active promoters of operons compared to silent operon promoters (Fisher’s test *p* value = 2.044 × 10^−10^) but not at regions surrounding the 5′ ends of expressed downstream operon genes. This provides evidence that these are indeed co-transcribed from a single upstream promoter. Active promoter state 8 (H3K27ac, H3K18ac, H3K4me2/3) was enriched in active genes related to oogenesis and embryonic development (presumably maternal transcripts stocked in oocytes: cluster 22, Fig. 3), but also marked a group of silent spermatogenesis genes in the ovary (cluster 23, Fig. 3).

### RNAPII pausing

Pausing of RNAPII is a pervasive feature of promoters in metazoans that facilitates integration of multiple cellular signals, poising genes for rapid expression and/or synchronous activation [55]. In *O. dioica*, 15% of the ovary genome and 8% of the testis genome are transcribed [32]. Serine-5 phosphorylated RNAPII ChIP-chip, however, showed near-equal occupancy across the genome: 17.6% of the genome in the ovary and 17.0% in the testis were enriched for RNAPII S5P (Fig. 1), suggesting that paused RNAPII is frequent in *O. dioica*. We identified 1106 genes in the ovary and 1389 genes in the testis, which have proximal promoter regions that are repressed but associated with RNAPII (16 and 18% of silent genes in the ovary and testis, respectively, which is higher than expected in the testis *χ* ^2^ = 12.17, *df* 1, *p* value = 4.85 × 10^−4^). Most of these genes encode proteins for RNA-processing functions, cell cycle and to a lesser extent, development, in both samples, and for nutrient response functions, particularly in the ovary (Additional file 2: Fig. S7). Such a mechanism is consistent with the observation that *O. dioica* can rapidly adjust its gamete output in response to shifting nutrient conditions (e.g., algal blooms) [28, 56, 57].

### Enhancers in the compact *Oikopleura* genome

We identified 8070 regions of H3K4me1 enrichment that did not overlap annotated gene start sites in the ovary and 12,272 in the testis. Of these, 1087 and 6829 overlapped regions of H3K27ac enrichment in the ovary and testis, respectively, and constituted candidate enhancers. We intersected these with regions associated with the co-activator p300, a tissue-specific enhancer-binding histone acetyltransferase [58, 59], and found 353 and 2281 strong candidates of active (p300-bound) enhancers in the ovary and testis, respectively. Sites bound by p300 frequently co-localized with the E2F transcription factor and CTCF (Additional file 2: Fig. S8). Chromatin states enriched at p300-binding sites (Fig. 2c) included states 5 and 8, which contained typical enhancer PTMs. Enhancer marks were present to a lesser extent in state 13, which comprises a complex combination of histone PTMs, and heterochromatic state 12, which may represent repressed regulatory elements. Highly conserved elements (HCNEs), indicative of regions with conserved regulatory function [60], were identified around genes encoding developmental TFs [25]. HCNEs were enriched in states 1 and 15 in the testis and 1, 6 and 7 in the ovary; states also associated with developmental TFs (Fig. 2c; Additional file 2: Fig. S9). As expected, given that developmental TFs are primarily active during embryogenesis, we did not find any overlap of HCNEs with our candidate enhancers in the gonads. Together the data indicate that enhancers are present in the *O. dioica* genome, despite its high compaction, reshuffling and loss of some regions of evolutionarily conserved synteny. More enhancers would likely be detected in early embryonic and larval samples when developmental regulatory networks are activated.

### Marking of repressed developmental regulatory genes differs in ovary and testis

Repressed TF and zinc finger (ZF) genes in the ovary were associated with chromatin state 1 (enriched for H3K79me3, together with H3K36me3, H3K9me2 and H4K20me3) (Fig. 2a, b; Additional file 3: Table S3). State 1, and H3K79me3 in general, was found almost exclusively in the ovary (Figs. 1, 2a; Additional file 2: Fig. S3) and was enriched in the long introns of silent genes (Additional file 2: Fig. S10). It is interesting, therefore, to note that 3 testis-specific H3t variants have substantial modification of the Dot1 recognition site for H3K79 methylation [33]. Repressed TF, ZF and homeodomain (HD) genes, particularly in the testis, had an unusual signature: strong enrichment for H4ac that resulted in assignment to state #15 (Figs. 2a, c, 3; Additional file 3: Tables S3 and S4). Extended analysis to the 50-state model confirmed these findings and revealed additional marks such as H4K20me1 in addition to H4ac (state 14; Additional file 2: Fig. S5A, C), H3K4me1, H3K27me3 or H3K36me1 (states 43–46). These loci correspond to typical Polycomb targets known from metazoan somatic cells [12]. Interestingly, a group of silent TFs, HD and ZF genes with functions in larval development had gene bodies enriched for state 7 in the ovary (Fig. 3, clusters, 3, 4, 42, 43, 44) and both states 6 and 7 in the testis (Fig. 3, cluster 17). These data indicate that *O. dioica* testes deploy distinct epigenomic states (and possibly histone variants) in the control of important developmental regulatory loci.

### Tissue-specific spermatogenesis-related chromatin states and a switch from SETD2- to NSD-mediated H3K36 methylation in the testis

State 8 was the most prevalent active promoter state in the testis, driving spermatogenesis-related genes (Fig. 3, testis clusters 4 and 6). This profile, to a lesser extent, corresponded to a general signature of tissue-specific gene promoters for the ovary as well. Transcriptional elongation on less tissue-specific expressed genes and operon genes in testis mapped to chromatin states 6 and 7 (Figs. 2a, c, 3b), which consisted of H3K27 and H3K18 acetylations but differed in the presence (state 6) or absence (state 7) of H3K36me2 (Fig. 2a). Remarkably, 9.5% of the testis genome was transcriptionally elongated in the absence or with low enrichment of H3K36me2/3 (Fig. 2a, b). Expression data showed that the SETD2 ortholog (the main histone methyltransferase generating H3K36me3 from H3K36me2) was repressed in the testis and RT-PCR revealed alternative splicing of the SET domain of this gene (not shown). The presence of H3K36me2, independent of gene activity, in state 6 (Fig. 2a) suggests a developmental switch where the SETD2 co-transcriptional deposition of H3K36me2/3 is replaced by an NSD-like H3K36-methyltransferase that is highly transcribed in the testis (Fig. 5; Additional file 3: Table S6). This resembles the switch from somatic met1 (worm SETD2 ortholog) to the germline mes-4 (worm NSD1 ortholog) in *C. elegans*. This switch is essential for production and maintenance of H3K36 methylation patterns during transcription in the parental germ line as well as for proper post-embryonic development of germ cells in the offspring [61].

### A modified chordate Polycomb system

Homologs of core components of the canonical Polycomb complexes were not identified (RNF1, SUZ12, PCGF, SCMH1), and RA signaling is absent *O. dioica* [39, 62]. Several trithorax group proteins (MLL1, Ash1 and PRC-recruiting KDM2B) that activate Hox genes in a cell-specific manner in response to RA [41] were also not found in the *O. dioica* genome (Additional file 3: Table S6). We found, however, duplications for EZH2, which is responsible for PRC2 methyltransferase activity, and the EED subunit, which recognizes H3K27me3 and stimulates an activity-based feedback loop. Both EZH2 paralogs were abundantly transcribed in the endocycling ovary, but only weakly expressed or silent in the testis (Fig. 5; Additional file 3: Table S6). Taken together, these observations raise interesting questions on the mechanism of Polycomb-mediated repression in this chordate lineage.

Prior to H3K27me3 deposition, PRC2 can be recruited in a number of ways that depend on composition of the complex, its interacting partners, target genes and the cell cycle and developmental stage [41, 63, 64]. In nematodes, H3K27me3 can remain on daughter chromatin through replication and serve as a template for PRC2-mediated deposition [65]. G9a- [66] or EZH2-mediated H3K27me1 deposition has also been reported as a step toward H3K27me3 [67]. Mitotic H3S28P creates an optimal substrate for Polycomb deposition [68, 69], but can also displace the complex and induce a methyl-acetylation switch of the adjacent K27 residue, depending on the cell cycle stage [70]. In *Drosophila* [71] and mammals [72], PRC exhibits a preference for CG-rich sequences, but we did not observe H3K27me3 enrichment on such sequences in testes or ovaries (Additional file 2: Fig. S2B).

We observed broad regions of H3K27me3 in the *O. dioica* genome at a lower enrichment level than other marks (Additional file 2: Figs. S11 and S12) and therefore did not resolve distinct Polycomb repressive states in the 15-state model. The 50-state model, however, resolved a typical Polycomb state 46, unambiguously marking promoters of silent TF, ZF and HD genes (Additional file 2: Fig. S5; Additional file 3: Table S5). In the testis, H3K27me3 regions co-localized with H3K27me1 and H3K4me1, but not with H3S28P (Additional file 2: Fig. S8). In contrast, in the ovary, H3K27me3 co-localized with H3S28P and H3K4me1 but not H3K27me1, the latter mark instead being strongly associated with transcriptional elongation and H3K36me2/3 (Additional file 2: Figs. S8 and S11). We also observed that H3K27me3 blocks were largely complementary to those of H3K36me2 in *O. dioica* (Additional file 2: Fig. S11A). It is known that mes-4 (NSD1)-dependent H3K36 methylation not only marks germ-cell-expressed genes in the germ line of adult nematodes, but also leads to exclusion of PRC2 (mes2/3/6) from germline-activated genes and repression of genes normally expressed in somatic cells, as well as genes on the X-chromosome [61, 73]. This mechanism would explain X-chromosome bias of H3K27me3 and complementarity of the two marks in the *O. dioica* testis although besides NSD1, H3K36me3 could in addition be deposited by the activity of male-specific PRDM9 [74]. Testis X-chromosome genes are less transcriptionally active than those in the ovary (cf. TARs in Fig. 1) despite similar RNAPII occupancy.

### PRC2 as a central component of dosage compensation in *Oikopleura*

The *O. dioica* X-chromosome is gene rich and was transcriptionally active in both day 6 heterogametic (XY) males and homogametic (XX) females. Autosomal and X-chromosome genes have similar expression levels in both ovary and testis (mean X:A ratio: 1.1 in the ovary, 1.12 for constitutively expressed genes; and 0.88 in the testis, 0.99 for constitutively expressed genes; see also Additional file 2: Fig. S13), indicating dosage compensation. Several histone PTMs showed differential enrichment on autosomes versus the X-chromosome (Additional file 2: Fig. S2C). Ovarian X-chromosomes showed depleted levels of H3K27me1, the first step in Polycomb repression and memory, but were enriched in H3K27me3, the final Polycomb-repressed state. We interpret this as a possible dosage compensation mechanism.

In the testis, transcriptionally active promoters on the X-chromosome had a higher enrichment of H4ac, H3K27me1 and H3K4me1 compared to transcriptionally active genes on autosomes. Repressive H4K20me3, H3K9me2 and 3, strongly enriched on the Y-chromosome, were depleted from the X-chromosome as compared to autosomes (Additional file 2: Fig. S2C, D). On the other hand, the active promoter-associated H3K9me1 [12, 13] was enriched on male X-chromosome genes, similar to observations in *C. elegans* [14]. Enrichment of H3S28P in the testis was higher around X-chromosome genes compared to autosomes (Additional file 2: Fig. S2C). This may be related to Polycomb-mediated higher-order chromatin structures and/or higher residence/recruitment of PRC on this chromosome during mitotic divisions.

Together, our data revealed specific distributions of marks on sex chromosomes and are consistent with the Polycomb system being a central component of the *O. dioica* dosage compensation system.

### H3K4me3 and H3K36me3 enriched heterochromatic states linked to mobile DNA elements

We resolved several distinct repressive chromatin states (11–13). While we found regions of mutual exclusion of the repressive marks H3K27me3 and H3K9me3 in *O. dioica* (Additional file 2: Fig. S12), we also found that they overlap in heterochromatin (Fig. 2a, state 13 in the 15-state model and Additional file 2: Fig. S5; states 18, 26, 29 in the 50-state model, Additional file 2: Fig. S8). This overlap was previously thought to be specific to worms [12], although cross talk between PRC and G9a/GLP in regulating a subset of genes has been found [66] and these H3K27 and H3K9-, mono- and di-methyltransferases are known to form multimeric complexes with tri-methyltransferases in human cells [2].

The *O. dioica* Y-chromosome was enriched for repressive states characterized by H3K9me3, H3K9me2 and H4K20me3 (Fig. 2a, states 11 and 13; Additional file 2: Fig. S12). In the testis, mobile DNA elements were also enriched for these states. State 11 (H3K9me2/3 heterochromatic marks) was enriched at regions of 5mC despite limited DNA methylation potential in *O. dioica* (DNMT1 and 3 absence) (Fig. 2a, c).

Beside the conserved role of H3K4me3 at active promoters in *O. dioica* (Fig. 2c, chromatin states 5 and 8), we also found broad regions of this mark within heterochromatin states 11, 12 and 13 (and states 16, 19 and 18 in the 50-state model, Additional file 2: Fig. S5). Independent support for this observation comes from immunolocalizations on both *O. dioica* diploid and endocycling nuclei, showing H3K4me3 signals in RNAPII-depleted heterochromatic knobs together with other typical heterochromatic marks and DNA methylation [75]. Intriguingly, we also found that regions containing the active transcription mark, H3K36me3, coincided with these heterochromatic regions in the testis (Fig. 2c; Additional file 2: Fig. S5, state 18 in the 50-state model; Fig. S12).

**Fig. 6:**
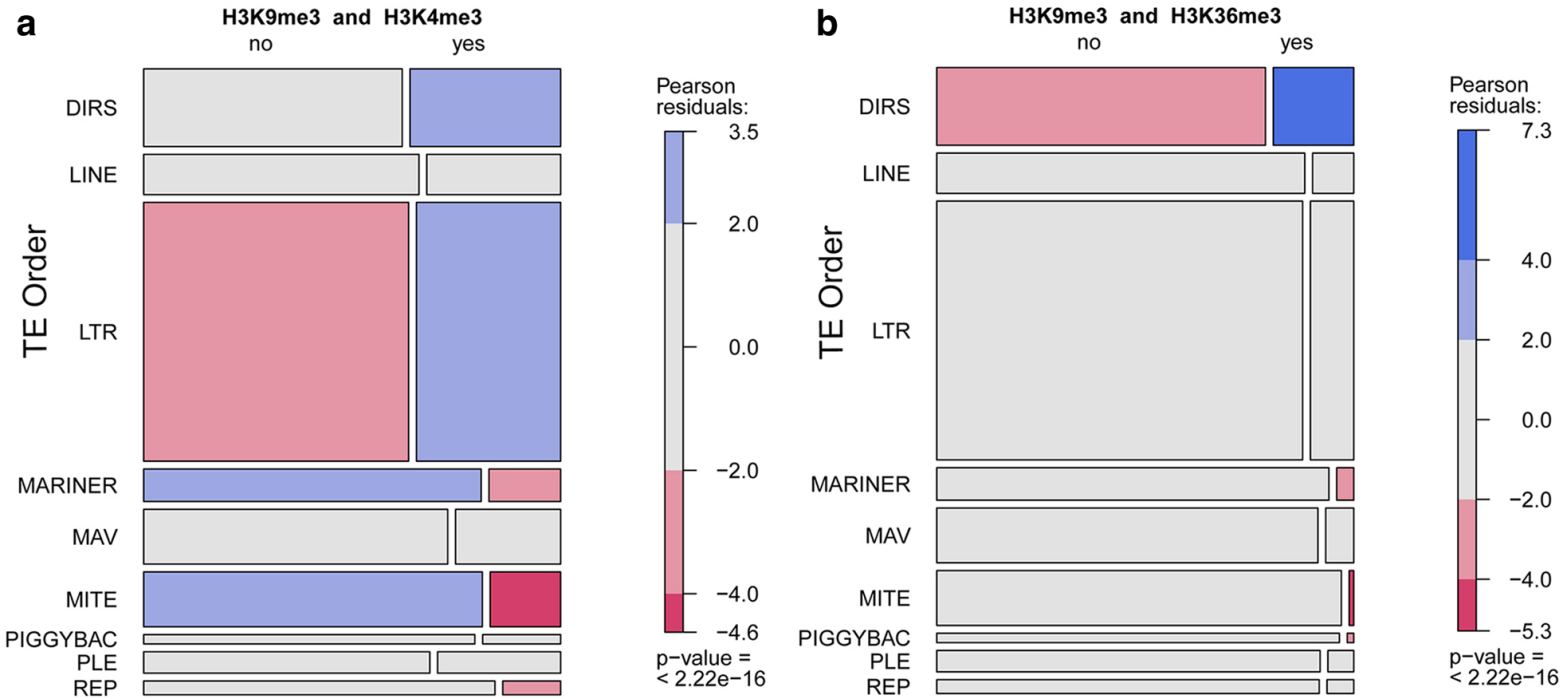
Mosaic plots of Pearson Chi-squared tests for the association of the type of transposable element (TE) on the Y-chromosome and its “bi-modal” overlap with regions of enrichment of heterochromatic H3K9me3 and one of the active marks H3K4me3 (**a**) or H3K36me3 (**b**). *DIRS, Dictyostelium* intermediate repeat sequence; LINE, long interspersed nuclear element; LTR, long terminal repeats; MAV, maverick; MITE, miniature inverted-repeat transposable elements; PLE, PiggyBac-like element; REP, repetitive extragenic palindromic

We found a significant relationship between the type of TE on the Y-chromosome and whether or not it overlapped the heterochromatic mark H3K9me3 and either of the active marks H3K4me3 or H3K36me3 (*χ* ^2^ = 97.454, *df* 8, *p* value <2.2e−16). The TE orders DIRS (102 regions out of 519) and LTR (183 out of 1729) both had an unusually high frequency of this combination of heterochromatin and active marks, whereas Mariner, MITEs and repeats annotated as possible Env had lower than expected frequencies. We tested for the combination of H3K9me3 with each active mark separately and obtained similar results when considering H3K9me3 with H3K4me3 (*χ* ^2^ = 95.685, *df* 8, *p* value <2.2e−16) (Fig. 6a). When considering only H3K9me3 and H3K36me3, however, the DIRS order was the only one that had a higher than expected frequency of the two marks together (X-squared = 109.76, *df* 8, *p* value <2.2e−16) (Fig. 6b). These retrotransposons carrying tyrosine recombinases are widespread in eukaryotes. Their sequences are bordered by terminal repeats related to their replication via free circular dsDNA intermediates that are integrated without duplications in the site of integration [76].

## Discussion

Compared to vertebrates, the protochordate *Oikopleura dioica* has undergone strong secondary genome compaction and morphological simplification, characteristic of the rapidly evolving larvacean lineage. *O. dioica* is the only known larvacean with separate sexes, and this is rare in tunicates in general [77] indicating an ancestral hermaphroditic state from which separate sexes and heteromorphic sex chromosomes have evolved more recently in only a few species. In this study, we compared the chromatin landscapes of the *O. dioica* ovary and testis and assessed differences between the autosomes and sex chromosomes. A greater diversity of chromatin states was deployed in the testes, compared to the ovary, paralleling the previously observed amplification of testes-specific histone variants [33].

Chromatin states in *O. dioica* included the typical epigenetic signature of active promoters conserved among other metazoans (H3K4me3, H3K27ac, H3K18ac, weak H3K4me1 and H3K79me2 [12]). Tissue-specific promoter states also often marked gene bodies. This has also been observed in other species on frequently transcribed genes [78] and may be a consequence of very short coding regions and introns and/or high RNAPII processivity in *O. dioica*.

Histone acetylations are generally associated with accessible chromatin and are related to transcriptional activation. We found, however, silent developmental genes marked by H4ac, particularly in the testis (state 15). In endocycling nurse nuclei, silent developmental genes were enriched in H3K79me3 (state 1) (e.g., sox2 locus; Additional file 2: Fig. S12) though these two marks also co-occurred in both sexes to some extent, as resolved by the 50-state model (states 43–45, Additional file 2: Fig. S5). All three methylation states of H3K79 are mediated by Dot1 [79]. This mark acts to regulate endocycle progression, and its peak at G1/S prohibits re-replication in mammalian cells [3]. H4 acetylation and H4K20me1 peak during M and early G1 and decrease during S-phase [80] to ensure replication origin licensing and a chromatin state accessible to replication factors [81]. Thus, one potential reason for differential H4ac (testes) and H3K79me3 (ovaries) marking could be related to the different mitotic versus endocycling cell cycle modes. Alternatively, these nucleosomes may be subject to histone variant exchange in the testis. *O. dioica* sperm chromatin does not undergo histone-to-protamine replacement that is typically preceded by massive H4 hyperacetylation in both human and *Drosophila* [82]. Instead, histone H3.3 is replaced by three isoforms of the *O. dioica* testis-specific variant H3t [33], and nucleosomes are retained. Amino acid substitutions surrounding K79 within the H3ts most likely preclude Dot1 binding and its ability to methylate these histones. H4 is also replaced by the male-specific H4t that has two residue changes adjacent to K20 that may hinder the methylation of this residue. Together with the retention of histones in *O. dioica* sperm, the association of generally activating H4ac with silent developmental genes in the testes could be viewed as a potentiating transgenerational mark. The lack of a transition to protamines, and extremely rapid embryonic and larval development, may have increased weighting on the intergenerational transmission of potentiated epigenetic states. Resolving individual lysine acetylations on H4 would be a step toward better understanding this unusual marking.

Along with promoter structure, enhancers determine cell-type specificity of gene expression and are important in developmental switching of metazoan genes. *O. dioica* intergenic and intronic spaces are very limited compared to vertebrate genomes, and the presence of enhancers is as yet poorly defined. We identified a number of candidate enhancer regions in this compact genome, in both the ovary and the testis, using intersections of typical enhancer PTMs and p300-bound regions. Histone PTMs marking enhancers in human, fly, cnidarians and worm (H3K4me1 and 2, H3K27ac, H3K79me2 and 3 [12, 16, 83]) were found in actively differentiating embryonic cells and tissue cell cultures. The *O. dioica* ovary and testis comprise terminally differentiated, highly specialized cell types, and the lower abundance of typical enhancer marks is in accordance with recent models of gene regulation during differentiation [84]. Moreover, genes organized in operons, particularly those devoted to maternal transcripts in the ovary, might have high transcriptional rates by default and be more subject to translational regulation via mTOR signaling [57]. The longer introns of *O. dioica* developmental genes exhibit more conserved intron positioning and also contain HCNEs [25]. We observed enrichment of chromatin states 1 and 15 on HCNEs. State 15 was under-represented in regions bound by p300 in the testis, but in the ovary, p300 co-localized with actively transcribed gene promoter state 5 or with 5mC in silent intergenic regions. The detection of typical active developmental enhancer activity would be further addressed through analysis of embryonic stages.

We found evidence of Polycomb interactions on autosomes and X-chromosomes in both sexes and propose roles of PRC2 in dosage compensation. We also observed in the *O. dioica* male germ line that the somatic H3K36 methyltransferase SETD2 is repressed, leaving NSD-(mes-4)-like and PRDM9-like to deposit H3K36-methylation. Complementary patterns of H3K36me and H3K27me3 in testis indicate regulation of transcript levels in the germline analogous to mes-4 (NSD-like) catalyzed H3K36me in *C. elegans*, which antagonizes PRC on germline genes such that PRC is excluded from autosomes but remains on (and represses) the X-chromosome. The mutual antagonism between PRC2 and mes-4 (NSD-like) is thought to be important in the transgenerational inheritance of germline-specific transcriptional programs in *C. elegans* [61, 85–87]. Thus, similar mechanisms may be operating in *O. dioica*, and it would be of future interest to determine the terminal chromatin states of mature sperm in this regard. In *C. elegans*, this proposed mechanism operates in the absence of DNA methylation or histone replacement by protamines during spermatogenesis. Both of these latter mechanisms are features of mammalian spermatogenic programs, though protamine replacement is not complete and some histones remain. Interestingly, *O. dioica* is intermediate in that DNA methylation is present whereas histone replacement by protamines is absent [33], offering perhaps a useful comparative reference perspective in the evolution of mechanisms assuring transgenerational inheritance of germline transcriptional programs.

Given the rarity of separate sexes among tunicates, *O. dioica* offers an interesting model of more recent independent evolution of heteromorphic sex chromosomes. The *O. dioica* Y-chromosome contains a pseudoautosomal region, accumulated mobile elements, and a few male-specific genes, indicating that Y-chromosomal degeneration progressed rapidly [25]. Evolutionary forces driving Y-chromosomal sequence decay are well studied [88], but little is known about autosomal epigenome transitions toward the largely heterochromatic nature of ancient Y-chromosomes. We observed strong co-localization of heterochromatic marks and 5mC on this chromosome. This cooperation is an ancient feature based on HP1-mediated DNA methyltransferase recruitment [89, 90]. Patterns of histone PTMs on the Y-chromosome reflect the functional state and evolutionary history of the sequences [88]. The combination of histone and DNA modifications on the *O. dioica* Y-chromosome appears to have adapted to repress the activity of accumulated mobile DNA elements [25].

Our finding of unusual chromatin states containing typically heterochromatic marks (H3K9me2, H3K9me3, H4K20me3) combined with active transcription-related ones (H3K4me3, H3K36me3, H3K27ac, H4K20me1, H3K79me1) might represent a transition state in the time course of heteromorphic chromosome evolution. To our knowledge, such patterns of H3K4me3 and H3K36me3 overlapping heterochromatin signatures have been observed only in mammalian imprinted loci [91] and *C. elegans* mobile elements [14]. In the analysis of the epigenomes in nine human cell types, a group of endogenous retroviruses was enriched in a complex chromatin state that consisted of a number of histone modifications including H3K36me3, H3K4me3 and H3K9me3 [92]. However, these states have not been reported specifically on the Y-chromosome. *O. dioica* has little heterochromatin, consistent with a scarcity of TE elements [30] and generally reduced noncoding regions. Most TEs are present on the largely non-recombining Y-chromosome. The genome contains active Tor-family retrotransposons [30] that are transcribed in primordial germ-cell-adjacent somatic cells during embryonic development and in the adult testis [31]. These elements are specifically activated, often from their own non-LTR internal promoters and not genome-wide de-repressed. The presence of H3K4me3 and H3K36me3 on TEs could be a consequence of TE transcription with the co-occurrence of H3K9me3 due to the location of some copies of these elements in repressive chromatin environments, whereas others are active, either within one animal, or in different individuals. Notably, these unusual states were restricted to marking certain classes of TEs in *O. dioica*.

The short life cycle and ability to rapidly regulate gamete output over 3 orders of magnitude [45, 56, 93] may also be relevant to the extent of paused RNAPII signatures we observed in the gonads. Such a strategy would be compatible with more rapid transcriptional response to nutrient availability in adjusting gamete number. Finally, it is suspected that the width of broad chromatin domains contributes to the heritability of epigenetic states because of random segregation of nucleosomes to daughter cells during genome replication [35]. Thus, the probability of loss of an epigenetic state will increase as a function of decreasing breadth of chromatin domains. This poses a challenge to epigenetic regulation and inheritance on the compact *O. dioica genome*, where intergenic regulatory regions are frequently on the order of one nucleosome. Indeed, we found that chromatin state domain widths in *O. dioica* were generally smaller than those in their chordate relatives, the vertebrates, and rarely exceeded 7 nucleosomes in size. Nonetheless, this is evidently compatible with fidelity of heritable transmission of epigenetic states in this species.

## Conclusions

Our work provides the first comprehensive view of a protochordate epigenome. In the transcriptionally active endocycling, ovarian, nurse nuclei, histone modifications linked to RNAPII activity were conserved compared to those in other metazoans. We found evidence that RNAPII pausing is frequently involved in controlling gene transcription during oogenesis. We also identified candidate enhancers that have enhancer PTMs typical of other metazoan enhancer regions. Large heterochromatic domains of H3K9me3 were largely absent in the ovary.

The epigenome of the male germ line reflected a shift from tissue-specific gene expression toward establishment of transgenerational inheritance and showed features related to the mitotic proliferation ongoing in this tissue. Our data support the involvement of a modified Polycomb complex in sex chromosome dosage compensation in homogametic females. We also identified a novel chromatin states that combines both euchromatic and heterochromatic histone PTMs that are associated with specific classes of TEs and the Y-chromosome.

Strong secondary genome compaction and disruption of evolutionarily conserved linear genome architecture in *O. dioica* have been epigenetically accompanied by reduced chromatin state domain widths and a general reduction of heterochromatin. It will be of interest to build on the foundation of the current study to determine how the three-dimensional genomic architecture of topologically associated domains (TADs) has been affected by these processes.

## Methods

### Animal culture and collection

*Oikopleura dioica* were cultured at 15 °C [21]. At late day 5/early day 6, males and females were separated from each other, and gonads were dissected in cold N-ChIP collection buffer (0.32 M sucrose, 1 mM CaCl_2_, 4 mM MgCl_2_, 50 mM Tris/HCl pH 7.7, 0.5 mM PMSF and 1× protease inhibitor. Sampled testes were in the mitotically dividing pre-meiotic (spermatogonia) stage, using H3S28P immunostaining as a reference mark of meiotic versus mitotic events. For histone acetylation samples, 5 mM sodium butyrate was also added, and for histone phosphorylation samples, phosphatase inhibitor (phosphatase inhibitor cocktails 2 and 3, Sigma) was included. Samples were sedimented at 8000 rpm and snap-frozen in liquid nitrogen.

### Native and cross-linked ChIP-chip

We performed native ChIP [94], with minor modifications, using 10 male or 40 female gonads in 300-μl buffer per ChIP. Mammalian and *O. dioica* histone H3 and H4 sequences are identical [33], and we used antibodies (10 μg per ChIP) validated as ChIP grade for human cells (Additional file 3: Table S1). Antibodies were bound to 50 μl Dynabeads^®^ Protein G (Invitrogen) and incubated with chromatin overnight (O/N). Immunoprecipitated DNA was purified by phenol–chloroform–isoamylalcohol extraction and EtOH precipitation followed by Min Elute PCR purification (Qiagen). Resulting DNA was amplified using Whole Genome Amplification kit 2 or 4 (Sigma) according to manufacturer’s instructions (the chemical fragmentation step was omitted). With the exception of H3K4me2 and H3K9me2, histone PTM profiles were replicated at least twice in both ovary and testis samples. Amplified material was labeled and hybridized to *O. dioica* genomic microarrays (Nimblegen) [32].

Animals were fixed in 1% formaldehyde in PBS for 12 min. Cross-linking was stopped by adding glycine to 0.13 M for 5 min followed by 3 washes with cold PBS. Fixed animals were kept in PBS containing 0.5 mM PMSF, 1× protease inhibitor and phosphatase inhibitor for dissection of gonads. Gonads were lysed in RIPA buffer (150 mM NaCl, 1% NP-40, 0.5% Na deoxycholate, 0.1% SDS, 50 mM Tris pH 8, 5 mM EDTA), supplemented with fresh PMSF, protease and phosphatase inhibitor for 15 min. Chromatin was fragmented to a size range of 200–600 bp using a Bioruptor UCD-200 (Diagenode) for 8 × 30 s on/1 min off. ChIP was performed using 300 μl of centrifuged chromatin. We used custom antibodies (twentieth-century biochemicals) against *O. dioica* p300 and CTCF. Antibodies were bound to 50 μl Dynabeads^®^ Protein G and incubated with chromatin O/N. Immune complexes were washed twice with 800 μl RIPA, twice with 500 mM NaCl/RIPA and twice with LiCl buffer (500 mM LiCl, 10 mM Tris–HCl pH 8, 1% sodium deoxycholate, 1% NP-40) and once with TE. Enriched DNA was recovered by 15-min elution at 65 °C in 1% SDS, 50 mM Tris–HCl pH 8, 1 mM EDTA and subjected to cross-link reversal for 5 h at 65 °C followed by RNase A and proteinase K treatments. DNA was then purified as for native ChIP. Immunoprecipitated DNA was amplified using the Whole Genome Amplification 4 kit (WGA4 Sigma), labeled and hybridized to *Oikopleura* genomic microarrays (Nimblegen).

### Methylated DNA immunoprecipitation on chip (MeDIP-chip)

*Oikopleura dioica* genomic DNA from day 6 immature ovaries and testes was isolated using proteinase K lysis followed by RNAse A treatment and phenol–chloroform extraction. Ethanol-precipitated DNA was diluted in 450 μl TE, randomly fragmented by sonication using the Bioruptor UCD-200 (Diagenode). Fragments ranged in size from 200 to 600 bp and were denatured at 95 °C for 5 min. Protein G Dynabeads (50 μl) were coupled to the monoclonal antibody against 5-methylcytidine (5mC) (Eurogentec #BI-MECY-1000). After three washes with IP buffer (10 mM Na-phosphate pH 7.0, 140 mM NaCl, 0.05% Triton X-100), DNA fragments together with 51 μl of 10 × IP buffer were added to the beads and incubated 16 h at 4 °C with constant rotation. Beads were collected with a magnetic rack and washed with 700 μl 1× IP buffer for 10 min with rotation. Washing was repeated twice, followed by elution in proteinase K buffer (50 mM Tris pH 8.0, 10 mM EDTA, 0.5% SDS, 50 μg proteinase K (NEB)) and incubation for 2 h at 55° C with shaking. Immunoprecipitated DNA was purified by phenol–chloroform–isoamylalcohol extraction and EtOH precipitation followed by Min Elute PCR purification (Qiagen). Resulting DNA was amplified using Whole Genome Amplification kit 4 (Sigma) according to manufacturer’s instructions (the chemical fragmentation step was omitted).

### Preprocessing ChIP-chip data

ChIP-chip data were preprocessed using the Bioconductor R package *Ringo* [95]. We normalized raw probe intensities from each sample (Cy5 channel) to corresponding input DNA probe intensities (Cy3 channel) by computing log_2_(Cy5/Cy3). We applied the NimbleGen normalization method, which adjusts for systematic dye and labeling biases by subtracting from individual log_2_ ratios, the Tukey’s biweight mean, computed across each sample’s log_2_ ratios. To reduce noise, we smoothened the normalized log_2_ ratios using a running median across a 150-bp window (approximate nucleosome size) with a minimum threshold of three nonzero probes. We used the resulting log_2_ ratios in all further analyses and browser visualizations.

### Histone PTM distributions and defining ChIP-enriched regions

We defined transcription start sites (TSSs) and transcription end sites (TESs) using gene models on the *O. dioica* reference genome [25]. We categorized genes into highly expressed (top quartile) and silent using ovary and testis transcriptome profiling data [32]. We further categorized genes according to whether or not they are *trans*-spliced with the spliced leader (SL) using an SL CAGE dataset [28]. We also categorized genes according to GC content 500 bp upstream of TSSs and defined the top 10% as high GC (HGC) and the bottom 10% as low GC (LGC). We also categorized genes according to their localization on X- and Y-chromosomes or autosomes. For each gene category, we calculated the mean log_2_ ratio at each probe position in a 1000-bp window centered on TSSs and TESs. Genes shorter than 1000-bp and with less than 1100-bp intergenic space were excluded, as were genes with no expression data. A total of 5444 gene models met our criteria for inclusion.

 We used the R package *Ringo* [95] to identify ChIP-enriched regions (chers). This models the distribution of smoothened probe intensities (*y*) as two underlying distributions: a null distribution from non-enriched regions and an alternative distribution from enriched regions. The null distribution is estimated for each sample individually using the empirical distribution of probe intensities: The mode (*m*_0_) is calculated and probe intensities *y* < *m*_*0*_ are reflected onto *y* > *m*_0_. A threshold *y*_0_ is then computed on the null distribution to define enrichment. We computed two enrichment thresholds using the 99 and 95% quantiles of the null distribution estimated for each sample individually. A region was called ChIP-enriched if a minimum of two probes exceeded the enrichment threshold. Regions were merged if the width between them was less than 50 bp. For samples with replicates, we defined final chers by intersecting the chers of each replicate: A region was only classed as ChIP-enriched if it was present in at least two replicates at the 95% threshold and present at the 99% threshold in at least one replicate. For H3K27me3, we also lowered the enrichment threshold to 75% in order to identify broader domains.

### Chromatin states and related genomic features

The genome was segmented into 15 or 50 chromatin states using chromHMM [9]. We created a binary matrix indicating the presence or absence of ChIP-enriched regions for each histone PTM in the ovary and testis, using 50-bp intervals across the genome. A PTM was defined as present if the 50-bp window overlapped a cher. States were learned using this matrix as input to chromHMM. We used models learnt jointly across both ovary and testis for all downstream analyses. We also used chromHMM to learn 15-state models for the ovary and testis data separately. We used chromHMM to calculate fold enrichments of states for various genomic features to functionally classify chromatin states. We used *O. dioica* annotations [25] to define TSS, TES, exon/intron boundaries, TEs and operons. A promoter region was defined as the 400 bp region upstream of the annotated TSS. We used InterPro domains, together with GO terms and manual curation, to identify transcription factors, zinc fingers and homeodomain protein genes. *O. dioica* homeodomain proteins were classified according to whether or not they had DNA-binding specificity PWM predictions using PreMoTF [96] (http://stormo.wustl.edu/PreMoTF/). We calculated the specificity of each *O. dioica* gene across the developmental transcriptome using Shannon entropy, normalized to range between 0 and 1, following [97]. The specificity of a gene ranged from 1 (specific to a single stage) to 0 (equally expressed in all stages). We defined low-specificity and high-specificity genes using thresholds of 0.2 and 0.9, respectively. In order to assign genomic features to chromatin states, we used a threshold of 2 on the enrichments calculated by chromHMM. The main histone modifications for each state were extracted using a threshold of 0.5 on emission parameters.

### GO term analyses

We used published *O. dioica* GO term annotations [32]. A subset of genes was created for each GO term that was associated with 50 or more genes (9665 in total). This subset was further subdivided into active and silent genes for the ovary and testis, separately. Only subsets with 10 or more genes were analyzed further. For each subset, we computed the enrichment of chromatin states at different genomic features (TSS, TES, promoter region and gene body) using chromHMM. Enrichments were clustered hierarchically using (1—the Pearson’s correlation coefficient). Clusters were defined by cutting the resulting dendrogram at a height of 1. Clusters were further defined and functions summarized manually by visual inspection of the respective data files.

## Additional files

**Additional file 1.** Supplementary Results text.

**Additional file 2.** Supplementary Figures S1 to S13 with accompanying legends.

**Additional file 3.** Supplementary Tables S1 to S6 with accompanying footnotes and references.

## Abbreviations

bp: base pair
ChIP-seq: chromatin immunoprecipitation followed by sequencing
CTCF: CCCTC-binding factor
GO term: Gene Ontology term
HCNE: highly conserved element
meDIP: methylated DNA immunoprecipitation
MNase: micrococcal nuclease
PRC: Polycomb repressive complex
RNAPII: RNA polymerase II
TAR: transcriptionally active region
TE: transposable element
TSS: transcription start site
