# Supplementary material for: Sex-specific chromatin landscapes in an ultra-compact chordate genome
Source: Epigenetics Chromatin. 2017 Jan 17;10:3. doi: 10.1186/s13072-016-0110-4 (PMC5240408; doi:10.1186/s13072-016-0110-4)
Supplement: Supplementary file 1 — Additional file 1. Supplementary Results text. [file 13072_2016_110_MOESM1_ESM.pdf]

ADDITONAL FILE 1: Supplemental Results

Sex-specific chromatin landscapes in an ultra-compact chordate genome

P. Navratilova, G. B. Danks, A. Long, S. Butcher, J. R. Manak and E. M. Thompson

Histone PTM distributions and histone modifiers in *Oikopleura dioica*

<u>*Histone acetylations*</u>

Histone acetylations are generally associated with open chromatin and positively influence transcriptional activity by changing the accessibility of protein complexes. This role can also be attributed to *Oikopleura dioica* acetylated histones in the ovary, where pan-histone 4 acetylation matches mostly the pattern of both H3K18 and H3K27 acetylations and tightly correlates with RNAPII distribution (Additional file 2: Figs. S2A,B,C, S8 and S12). This marked active genes with a peak at their promoters. In the testis, however, histone H4ac preferentially associated to genes with GC-rich promoters (HGPs), both active and silent, but was also enriched on active genes on the male X-chromosome (Additional file 2: Fig. S2B,C). In addition, H4ac in the testis did not correlate with RNAPII (Fig. S8 and S12).

We searched for homologs of human histone modifier proteins and validated these using InParanoid [1]. Further, we extracted gene expression values for these homologs from a previously published transcriptomic data set [2] (Additional file 3: Table S6). Curiously, the conserved histone acetyltransferase GCN5/PCAF, a key component of coactivator SAGA (Spt-Ada-Gcn5-acetyltransferase) and ATAC (Ada2a-containing complex) complexes [3] were not identified in the *O. dioica* genome. [3]HAT1, an H4 histone acetyltransferase and key regulator of DNA repair by homologous recombination [4] was also not detected in the genome. This may relate to the reduced DNA repair toolkit and absence of gamma H2A.X histone variant in this species [5,6]. The catalytical component, Myst1/MOF1, of MSL and

NSL complexes, which are general regulators of gene expression, but also crucial for H4ac at the onset of *Drosophila* dosage compensation of the male X-chromosome, and DNA damage response in both mammalian cell and *Drosophila* [7–10], was ubiquitously expressed during *O. dioica* development. Myst2/HBO1, which mediates H4ac for replication origin licensing [11], was highly expressed in the ovary and other endocycling stages indicating that this function may be conserved in *O. dioica* (Fig. 5; Additional file 3: Table S6).

Both H3K18ac and H3K27ac were positively correlated to promoter and gene activity in both samples. They marked larger proportions of the epigenome in the testis (Fig. 1), being enriched at actively transcribed genes (Additional file 2: Fig. S2) although the chromatin state analysis also revealed silent genes bearing H3 acetylations in promoters. According to previously published expression data [2], basic transcriptional machinery- and enhancer-related ubiquitous histone acetyltransferases, ELP3, CBP and p300, responsible for these PTMs, were all ubiquitously expressed throughout *O. dioica* development, and in both ovaries and testes. In this study, we have collated and summarized the raw data available in the cited work, for these, and other histone modifiers, in Fig. 5 and Additional file 3: Table S6.

H3K4 methylations

During transcription, H3K4 methyltransferases associate with the Set1/COMPASS complex and promote H3K4 methylation via interaction with elongating RNAPII [12,13]. *O. dioica* has at least three proteins carrying the Set1/Trithorax-type SET domain, namely Setd1, the major “global” H3K4 di- and tri- methyltransferase common in metazoans and 2 MLL-like (*Drosophila* Trx/Trr) proteins. However, a homolog of MLL1 and another Trx protein, Ash1, which cooperates for the maintenance of homeotic gene expression in response to retinoic acid (RA) during embryonic development in worm, fly and human, has not been identified in the *O.*

dioica genome. This is consistent with loss of the mechanism of RA signalling in *O. dioica* that otherwise operates during chordate development [14]. Interestingly, Setd1 (paralog of SETD1B) was expressed as a male-specific splice variant lacking the SET domain. Further, there is a testis-specific H3K4- and H3K36- tri-methyltransferase in *O. dioica* homologous to human PRDM9, reported to mark crossover hotspots during meiotic recombination in mammals [15,16], that is also involved in transgenerational inheritance in the male germ line [17].

H3K4me1 was not very abundant in *O. dioica* gonads (Fig. 1) but was slightly more enriched on active genes of the X-chromosome (Additional file 2: Fig. S2C). In contrast, H3K4 di-methylation was strongly associated with gene activation and progressively declined towards the ends of active genes. We observed strong enrichment of H3K4me2 on all low-GC (LGP) and expressed high-GC promoters (HGP) in the ovary. In contrast, silent HGP promoters were depleted for this mark (Additional file 2: Fig. S2B). The difference between H3K4me2 on HGP and LGP was not as apparent in the testis. H3K4me3 is a histone PTM defining promoters and its ChIP signal peaks around the TSS of actively expressed genes in all eukaryotes examined to date [18,19] (Table 1). Similarly, a large proportion of active *O. dioica* promoters was enriched in H3K4me3. In active operons in the ovary H3K4me3 peaks were only detected at the promoter of the most 5` gene and not ahead of the internal operon cistrons. The H3K4me3 peak was apparent on both expressed and silent LGP and generally depleted from HGP in the testis (Additional file 2: Fig. S2B). Testis Y-chromosomal regions, generally transcriptionally silent, were also broadly enriched with H3K4me3 (Fig. 1; Additional file 2: Fig. S2D).

H3K9 methylations

Plotting H3K9me1 and H3K9me2 distributions revealed differences between ovary and testes samples and the sex chromosomes. Mono-methylation was associated with active promoters and gene bodies in the ovary, but was depleted on these in the

testis autosomal regions. Instead it was deposited on promoters of X-chromosomal genes in particular (Additional file 2: Fig. S2C). EHMT1 and 2 (Glp1 and G9a respectively) that act in a heterodimeric complex mainly as H3K9 mono- and di-methyltransferases for euchromatin [20], belonged to the ovary-specific group of histone modifiers (Fig. 5).

H3K9me3 is a proxy for identifying heterochromatic domains that are key for gene regulation, genome organization, genome stability and chromosome inheritance. It creates an interaction site for HP1 and is deposited by Suv39H1,2 and Setdb1 which belong to the complex found to interact with the mono- and di-methyltransferases EHMT1 and 2 [21]. Suv39H1 was ubiquitously expressed in *O. dioica*, whereas the other two, Suv39H2 and Setdb1 were silent in the testis, but abundant in the ovary (Fig. 5, Additional file 3: Table S6). Despite the presence of only one DNA (and/or RNA) methyltransferase (DNMT2) in *O. dioica*, H3K9me3 was strongly correlated with methylated DNA in the testis. This is well established from other organisms with high levels of DNA methylation and full DNMT toolkits, and is due to the interaction of the histone methyltransferase complex with both *de novo* and maintenance DNA methyltransferases [22]. Di- and tri-methylation of H3K9 were generally highly enriched in the testis, particularly on the Y-chromosome (Fig. 1), which represents a typical repressive mark of TE-rich genomic sequences (Fig. 2C).

H3K27 methylations

H3K27me1 enrichment patterns correlated with other transcription-related mono-methylations (H3K36me1, H3K20me1, H3K79me1) at active intragenic regions in the ovary (Additional file 2: Fig. S8). We observed relatively weak enrichments of H3K27me1 in the testis. In accordance to its profile in *C. elegans* [23], H3K27me1 was more enriched on the male X-chromosome in contrast to its relatively higher enrichment on autosomes in the ovary, whereas H3K27me3 was more abundant on X-chromosome in both sexes (Additional file 2: Fig. S2C). H3K27me1 clustered with

H3K36me3 and H3K27me3 in the testis correlation heatmap (Additional File 2: Fig. S8). This resembles findings that PRC2-deposited H3K27me1 accumulates within transcribed genes and is regulated by H3K36me3 deposition as the first step towards epigenetic memory of cell-type specific gene expression states [24]. H3K27me3 is deposited by the PRC2 complex which provides cellular memory of repressed states of developmentally regulated genes [25]. In *O. dioica*, the repressive function is conserved and H3K27me3 accumulates on the X chromosome in both ovary and testes, suggesting a role in female X dosage compensation (Additional file 2: Fig. S2C). Catalytic subunits (EZH1) of PRC2 and 2 paralogs of mammalian di- and tri-methyltransferases of mono-methylated K27 were highly expressed in the ovary and only EZH2 was weakly expressed in the testis (Fig. 5; Additional file 3: Table S6).

H3K36 methylations

H3K36 methylations are typically coupled to transcriptional elongation and have roles in transcriptional termination and early RNA processing. H3K36me is distributed over gene bodies with a progressive shift from mono- to tri-methylation of H3K36 between the promoters and 3′ ends of genes [26]. Mono-methylation only weakly correlates with transcriptional elongation and exhibits preference for genes with CpG-island promoters in mammalian cells [27,28]. These characteristics fit the distribution of H3K36me1 in *O. dioica*, which shows relatively low enrichment overall, shifted towards the 5' of genes, with peaks predominantly at active HGPs (Additional file 2: Fig. S2B). This is also reflected in the 50-state model, where H3K36me1-enriched states 38 and 39 appear specific for TF, ZF and HD genes (Additional file 2: Fig. S5).

High enrichments of H3K36me2 were detected on actively transcribed *O. dioica* genes with large continuous blocks covering gene bodies and correlating with RNAPII occupancy. In metazoans, H3K36me2 is uniformly distributed across transcribed genes and it is the most frequent histone mark in the *O. dioica* ovary sample: the ChIP enriched regions cover about 30% of the genome (Fig. 1). This

mark correlated with H3K36me3 (Additional file 2: Figs. S8 and S12) with higher enrichment at SL-genes that tend to occur in operons (Additional file 2: Fig. S2A). Mono- and di-methylations are deposited by the NSD-1 methyltransferase, independently from SETD2-deposited H3K36me3. Interestingly, another H3K36-methyltransferase NSD-1 was expressed in both ovary and testis, whereas SETD2 was silent in the testis (Fig. 5, Additional file 3: Table S6). Another alternative to substitute SETD2 in its histone methyltransferase activity is PRDM9, with recently confirmed specificity towards both H3K4me3 and H3K36me3 [16]. Intriguingly, H3K36me3-marked silent genomic regions were frequently found on the Y-chromosome in combination with H3K4me3 and heterochromatic marks (Additional file 2: Figs. S2C, S8 and S12).

H3K79 and H4K20 methylations

Methylations of H3K79me and H4K20me are known to be involved in cell cycle regulation as well as heterochromatin formation [29,30]. There was generally low enrichment of methylations on these residues in genic regions of the testis. This was not related to transcriptional activity and may be a consequence of asynchronous mitotic cell cycling in this tissue, as such a distribution is similar to what has been previously reported [31]. In the actively transcribing, endocycling ovary, however, H3K79me1 associated with active transcription, in agreement with studies on CD4+ cells [32,33]. Unlike the CD4+ model system, there was no dependence in *O. dioica* on the GC-content of target gene promoters (Additional file 2: Fig. S2B). H3K79me3 I s found at silent telomeric and centromeric regions but is also enriched on transcribed regions, independent of transcription level, in *S. cerevisiae* [34], *C. elegans* [23] and human cells [33,35]. It is deposited in a non-processive manner as a consequence of Dot1 (the sole H3K79 methyltransferase) association with the elongation complex [36]. It modulates the level of RNAPII transcription on essential ubiquitously expressed genes and contributes to RNAPII pausing on essential genes

during development and stress response [37,38]. This mark has also been proposed to provide the cell with a timer mechanism to couple cell-cycle length to changes in chromatin [39]. In *O. dioica* testis and ovary, ChIP distributions around gene starts and ends show mild H3K79me3 association with silent genes in the testis versus relatively highly enriched with no preference towards active or silent regions in the ovary (Additional file 2: Fig. S2). Dot1 was expressed ubiquitously, both in the testes and ovary (Fig. 5, Additional file 3: Table S6).

H4K20me1 represses a subset of mammalian genes [40] and marks the human Xist-expressing X chromosome in ES cells [41]. In *C. elegans* the dosage compensation complex (DCC) controls higher H4K20me1, versus lower H4K20me3 and H4ac levels, on the X chromosomes of XX hermaphrodites relative to autosomes, as a part of the two-fold repression of X-linked genes [42,43]. Other studies correlate this mark with active transcription [31,32]. H4K20me1 distribution in *O. dioica* highly correlated with H3K79me1 in the ovary (Additional file 2: Fig. S8) and was enriched on active genes with no dependence on sex chromosome location. SETD8, the sole enzyme essential for H4K20 mono-methylation, is cell cycle regulated. It is implicated in chromosome condensation during mitosis, is required for proper cytokinesis [44], and has a role in replication origin licensing for replication complex loading [45]. Another role is to regulate RNAPII pausing by controlling H4K16Ac and H4K20me3 deposition by Suv420H2 [46]. The *O. dioica* homolog of SETD8 is expressed in both ovary and testis. The other H4K20 methyltransferases Suv420H1 and 2 are both expressed in the ovary, whereas in testis, Suv420H2 is weakly expressed and the di-methylating counterpart, Suv420H1, is silent (Fig. 5, Additional file 3: Table S6). H4K20me3 is a histone PTM associated with transcription inhibition, located typically in centromeres, telomeres and other constitutive heterochromatin [47]. This PTM likely marks constitutive heterochromatin in *O. dioica* as H4K20me3 distributions correlate tightly to H3K9me3 in both tissues.

H3S28P

H3S28P, another key epigenetic player in cell cycle control with links to chromatin condensation during mitosis and meiosis, was distributed differently in *O. dioica* ovary and testis. We observed low-enriched broad regions, overlapping both silent and actively transcribed gene bodies and correlated to H3K27me3 and H3K4me1 in the ovary (Additional file 2: Figs. S8, S11 and S12). Enrichment in both active and silent regions was higher on the X-chromosome in testes (Additional file 2: Fig. S2C) and showed frequent coincidence with CTCF and p300 (Additional file 2: Fig. S8). Recent data support H3S28P promoting Polycomb-mediated gene silencing [48]. There is also evidence of a role in gene activation via stress-induced phosphorylation at S28 by MSK1/2 to reduce association of histone deacetylases and locally increase histone acetylation [49]. The two *O. dioica* MSK paralogs were ubiquitously expressed. In yeast and metazoa, histone H3 S10 and S28 are temporarily phosphorylated by Aurora B kinase during mitotic chromatin condensation. This also occurs in *O. dioica* during the mitotic cell cycle mode in early development and in the testis. Interestingly, the PP1-alpha catalytic subunit is duplicated in *O. dioica* with one testis-specific paralog (Fig. 5, Additional file 3: Table S6).

In summary, the distributions and expression patterns of the histone modifiers in the ovary and testis of *O. dioica* reflect different requirements in the mitotic versus endocycling tissues that may prioritize specific subsets of their multiple functions.

E2F1 and E2F7 binding sites

E2F activator and repressor transcription factors are known to antagonize each other by oppositely regulating an overlapping set of genes [50]. To explore such relations in *O. dioica*, we measured their degree of overlap. ChIP enriched regions (chers) of the activator E2F1 and repressor E2F7 showed high (45% as maximum) spatial and temporal co-localization (not shown). This indicates that there is indeed a high degree of overlap between the genomic regions bound and the sets of genes

regulated. Among genes that were targets of both TFs within one tissue, a significant overrepresentation of genes whose biological processes were associated to DNA replication and nucleotide metabolism was found. Other prominent overrepresented groups of GO terms among these genes were related with cell cycle regulation and progression, formation of protein-DNA complexes (with TFs overrepresented among these) and processes related with development of adult traits.

The 50-state chromatin model resolves additional features

In addition to the 15-state model we generated a 50-state model learned from pooled ovarian and testis histone PTM data (Additional file 2: Fig. S5). Some of the states from the 15-state model were resolved into multiple sub-states (Additional file 3: Table S5) and differentiated between silent versus active groups of genes. For example states 29-32 (state 8 in the 15-state model) showed that only state 32 marks silent genes and that states 29 and 30 were located as double peaks around TSS of distinct subgroups of genes. Similarly, states 15-20 (state 11 in the 15-state model) were resolved between state 18 with H3K4me3 downstream of transposable element TSSs, state 19, frequent on the Y-chromosome and state 20 as a non-transposable element repressive state. Expansion of the number of states was also helpful in better definition of developmental gene signatures. H4ac was frequently associated with developmental genes that were silent in the testis. Also in the testis, state 14 included H4K20me1 on the promoters of silent genes coding for TFs, HDs and ZFs. Finally, state 50 represents a number of testis-specific H3S28P, CTCF and p300 enriched regions, most frequently occurring on the X-chromosome.

Supplemental References

1. Sonnhammer ELL, Östlund G. InParanoid 8: orthology analysis between 273 proteomes, mostly eukaryotic. Nucleic Acids Res. 2015;43:D234-9.

2. Danks G, Campsteijn C, Parida M, Butcher S, Doddapaneni H, Fu B, et al.

OikoBase: a genomics and developmental transcriptomics resource for the urochordate Oikopleura dioica. Nucleic Acids Res. 2013;41:D845-53.

3. Spedale G, Timmers HTM, Pijnappel WWMP. ATAC-king the complexity of SAGA during evolution. Genes Dev. 2012;26:527–41.

4. Yang X, Li L, Liang J, Shi L, Yang J, Yi X, et al. Histone acetyltransferase 1 promotes homologous recombination in DNA repair by facilitating histone turnover. J. Biol. Chem. 2013;288:18271–82.

5. Denoeud F, Henriet S, Mungpakdee S, Aury J-M, Da Silva C, Brinkmann H, et al. Plasticity of animal genome architecture unmasked by rapid evolution of a pelagic tunicate. Science. 2010;330:1381–5.

6. Moosmann A, Campsteijn C, Jansen PW, Nasrallah C, Raasholm M, Stunnenberg HG, et al. Histone variant innovation in a rapidly evolving chordate lineage. BMC Evol. Biol. 2011;11:208.

7. Hilfiker A, Hilfiker-Kleiner D, Pannuti A, Lucchesi JC. mof, a putative acetyl transferase gene related to the Tip60 and MOZ human genes and to the SAS genes of yeast, is required for dosage compensation in Drosophila. EMBO J. 1997;16:2054–60.

8. Bhadra MP, Horikoshi N, Pushpavallipvalli SNCVL, Sarkar A, Bag I, Krishnan A, et al. The role of MOF in the ionizing radiation response is conserved in Drosophila melanogaster. Chromosoma. 2012;121:79–90.

9. Kind J, Vaquerizas JM, Gebhardt P, Gentzel M, Luscombe NM, Bertone P, et al. Genome-wide analysis reveals MOF as a key regulator of dosage compensation and gene expression in Drosophila. Cell. 2008;133:813–28.

10. Smith ER, Cayrou C, Huang R, Lane WS, Côté J, Lucchesi JC. A human protein complex homologous to the Drosophila MSL complex is responsible for the majority of histone H4 acetylation at lysine 16. Mol. Cell. Biol. 2005;25:9175–88.

11. Iizuka M, Stillman B. Histone Acetyltransferase HBO1 Interacts with the ORC1 Subunit of the Human Initiator Protein. J. Biol. Chem. 1999;274:23027–34.

12. Wang P, Lin C, Smith ER, Guo H, Sanderson BW, Wu M, et al. Global analysis of H3K4 methylation defines MLL family member targets and points to a role for MLL1-mediated H3K4 methylation in the regulation of transcriptional initiation by RNA polymerase II. Mol. Cell. Biol. 2009;29:6074–85.
13. Miller T, Krogan NJ, Dover J, Erdjument-Bromage H, Tempst P, Johnston M, et al. COMPASS: a complex of proteins associated with a trithorax-related SET domain protein. Proc. Natl. Acad. Sci. U. S. A. 2001;98:12902–7.
14. Cañestro C, Postlethwait JH. Development of a chordate anterior-posterior axis without classical retinoic acid signaling. Dev. Biol. 2007;305:522–38.
15. Grey C, Barthès P, Chauveau-Le Friec G, Langa F, Baudat F, de Massy B. Mouse PRDM9 DNA-binding specificity determines sites of histone H3 lysine 4 trimethylation for initiation of meiotic recombination. PLoS Biol. 2011;9:e1001176.
16. Powers NR, Parvanov ED, Baker CL, Walker M, Petkov PM, Paigen K. The Meiotic Recombination Activator PRDM9 Trimethylates Both H3K36 and H3K4 at Recombination Hotspots In Vivo. PLoS Genet. 2016;12:e1006146.
17. Greer EL, Beese-Sims SE, Brookes E, Spadafora R, Zhu Y, Rothbart SB, et al. A histone methylation network regulates transgenerational epigenetic memory in C. elegans. Cell Rep. 2014;7:113–26.
18. Bernstein BE, Humphrey EL, Erlich RL, Schneider R, Bouman P, Liu JS, et al. Methylation of histone H3 Lys 4 in coding regions of active genes. Proc. Natl. Acad. Sci. U. S. A. 2002;99:8695–700.
19. Lauberth SM, Nakayama T, Wu X, Ferris AL, Tang Z, Hughes SH, et al. H3K4me3 interactions with TAF3 regulate preinitiation complex assembly and selective gene activation. Cell. 2013;152:1021–36.
20. Tachibana M, Ueda J, Fukuda M, Takeda N, Ohta T, Iwanari H, et al. Histone methyltransferases G9a and GLP form heteromeric complexes and are both crucial for methylation of euchromatin at H3-K9. Genes Dev. 2005;19:815–26.
21. Fritsch L, Robin P, Mathieu JRR, Souidi M, Hinaux H, Rougeulle C, et al. A

subset of the histone H3 lysine 9 methyltransferases Suv39h1, G9a, GLP, and SETDB1 participate in a multimeric complex. Mol. Cell. 2010;37:46–56.

22. Lehnertz B, Ueda Y, Derijck AAHA, Braunschweig U, Perez-Burgos L, Kubicek S, et al. Suv39h-Mediated Histone H3 Lysine 9 Methylation Directs DNA Methylation to Major Satellite Repeats at Pericentric Heterochromatin. Curr. Biol. 2003;13:1192–200.

23. Gerstein MB, Lu ZJ, Van Nostrand EL, Cheng C, Arshinoff BI, Liu T, et al. Integrative analysis of the Caenorhabditis elegans genome by the modENCODE project. Science. 2010;330:1775–87.

24. Ferrari KJ, Scelfo A, Jammula S, Cuomo A, Barozzi I, Stützer A, et al. Polycomb-dependent H3K27me1 and H3K27me2 regulate active transcription and enhancer fidelity. Mol. Cell. 2014;53:49–62.

25. Cao R, Wang L, Wang H, Xia L, Erdjument-Bromage H, Tempst P, et al. Role of histone H3 lysine 27 methylation in Polycomb-group silencing. Science. 2002;298:1039–43.

26. Bannister AJ, Schneider R, Myers FA, Thorne AW, Crane-Robinson C, Kouzarides T. Spatial distribution of di- and tri-methyl lysine 36 of histone H3 at active genes. J. Biol. Chem. 2005;280:17732–6.

27. Andersson R, Enroth S, Rada-Iglesias A, Wadelius C, Komorowski J. Nucleosomes are well positioned in exons and carry characteristic histone modifications. Genome Res. 2009;19:1732–41.

28. Vavouri T, Lehner B. Human genes with CpG island promoters have a distinct transcription-associated chromatin organization. Genome Biol. 2012;13:R110.

29. Beck DB, Oda H, Shen SS, Reinberg D. PR-Set7 and H4K20me1: at the crossroads of genome integrity, cell cycle, chromosome condensation, and transcription. Genes Dev. 2012;26:325–37.

30. Kim W, Choi M, Kim J-E. The histone methyltransferase Dot1/DOT1L as a critical regulator of the cell cycle. Cell Cycle. 2014;13:726–38.

31. Barski A, Cuddapah S, Cui K, Roh T-Y, Schones DE, Wang Z, et al. High-resolution profiling of histone methylations in the human genome. Cell. 2007;129:823–37.

32. Karlić R, Chung H-R, Lasserre J, Vlahovicek K, Vingron M. Histone modification levels are predictive for gene expression. Proc. Natl. Acad. Sci. U. S. A. 2010;107:2926–31.

33. Wang Z, Zang C, Rosenfeld J a, Schones DE, Barski A, Cuddapah S, et al. Combinatorial patterns of histone acetylations and methylations in the human genome. Nat. Genet. 2008;40:897–903.

34. Pokholok DK, Harbison CT, Levine S, Cole M, Hannett NM, Lee TI, et al. Genome-wide map of nucleosome acetylation and methylation in yeast. Cell. 2005;122:517–27.

35. Ernst J, Kheradpour P, Mikkelsen TS, Shoresh N, Ward LD, Epstein CB, et al. Mapping and analysis of chromatin state dynamics in nine human cell types. Nature. 2011;473:43–9.

36. Wood A, Schneider J, Dover J, Johnston M, Shilatifard A. The Paf1 complex is essential for histone monoubiquitination by the Rad6-Bre1 complex, which signals for histone methylation by COMPASS and Dot1p. J. Biol. Chem. 2003;278:34739–42.

37. Cecere G, Hoersch S, Jensen MB, Dixit S, Grishok A. The ZFP-1(AF10)/DOT-1 complex opposes H2B ubiquitination to reduce Pol II transcription. Mol. Cell. 2013;50:894–907.

38. Frederiks F, Tzouros M, Oudgenoeg G, van Welsem T, Fornerod M, Krijgsveld J, et al. Nonprocessive methylation by Dot1 leads to functional redundancy of histone H3K79 methylation states. Nat. Struct. Mol. Biol. 2008;15:550–7.

39. De Vos D, Frederiks F, Terweij M, van Welsem T, Verzijlbergen KF, Iachina E, et al. Progressive methylation of ageing histones by Dot1 functions as a timer. EMBO Rep. 2011;12:956–62.

40. Congdon LM, Houston SI, Veerappan CS, Spektor TM, Rice JC. PR-Set7-

mediated monomethylation of histone H4 lysine 20 at specific genomic regions induces transcriptional repression. J. Cell. Biochem. 2010;110:609–19.

41. Kohlmaier A, Savarese F, Lachner M, Martens J, Jenuwein T, Wutz A. A chromosomal memory triggered by Xist regulates histone methylation in X inactivation. PLoS Biol. 2004;2:E171.

42. Wells MB, Snyder MJ, Custer LM, Csankovszki G. Caenorhabditis elegans dosage compensation regulates histone H4 chromatin state on X chromosomes. Mol. Cell. Biol. 2012;32:1710–9.

43. Vielle A, Lang J, Dong Y, Ercan S, Kotwaliwale C, Rechtsteiner A, et al. H4K20me1 contributes to downregulation of X-linked genes for C. elegans dosage compensation. Kelly WG, editor. PLoS Genet. 2012;8:e1002933.

44. Oda H, Okamoto I, Murphy N, Chu J, Price SM, Shen MM, et al. Monomethylation of histone H4-lysine 20 is involved in chromosome structure and stability and is essential for mouse development. Mol. Cell. Biol. 2009;29:2278–95.

45. Tardat M, Brustel J, Kirsh O, Lefevbre C, Callanan M, Sardet C, et al. The histone H4 Lys 20 methyltransferase PR-Set7 regulates replication origins in mammalian cells. Nat. Cell Biol. 2010;12:1086–93.

46. Kapoor-Vazirani P, Vertino PM. Dual role of PR-SET7-mediated H4K20me1 in the local regulation of RNA polymerase II promoter-proximal pausing. J. Biol. Chem. 2014;

47. Beck DB, Burton A, Oda H, Ziegler-Birling C, Torres-Padilla M-E, Reinberg D. The role of PR-Set7 in replication licensing depends on Suv4-20h. Genes Dev. 2012;26:2580–9.

48. Yung PYK, Stuetzer A, Fischle W, Martinez A-M, Cavalli G. Histone H3 Serine 28 Is Essential for Efficient Polycomb-Mediated Gene Repression in Drosophila. Cell Rep. 2015;

49. Sawicka A, Hartl D, Goiser M, Pusch O, Stocsits RR, Tamir IM, et al. H3S28 phosphorylation is a hallmark of the transcriptional response to cellular stress.

Genome Res. 2014;24:1808–20.

50. Pandit SK, Westendorp B, Nantasanti S, van Liere E, Tooten PCJ, Cornelissen PW a, et al. E2F8 is essential for polyploidization in mammalian cells. Nat. Cell Biol. 2012;14:1181–91.
