# Supplementary material for: Sex-specific chromatin landscapes in an ultra-compact chordate genome
Source: Epigenetics Chromatin. 2017 Jan 17;10:3. doi: 10.1186/s13072-016-0110-4 (PMC5240408; doi:10.1186/s13072-016-0110-4)
Supplement: Supplementary file 2 — Additional file 2. Supplementary Figures S1 to S13 with accompanying legends. [file 13072_2016_110_MOESM2_ESM.pdf]

ADDITIONAL FILE 2: Supplemental Figures S1-13

Sex-specific chromatin landscapes in an ultra-compact chordate genome

Pavla Navratilova[1§], Gemma B. Danks[1§], Abby Long[2], Stephen Butcher[2], J. Robert Manak[2] and Eric M. Thompson[1,3]*

Supplemental Figure S1

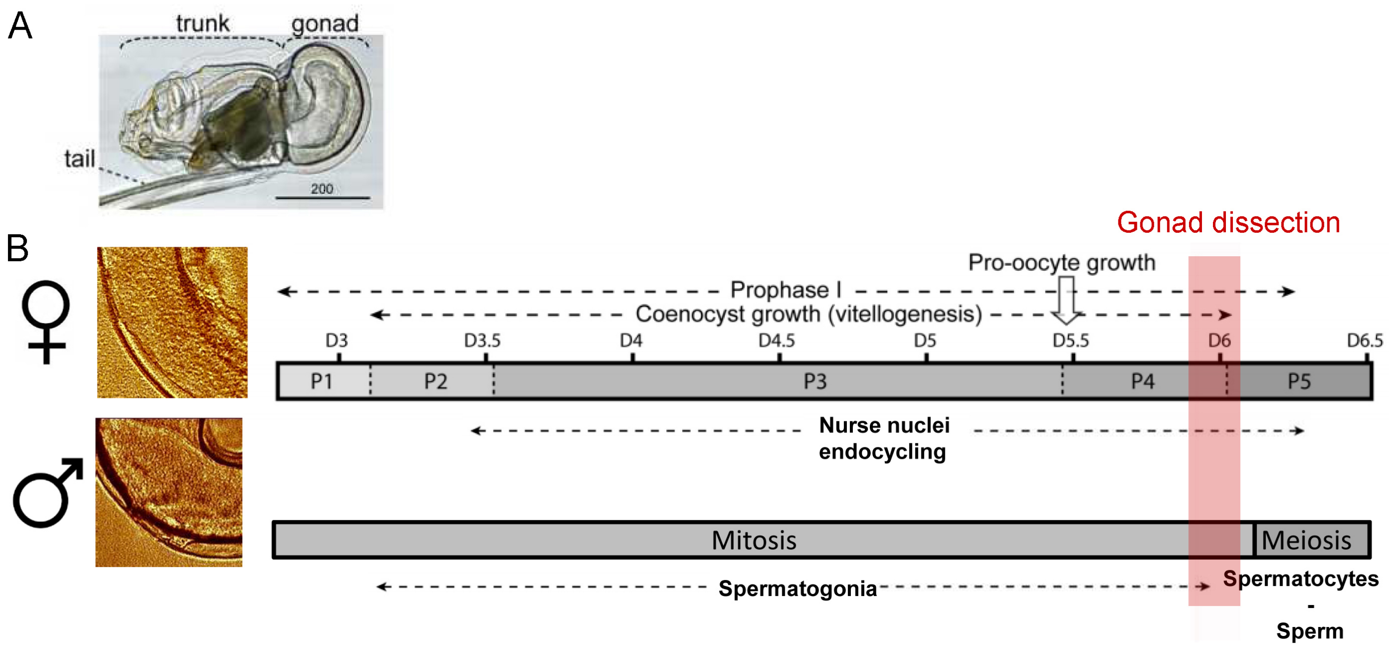

Supplemental Fig. S1. Specification of the tissues sampled in this study. A) *Oikopleura dioica* in the late day 5 - early day 6 developmental stage that was used for all experiments. Scale bar in µm. B) Gonad maturation chronologies in the ovaries (meiotic nuclei - top, nurse nuclei - bottom) and in the testis. The sexes can be easily separated based on the size of nuclei (larger endocyling nuclei give a rougher appearance in the ovary) as shown by the stereoscope images of day 5-6 ovary and testis sections. The stages that were sampled are indicated by a red rectangle on the timeline.

Supplemental Figure S2

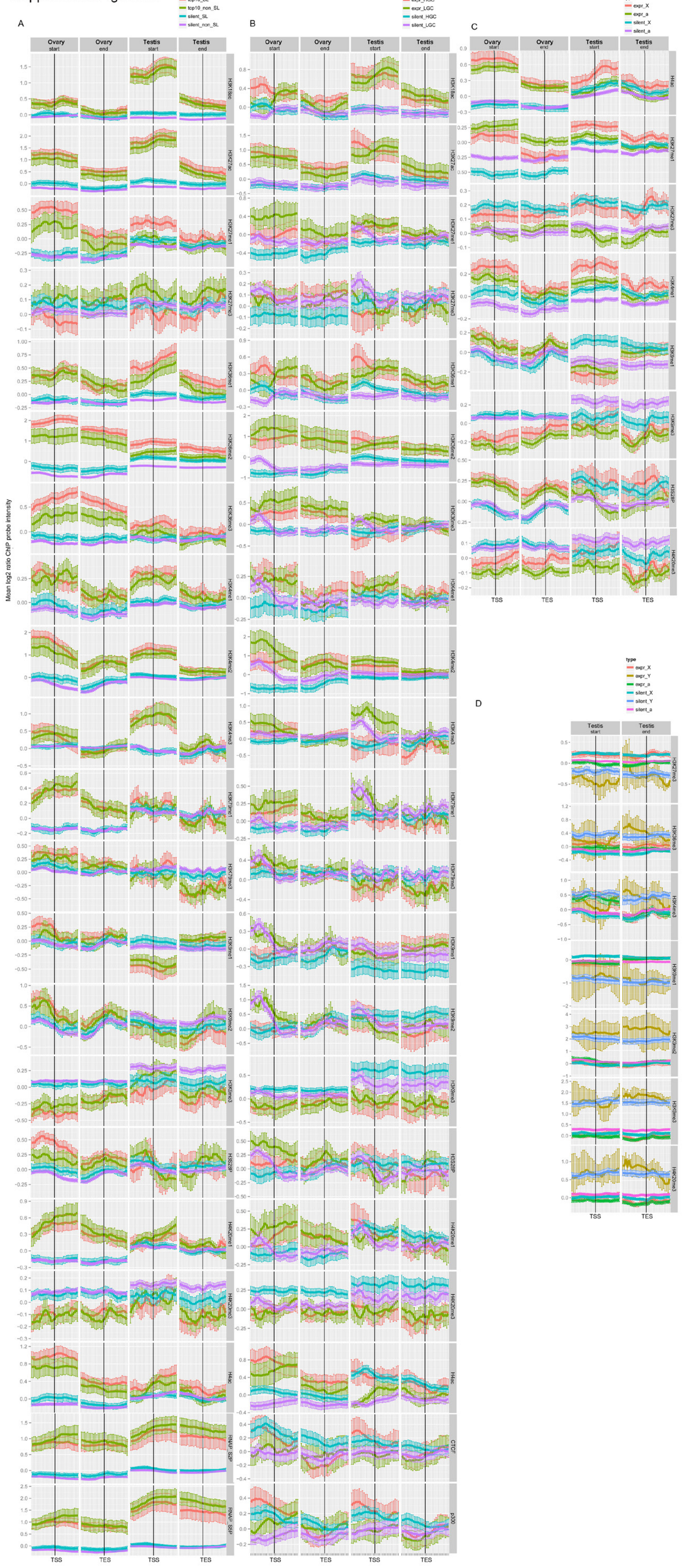

Supplemental Fig. S2. Mean $\log_2$ ChIP to input signals in a 1000 bp window centered around transcription start (TSS) and end (TES) sites for different gene categories. Genes were grouped by expression level (upper quartile of expression versus silent) and further subdivided based on whether or not their transcripts are *trans*-spliced with the spliced leader SL-RNA (A); the GC-content of their promoter regions: high GC = HGP (top 10% of all genes) and low GC = LGP (bottom 10% of all genes) (B); or by their chromosomal location (X, Y, or autosomal) (C,D). Error bars represent 95% confidence intervals on the mean calculated by bootstrapping.

Supplemental Figure S3

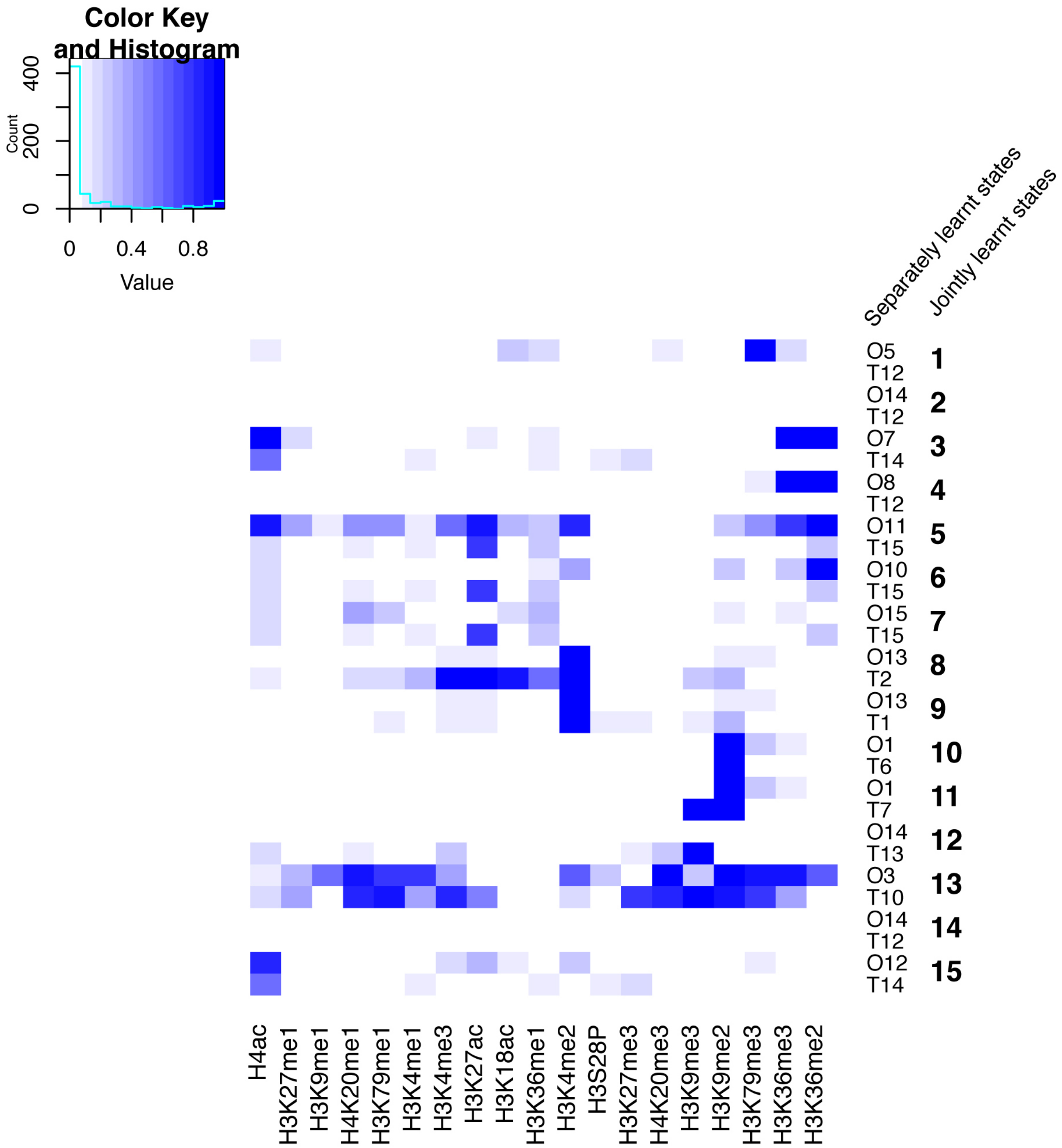

Supplemental Fig. S3. Emission parameters from separately learnt models on the ovary and testis samples were compared to those of the jointly learnt model on both tissues combined. States from the separately learnt models as labeled (where O# = state # in the ovary model and T# = state # in the testis model) according to their closest match (lowest Euclidean distance) to the jointly learnt model states (1 to 15). The jointly learnt model captures all states of the separately learnt models shown here (cf. Fig. 2A).

Supplemental Figure S4

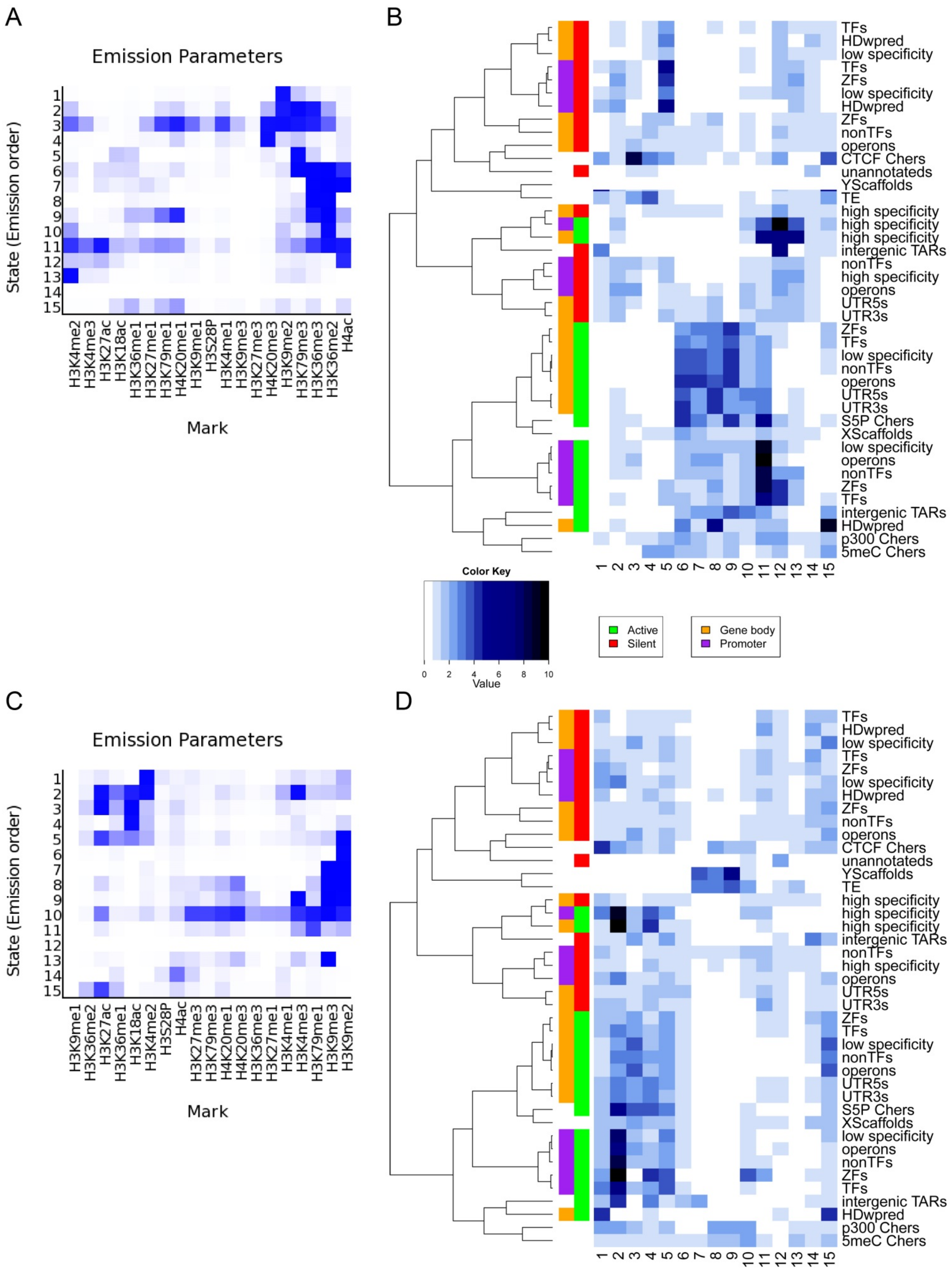

Supplemental Fig. S4. Chromatin states in the *O. dioica* ovary and testis obtained by learning 15-state models separately for each data set reveal all states captured by the jointly learnt model and highlight their specificity to each tissue. Heatmaps visualize model emission parameters from the ovary (A) and testis (C) 15-state models (see also Fig. S3). The fold-enrichments at genomic features for separately learnt chromatin state in the ovary (B) and testis (D) are plotted as in Fig. 2. Gene bodies (orange) and promoter regions (purple) are split into active (green) and silent (red). Txn = transcription; TF = transcription factor; ZF = zinc finger protein; HD = homeodomain protein; high and low specificity genes according to breadth of expression across development (see Methods); TAR = transcriptionally active region; TE = transposable element; unannotated = regions lacking annotation or transcription; 5meC = methylcytosine; S5P = serine 5 phosphorylated RNA polymerase II.

Supplemental Figure S5

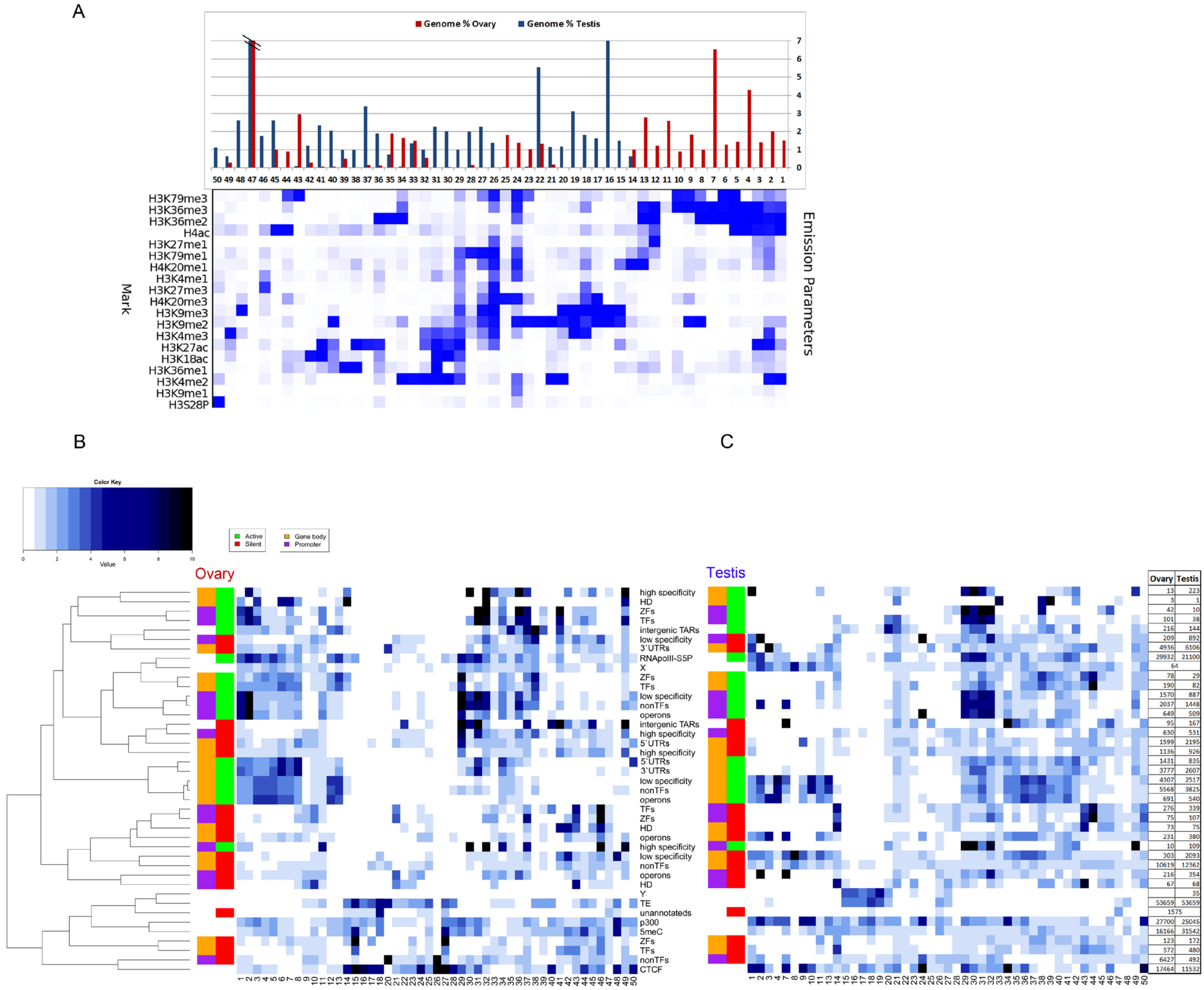

Supplemental Fig. S5. A more detailed 50-state model for the *O. dioica* ovary and testes epigenomes. A) Heatmap of model emission parameters for 50 chromatin states learned across the genome, using both samples. Proportions of the genome in the ovary and testis covered by each chromatin state are indicated at the top. Chromatin state fold-enrichments for genomic features in the ovary (B) and testis (C), ordered according to clustering by correlation in the ovary. The table gives the numbers of each feature in each sample. Gene bodies (orange) and promoter regions (purple) are split into active (green) and silent (red). Txn = transcription; TF = transcription factor; ZF = zinc finger protein; HDwpred = homeodomain protein; high and low specificity genes according to breadth of expression across development (see Methods); TAR = transcriptionally active region; TE = transposable element; unannotated = regions lacking annotation or transcription; Cher = ChIP-enriched region; 5meC = methylcytosine; S5P = serine 5 phosphorylated RNA polymerase II; UTR5 = 5’ UTR.

Supplemental Figure S6

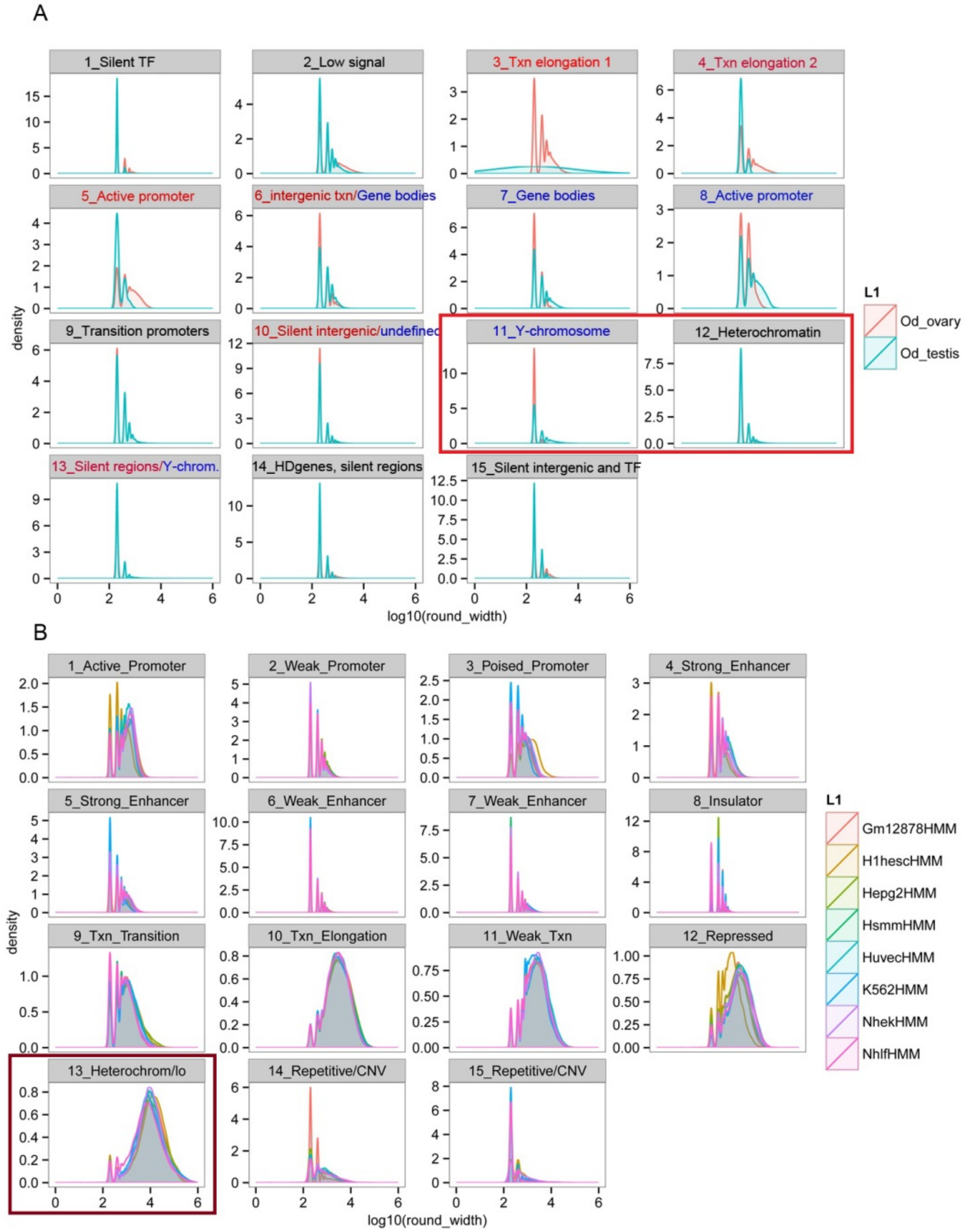

Supplemental Fig. S6. Compact chromatin state domains in the *O. dioica* epigenome. Distributions of chromatin state domain widths are shown for each of 15 chromatin states in the testis and ovary in *O. dioica* (A) and in 8 human cell lines (B) [1]. The heterochromatic states (red boxes) are reduced in particular compared to corresponding states in the human epigenome.

Supplemental Figure S7

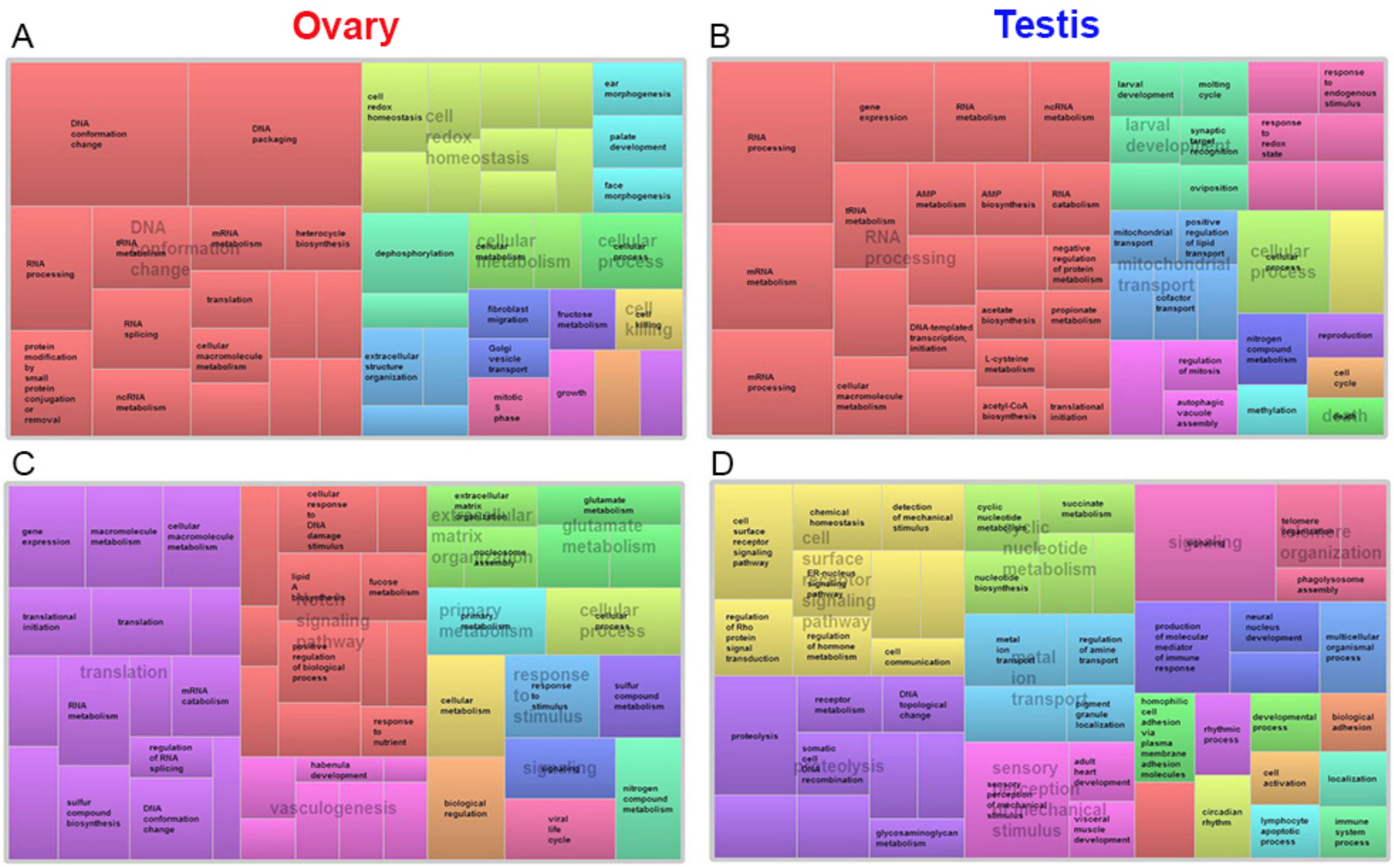

Supplemental Fig. S7. Gene Ontology (GO) categories related to RNA pol II pausing and H3K27me3 ChIP-enriched regions. GO-terms over-represented in the set of silent genes with paused RNA pol II in the promoter in the ovary (A) and testis (B). GO terms over-represented in the set of genes with H3K27me3 chers in the ovary (C) and (D) testis.

Supplemental Figure S8

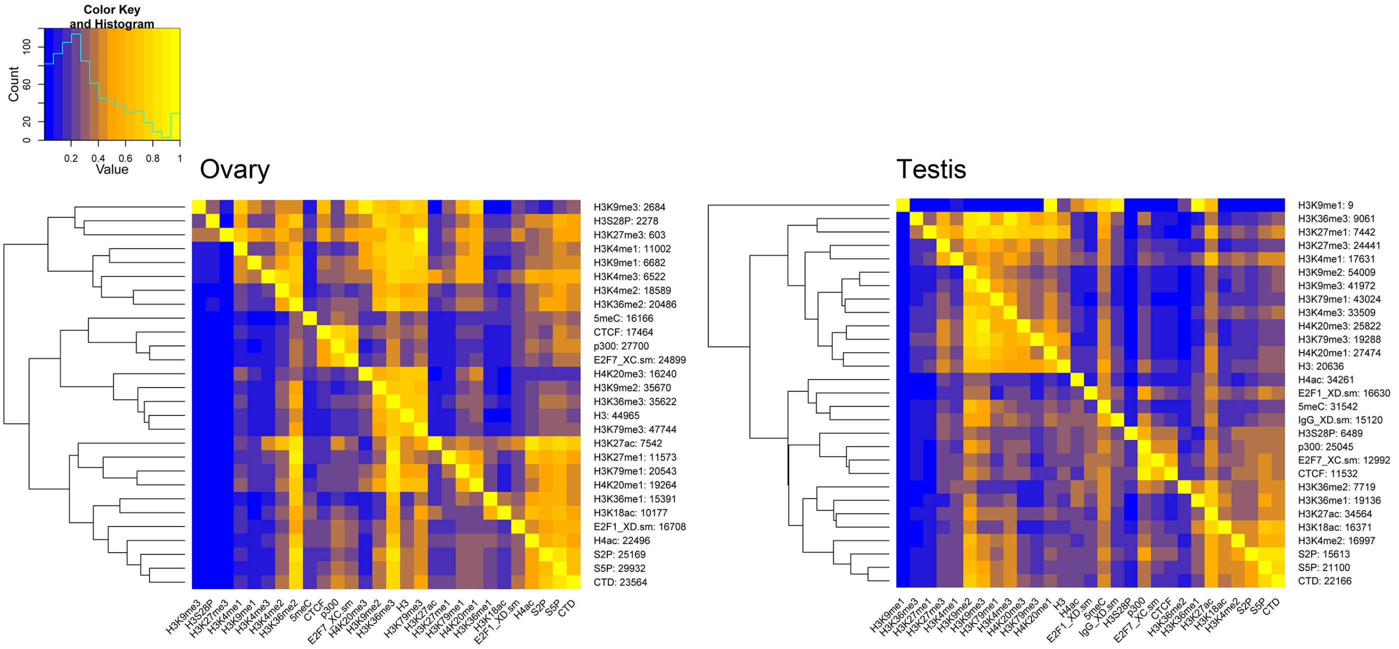

Supplemental Fig. S8. Co-occurrence of ChIP-enriched regions among all ChIP samples in both testis and ovary. Labels indicate the sample and the number of the particular ChIP-enriched regions (chers) detected (using all replicates; see Methods) in the ovary and testes, respectively. Color indicates the proportion of overlapping chers between pairs of samples (blue = 0% overlap; yellow = 100% overlap).

Supplemental Figure S9

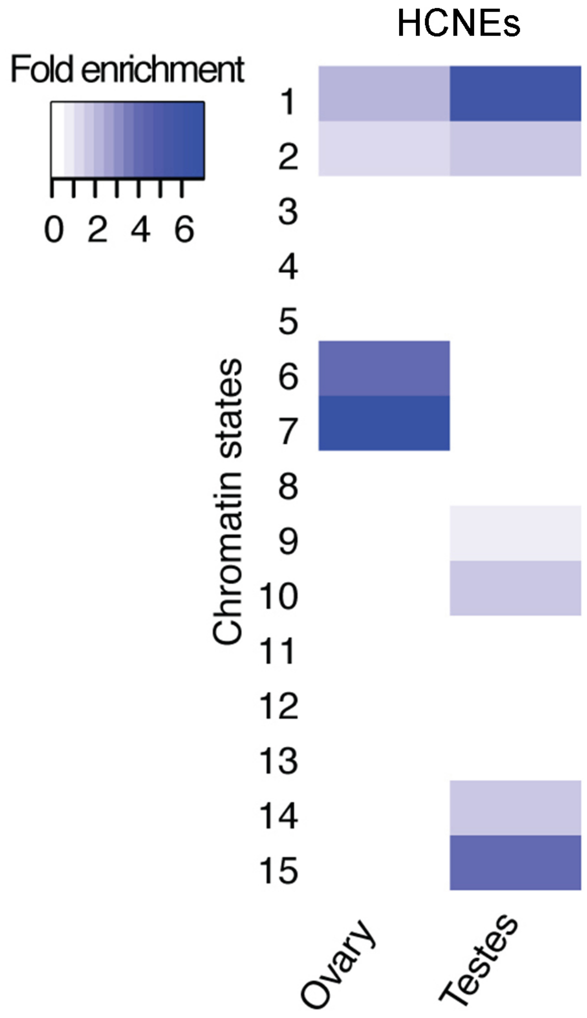

Supplemental Fig. S9. Enrichment of 15 jointly-learnt chromatin states at HCNEs (highly conserved elements).

Supplemental Figure S10

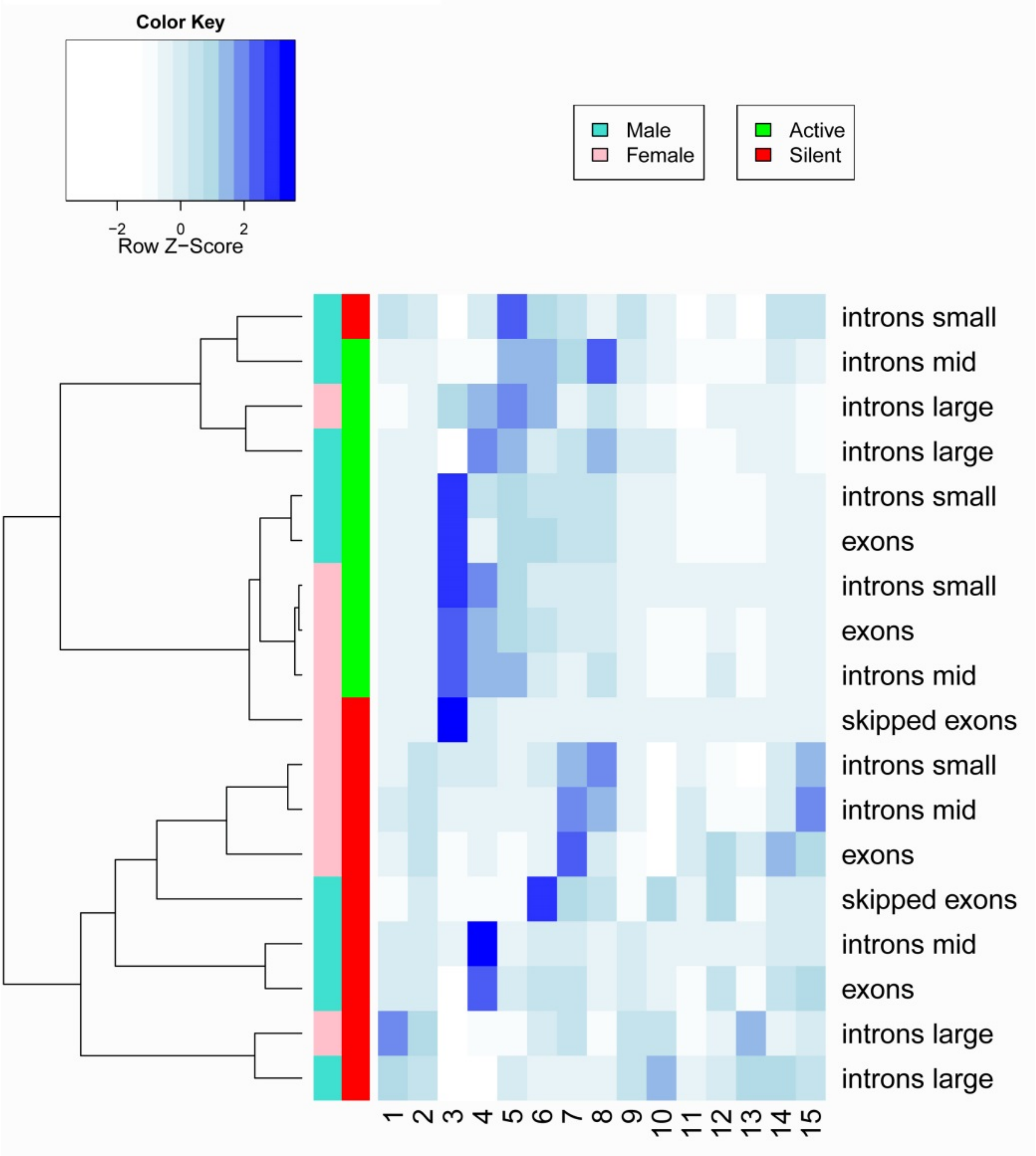

Supplemental Fig. S10. Distinct chromatin states were observed for large introns and skipped exons. Chromatin state fold-enrichment (converted to z-scores to highlight the relative fold-enrichments for each feature) heatmap for expressed (green) and silent (red) introns and exons in the ovary (pink) and testis (light blue). Introns were separated by length into short (< 50 nt), mid (50 - 100 nt) and long (> 100 nt). Features were clustered by correlation of fold-enrichments across chromatin states (darker blue = higher enrichment) of a particular state. Notably, the chromatin states of large introns cluster as a function of their expression level, irrespective of the sample being from the ovary or the testes.

Supplemental Figure S11

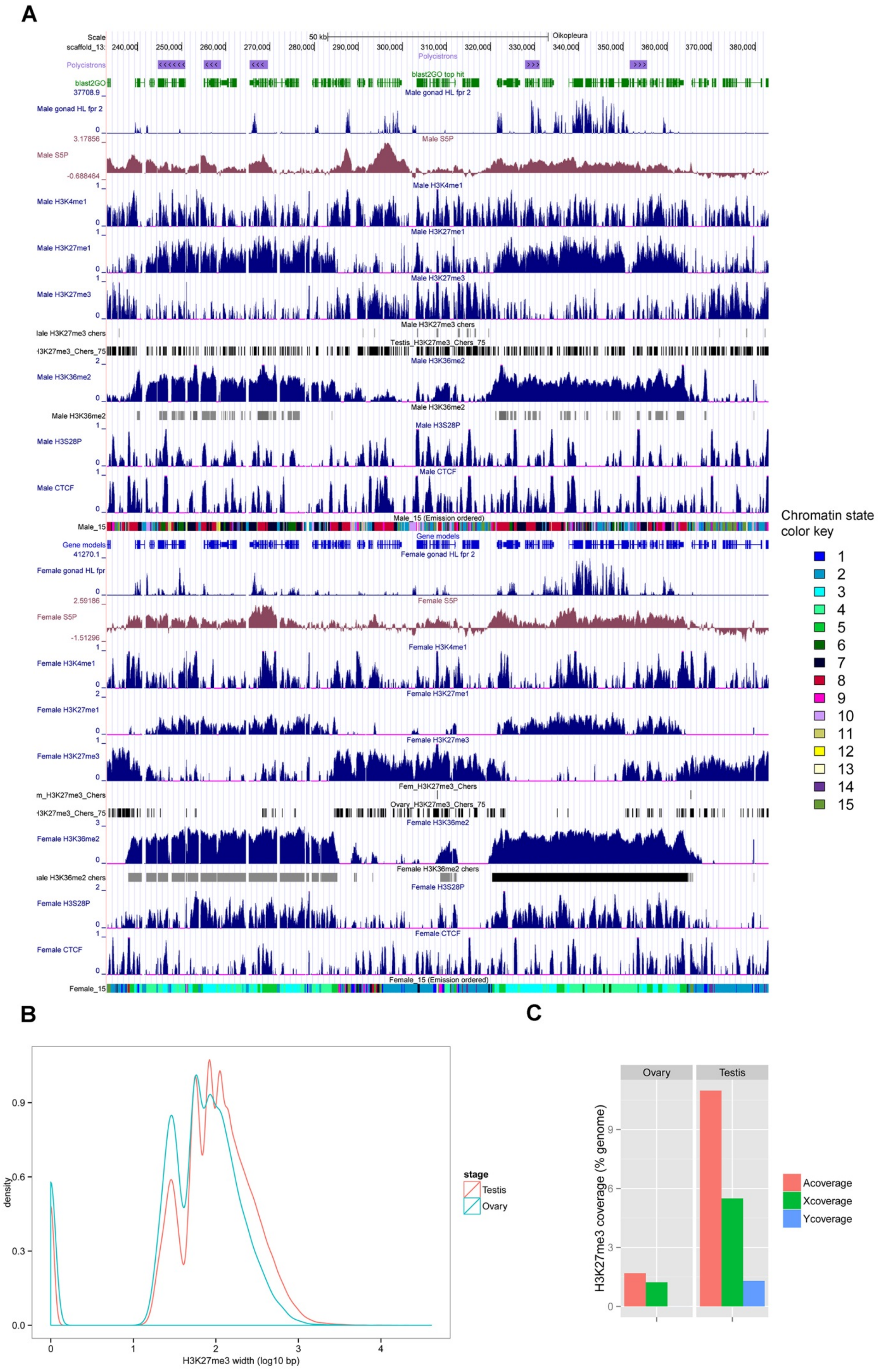

Supplemental Fig. S11. Polycomb domains resolved by using a lower (75%) threshold for cher detection. (A) Genome browser screenshot showing histone PTMs over a 1.5 Mb region. Tracks of Polycomb-related histone PTM signal/input distributions and H3K27me3 chers using 95 (top of paired row) and 75% (bottom of paired row) thresholds were complementary to the H3K36me2 chers (95% threshold). (B) Distribution of cher breadth and (C) Relative coverage of the 75% threshold-detected H3K27me3 domains on autosomal (Acoverage) and X- and Y- chromosomes.

Supplemental Figure S12

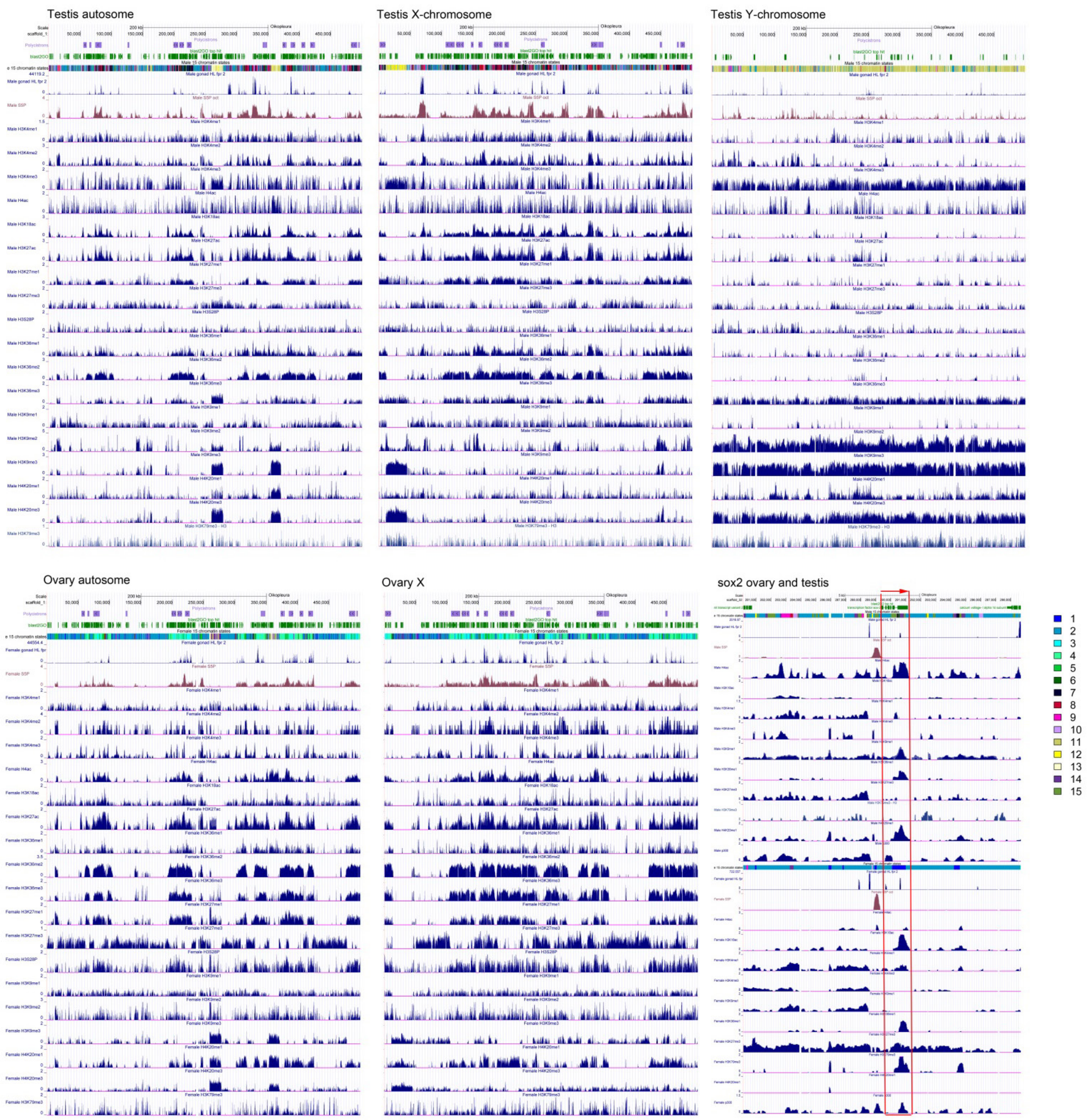

Supplemental Fig. S12. Genome browser screenshots showing histone PTMs over 0.5 Mb regions of sex chromosomes and autosomes together with chromatin state (15-state model) and selected annotation tracks. HL fpr 2 = processed tiling microarray probe intensity signal (expression data). Selected tracks in the sox2 genomic locus show differences in histone PTM distributions and chromatin states between ovary and testis. The sox2 gene with its promoter is boxed in red.

Supplemental Figure S13

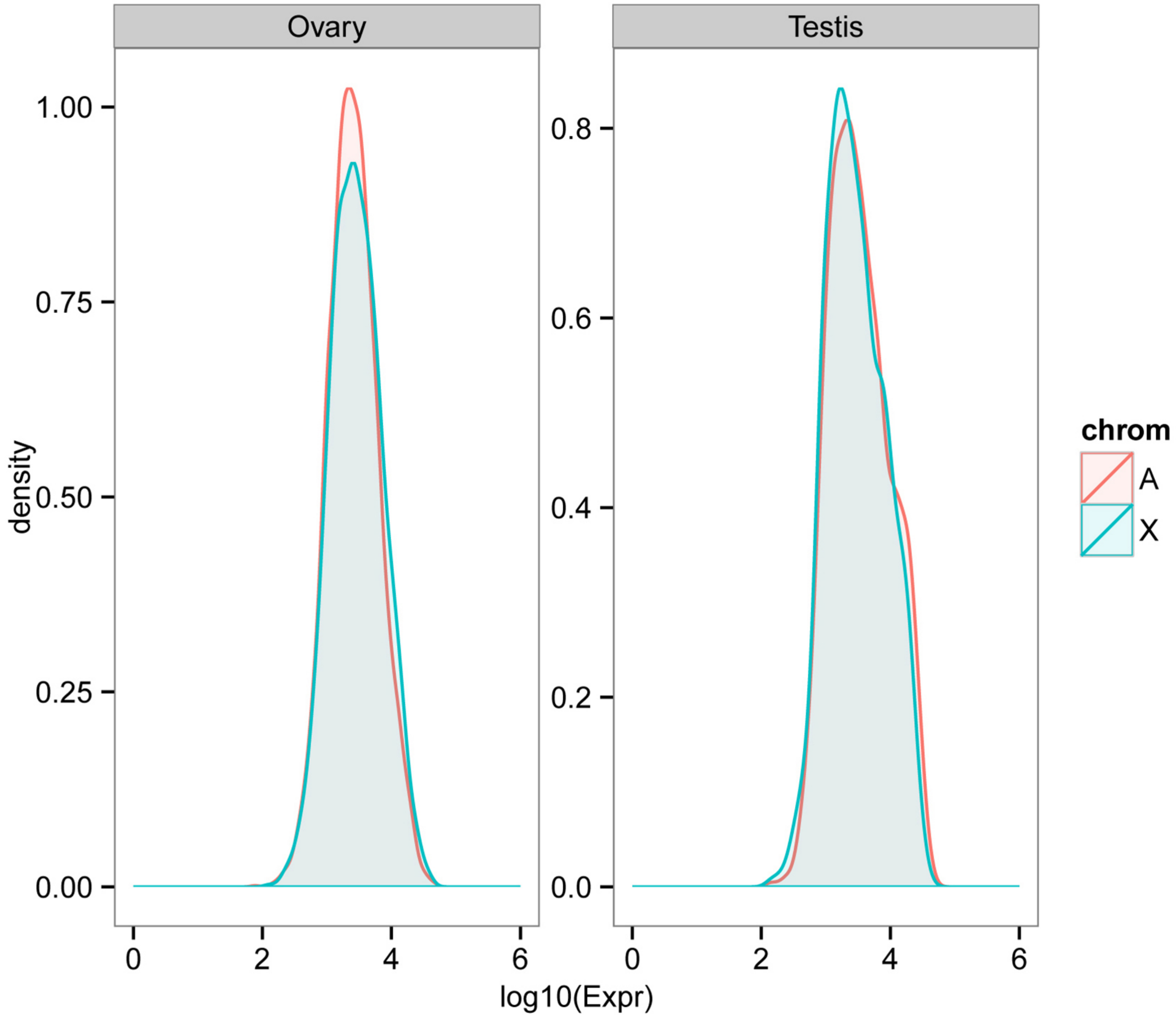

Supplemental Fig. S13. Expression value distributions of autosomal and X-chromosomal genes in *O. dioica* ovary and testis indicate a dosage compensatory mechanism.

References

1. Ernst J, Kheradpour P, Mikkelsen TS, Shoresh N, Ward LD, Epstein CB, et al. Mapping and analysis of chromatin state dynamics in nine human cell types. Nature. 2011;473:43–9.
