# Supplementary material for: Sex-specific chromatin landscapes in an ultra-compact chordate genome
Source: Epigenetics Chromatin. 2017 Jan 17;10:3. doi: 10.1186/s13072-016-0110-4 (PMC5240408; doi:10.1186/s13072-016-0110-4)
Supplement: Supplementary file 3 — Additional file 3. Supplementary Tables S1 to S6 with accompanying footnotes and references. [file 13072_2016_110_MOESM3_ESM.pdf]

ADDITIONAL FILE 3: Supplemental Tables S1-S6

Sex-specific chromatin landscapes in an ultra-compact chordate genome

P. Navratilova, G. B. Danks, A. Long, S. Butcher, J. R. Manak and E. M. Thompson

Supplemental Table S1. Antibodies for histone PTM ChIP-chip profiles.

Histone PTM	Antibody (Catalog #)	Supplier	Replicates (ovary/testis)
H3K18ac	ab1191	Abcam	2/2
H3K27ac	ab4729	Abcam	2/2
H3K27me1	07-448	Millipore	2/2
H3K27me3	ab6002	Abcam	3/4
H3K36me1	ab9048	Abcam	2/2
H3K36me2	ab9049	Abcam	2/2
H3K36me3	ab9050	Abcam	2/2
H3K4me1	ab8895	Abcam	3/3
H3K4me2	ab32356	Abcam	1/1
H3K4me3	04-745	Millipore	2/2
H3K79me1	ab2886	Abcam	2/2
H3K79me3	ab2621	Abcam	1/2
H3K9me1	ab89906	Abcam	3/2
H3K9me2	ab1220	Abcam	1/1
H3K9me3	ab8898	Abcam	3/2
H3S28P	ab10543	Abcam	2/2
H4ac	06-866	Millipore	2/3
H4K20me1	ab9051	Abcam	2/2
H4K20me3	ab9053	Abcam	2/2
RNAP-S5P	ab5131	Abcam	2/2
RNA polII-S2P	ab5095	Abcam	2/2
RNA polII-CTD	ab5408	Abcam	2/2

Supplemental Table S2. *O. dioica* epigenome 15-state model: tissue-specific proportional genome coverage and ChromHMM emission parameters.

State	1	2	3	4	5	6	7	8	9	10	11	12	13	14	15
**Percent of Genome Coverage**															
**ovary**	7.72	53.87	8.63	11.56	4.18	2.27	0.60	0.27	2.65	1.73	0.02	0.09	1.56	2.97	1.89
**testis**	0.21	39.20	0.001	0.004	0.02	2.71	9.46	6.02	3.66	7.05	15.48	3.70	4.49	4.40	3.60
**ChromHMM emission parameters**															
**H4ac**	0.02874	0.00014	0.99622	0.00000	0.85336	0.20385	0.12141	0.10498	0.08668	0.03729	0.05881	0.13679	0.15455	0.00127	0.98501
**H3K27me1**	0.00203	0.00000	0.15003	0.06210	0.32737	0.00856	0.00803	0.03409	0.00130	0.00422	0.00052	0.00162	0.27154	0.01366	0.00544
**H3K9me1**	0.01652	0.00002	0.01506	0.00469	0.08008	0.00317	0.00086	0.00050	0.00569	0.00075	0.00000	0.00041	0.12164	0.01324	0.00424
**H4K20me1**	0.05953	0.00000	0.13040	0.17588	0.43914	0.09901	0.05421	0.12308	0.01543	0.03319	0.10109	0.14777	0.75603	0.14404	0.05316
**H3K79me1**	0.03858	0.00015	0.11940	0.11954	0.44294	0.05513	0.04544	0.15427	0.03476	0.08522	0.09003	0.14291	0.77207	0.19383	0.04052
**H3K4me1**	0.06857	0.00000	0.00824	0.00679	0.13682	0.08830	0.03736	0.24006	0.02849	0.00529	0.00163	0.04197	0.39525	0.05593	0.04816
**H3K4me3**	0.00112	0.00000	0.00004	0.00040	0.46693	0.00592	0.04842	0.69469	0.09454	0.03784	0.27418	0.22559	0.61287	0.07032	0.01463
**H3K27ac**	0.00195	0.00007	0.10141	0.00359	0.75396	0.34768	0.76481	0.91800	0.08383	0.02408	0.05126	0.04389	0.32639	0.00793	0.03026
**H3K18ac**	0.10839	0.00015	0.06958	0.01147	0.23334	0.15921	0.43503	0.84657	0.06474	0.01945	0.00497	0.00382	0.03536	0.00207	0.03774
**H3K36me1**	0.09032	0.00000	0.10821	0.08830	0.23093	0.20907	0.23136	0.49625	0.02650	0.04601	0.01888	0.02128	0.04237	0.10781	0.10679
**H3K4me2**	0.06262	0.00000	0.05230	0.07310	0.79428	0.08187	0.00026	0.92972	0.98011	0.00080	0.05081	0.01317	0.22261	0.00010	0.00070
**H3S28P**	0.00440	0.00000	0.00456	0.00188	0.01976	0.05047	0.01238	0.03706	0.05285	0.01213	0.00593	0.01111	0.08933	0.06477	0.01774
**H3K27me3**	0.00467	0.00000	0.00000	0.00005	0.00356	0.04557	0.02332	0.05004	0.03181	0.00844	0.03519	0.12120	0.48949	0.10091	0.07685
**H4K20me3**	0.17678	0.00000	0.00621	0.00288	0.04232	0.01142	0.00526	0.04921	0.01817	0.01639	0.17589	0.25002	0.82354	0.18118	0.04983
**H3K9me3**	0.00091	0.00011	0.00002	0.00000	0.00037	0.00987	0.01220	0.16998	0.03752	0.00246	0.99858	0.99003	0.77199	0.00196	0.00229
**H3K9me2**	0.35351	0.00000	0.05519	0.10156	0.29271	0.10333	0.11062	0.30140	0.26702	0.97634	0.99922	0.00070	0.89409	0.00350	0.02645
**H3K79me3**	0.86131	0.00023	0.22364	0.18398	0.45619	0.01153	0.01137	0.03320	0.05495	0.00549	0.03915	0.06019	0.69212	0.01659	0.08266
**H3K36me3**	0.41873	0.00041	0.99500	0.98616	0.81073	0.00741	0.00605	0.01500	0.02142	0.00542	0.03557	0.02842	0.43374	0.01104	0.01082
**H3K36me2**	0.07250	0.00008	0.99901	0.99295	0.95716	0.98975	0.00035	0.07708	0.11065	0.00530	0.00168	0.00205	0.14813	0.00014	0.00037

Supplemental Table S3. Characterization of the 15 chromatin state model in *Oikopleura dioica* by summary of the genomic features enriched in each state.

State	Histone PTMs	Characteristic associated Genomic and Chromatin Features	
		Ovary	Testis
1	**H3K79me3,** H3K36me3 H3K9me2, H4K20me3	silent HD, TF and ZF promoters, low specific gene promoters, silent HD gene bodies – **Silent TFs**	silent HD genes and promoters, ZF and TF promoters, silent TF gene bodies, CTCF
2		unannotated, silent regions	unannotated, silent regions
3	**H4ac, H3K36me3, H3K36me2**	active operons, RNApol II S5P, active gene bodies, low-specific genes, non-TF and some TF and ZF genes, active TARs, UTRs, skipped exons – **Transcriptional elongation, alternatively spliced exons**	RNApol II S5P, active operons, bodies of low specificity genes, non-TF genes p300**,** X scaffolds – **Transcriptional elongation**
4	**H3K36me3, H3K36me2**	active HD, ZF and TF genes, RNApol II S5P, UTRs, active operons, active non-TF genes – **Transcriptional elongation esp. operons**	silent intergenic TARs, silent operon promoters and bodies, low-specificity gene bodies, CTCF, p300, promoters and gene bodies of active non-TF genes
5	**H4ac, H3K4me2, H3K27ac, H3K36me2, H3K36me3,** H3K4me3, H3K79me1,3**,** H4K20me1	RNApol II S5P, operon promoters, low-specif and non-TF gene promoters, active ZF and TF promoters, active 3 and 5 UTRs, p300 **- Active promoters esp. operons and low specificity genes**	RNApol II S5P, active low specificity gene bodies, operon gene bodies; silent operon promoters and gene bodies, low specificity gene promoters and bodies; CTCF, p300, X chromosome
6	**H3K36me2,** H3K27ac	active intergenic TARs, active ZF genes – **Intergenic transcription**	skipped exons, active operon and low-specificity gene bodies and non-TFs, silent operons and low-specificity gene bodies, intergenic TARs, 5meC – **Gene bodies, alternatively spliced exons**
7	**H3K27ac,** H3K18ac	silent intergenic TARs, active high specificity ZF and TF promoters; active intergenic TARs, silent TFs, 5meC	active operon and low-specif. gene bodies, active TFs and ZFs, non-TF genes,5UTRs, silent operons and low specificity gene bodies **- Gene bodies**
8	**H3K4me3, H3K27ac, H3K18ac, H3K4me2,** H3K36me1	active high specificity ZF and TF promoters; active low- specificity genes, non-TF gene promoters; active operon promoters, RNApol II S5P, silent non-TF promoters, low and high specificity promoters, silent intergenic TARs	active high specificity ZF and TF promoters; active low- specificity genes, non-TF gene promoters; active operon promoters, RNApol II S5P, active 3 and 5 UTRs, active intergenic TARs **- Active promoters**
9	**H3K4me2,** H3K9me2	active ZFs, TFs and non-TF gene promoters, high-specif promoters. silent operons and ZF promoters **- Active and silent tissue-specific promoters**	active HD genes, CTCF, active high specificity, ZFs and TFs promoter, silent operons and ZFs promoters **- Active and silent promoters**
10	**H3K9me2**	silent intergenic TARs, CTCF, TEs – **Heterochromatin**	no features are enriched over the threshold

11	**H3K9me3, H3K9me2,** H3K4me3	CTCF, silent ZF gene bodies, TEs, p300	Y scaffolds, TEs **- Silent Y-chromosomal regions**
12	**H3K9me3,** H3K4me3, H4K20me3	5meC, CTCF, p300, TEs, silent low specif. genes	**Heterochromatin**
13	**H4K20me1, H3K79me1 H3K4me3, H4K20me3, H3K9me3, H3K9me2, H3K79me3,** H3K4me1**,** H3K27ac, H3K36me3	CTCF, TEs **- Silent regions**	active ZF promoters, p300, TE, CTCF, 5meC, Y scaffolds - **Active Y-chromosomal genes and mobile elements, enhancers**
14	H3K27me3, H4K20me1, H4K20me3 H3K79me1, H3K36me1	active HD genes, 5meC, CTCF, TE	active and silent HD genes – **Polycomb domains**
15	**H4ac**	5meC, p300, CTCF, silent intergenic TARs, active high specif. genes	silent intergenic TARs, silent HD and TF promoters and gene bodies **– Silent developmental genes**

The cutoff emission values for inclusion of histone PTMs in the Table was 0.25, except for state 14 (grey font) where emission values were >0.1, but <0.2. Those PTMs with values >0.5 are in bold. TF = transcription factor; ZF = zinc finger protein; high and low specificity genes according to breadth of expression across development (see Methods); TAR = transcriptionally active region; TE = transposable element; unannotated = regions lacking annotation or transcription; 5meC = methylcytosine; esp.- especially. Grey-shaded boxes indicate that the state appeared in a very small (<1%) proportion of the genome in respective tissue. Red/green font indicates transcriptionally silent/active genomic regions. Putative annotation/summaries of composite features associated to states are indicated in bold font where possible. Thresholds on the emission parameters for the state enrichment of each feature were >2.

Supplemental Table S4. Comparison of the 50-chromatin state model to the 15-state model.

50-state model	Features in the ovary	Feature in the testes	15-state model
1	Operons, non-TF promoters, low specif. promoters, RNA polII, skipped exons		5
2	Operons, non-TF promoters, RNA polII		5
3	Operons, RNA polII		3
4	Operons		3
5	Operons, RNA polII		3
6	Operons, UTRs		4
7	Operons		4
8	Operons, UTRs		4
9	HD promoter		1
10	HD promoter, TF and ZF promoter		1
11	High specif. promoters, ZF, TF, operon promoters	Active and silent gene bodies	2
12	Operon bodies, non-TF and low-specif. gene bodies, skipped exons		3
13	Operon bodies, non-TF and low-specif. gene bodies, ZF and TF gene bodies, RNA polII	low specif., non-TFgene bodies, p300	3
14	CTCF, active HD gene bodies	HD promoter and body	13
15		Y-chromosome, TE	11
16		TE	11
17	CTCF	TE upstream TSS	11
18	CTCF, unannotated, TE	TE downstream TSS, Y-scaffolds	11
19		TE, Y-chromosome	11
20		Intergenic TARs, TF promoter, high specif. gene promoters, CTCF, Y-chromosome	11
21	Operons, HD, TF, ZF promoters	HD, TF promoters, intergenic TARs, TF promoters, intergenic TARs	9
22	Intergenic TARs, HD promoter	Broad range of features	10
23	TE, CTCF, HD promoter	CTCF, HD promoter	13

24	CTCF		13
25	TE, operons and non-TF	Intergenic TARs, UTR3s	13
26		5meC, non-TF and non-SL promoter, Y-chromosome	13
27	5meC, CTCF, TFs, ZFs	HD	13
28	CTCF, TE, p300	3`UTRs, high specif., TF, ZF promoters	13
29		RNA polII, high specific, low specif. and non-TF promoters, ZF and TF promoters, operon promoters, CTCF, p300	8
30	RNA polII, high and low specif. promoters and gene bodies	RNA polII, active specific, low specif. and non-TF promoters, operon promoters, 5 and 3`UTRs, intergenic TARs	
31	RNA polII, UTRs, ZF and TFs promoters, CTCF	RNA polII, active non-TF promoters, operon promoters, 5 and 3`UTRs, gene bodies, intergenic TARs	
32	High specif. promoters and gene bodies, HD, TF and ZFs promoters	High specific. promoters, low specific promoters, operon promoters, operon, non-TF promoters	
33	High specif. gene body	HD gene body	9
34	High specif., TFs, ZFs promoters, 5`UTRs,	CTCF, p300, intergenic TARs,	5
35	intergenic TARs	active and silent low specif. gene, non-TF, operons, intergenic TARs,	6
36	specif. TFs, ZF, operon promoters	low specif. gene, non-TF, operons, CTCF	6
37	active and silent specif. TFs, intergenic TARs	low specif. gene, non-TF, operons,	8
38		ZF, TF, HD, low specif, operon gene bodies	8
39	intergenic TARs, TE	operon, ZF, HD gene bodies, active and silent intergenic TARs	8
40	CTCF, intergenic TARs	5 `UTRs, RNA polII	8
41	ZF, TF gene bodies, HD gene bodies, intergenic TARs	RNA polII, intergenic TARs, high specif. promoters and gene bodies,	7
42	HD promoter, low specif. gene bodies	intergenic TARs, 5 `UTRs, RNA polII, high specif. promoters	7
43	TF, ZF, HD promoters and gene bodies	TF, ZF, HD promoters and gene bodies	1
44	5meC, high specif genes, TFs,	TF, ZF promoters, ZF, TF gene bodies	15
45	RNA polII, TFs	intergenic TARs, HD, ZF gene bodies	15
46	Silent ZF, TF, HD promoters and gene bodies - Polycomb domains	ZF, HD promoters and gene bodies, intergenic TARs, X-chromosome, CTCF - Polycomb domains	13
47	unannotated, broad range	unannotated, broad range	2

48	5meC, CTCF, p300	5meC, CTCF, p300	12
49	Active and silent high specific. promoters and bodies, HD, TFs, intergenic TARs	RNA polII, high specific. promoters	8
50	CTCF, active 5`UTRs	CTCF, p300	12 / 13

Expansion of certain states (15-state model) into several sub-states (50-state model) is indicated by the color code assigned to each of the 15 states. Grey shading in the middle rows indicate states covering <0,01% (< 7 kb total) of the genome. Red type, features associated with transcriptionally silent regions; Green type, features associated with transcriptionally active regions; Black type, no particular association with active or silent regions. TF, transcription factor; ZF, zinc finger genes; HD, homeodomain genes; specific., specificity; TAR, transcriptionally active region

Supplemental Table S5. GO terms associated with chromatin state enrichment clusters based on the *O. dioica* 15-chromatin state epigenome model.

State	Ovary			Testis		
	Clusters	Genomic Features	GO terms	Clusters	Genomic Features	GO terms
**1**	1	silent promoters	Chordate embryonic development, cell fate commitment, calcium ion binding, positive and negative regulation of transcription, organ morphogenesis, positive regulation of metabolic process and gene expression – **Embryonic development**	16	silent genes	M-phase, growth, Wnt signaling, gastrulation, growth, embryonic development, organ development, apoptosis, cell proliferation, microtubule-based process, tissue development, positive and negative regulation of cell proliferation, transcription, ribosome biogenesis, translation, signal transduction, microtubule based process, membrane organization – **Embryonic development**
	31	silent genes	Transcription, cell adhesion, developmental process, muscle differentiation, nervous system development, sex differentiation, tissue development, positive regulation of cell proliferation, cell growth, embryonic morphogenesis, Wnt signaling, tube development, organ morphogenesis – **Embryonic development**			
**3**	16	active gene bodies, TSS and TES	ATP binding, DNA binding, DNA replication, Golgi apparatus, chromatin organization, cell morphogenesis, biosynthesis, glucose, alcohol, amono acid metabolism, embryo development, reproduction, M phase, translation, cell adhesion, ectoderm development, cell proliferation, meiosis, mitochondria – **Maternal transcripts, Oogenesis, Housekeeping genes**	18	active gene bodies<br><br>silent gene bodies	**A:** Cytoskeleton, centrosome, spindle, microtubule-based processes, MTOC, GTPase activity, organelle organization, membrane, cation transport, nucleolus, cell communication, signaling, transport – **Mitosis related, housekeeping functions**<br><br>**S:** Immune system process, intracellular transport, membrane bounded vesicle-mediated transport, clathrin coated vesicle, lipid binding, defense response, response to stress, response to other organism – **Stress response**
	17	active gene bodies<br><br>silent gene bodies	**A:** DNA recombination, repair, ATP metabolism, GTPase activity, reproduction, M phase, RNA splicing, aging, cell adhesion, growth, apoptotic process, signaling, methylation, embryo development ending in hatching, gametogenesis, histone modification, ribosome, - **Maternal transcripts, Oogenesis, Housekeeping genes**<br>**S:** Methylation, ribosome biogenesis, ncRNA metabolism, tRNA metabolism, mRNA processing, regulation of translation - **Nutrient response**			
**4**	16		Overlap with states #3 and 5	19	silent TSS promoters, gene bodies	**S:** DNA damage response, meiosis, M-phase, gamete generation, reproduction, lipid biosynthesis, ribosome, endoplasmic reticulum, protein import, translation, zinc ion binding – **Gametogenesis, Meiosis**<br><br>**A:** RNA biosynthesis, cell-cell signaling, regulation of hormone
	17					

	24				some active gene bodies	levels, alcohol metabolism, mitochondrial matrix, secretion, actin-filament based process
**5**	14<br>16	active TSS	Response to nutrient, locomotory behavior, contractile fiber, ion channel activity, cellular protein complex assembly, regulation of binding, cation transport, microtubule-based movement, fatty acid metabolism – **Nutrient response**	20	active gene bodies<br>silent gene bodies	**A:** DNA replication, packaging, M-phase, chromosome organization, RNA splicing , anatomical structure,cellular metabolism,development, apoptosis, aging, response to hormone, chromatin organization, embryo development, gastrulation, male gamete generation, spermatogenesis<br>**S:** MAPK cascade, ATPase activity, RNA processing, ncRNA processing, ribonucleoprotein complex biogenesis, RNA splicing, reproduction, M-phase, ectoderm development, growth, hormone metabolism, spermatogenesis<br>– **Mitosis, spermatogenesis, development**
	17	active gene bodies	Overlap with states #3 and 4			
	24	active promoters and TSSes	Cell proliferation, developmental process, methylation, regulation of transport, of cell cycle and cellular metabolism, membrane organization, proteolysis, DNA metabolism, tRNA metabolism, translation, vesicle transport **– Housekeeping functions**	21	silent gene bodies	Cell development and differentiation, actin cytoskeleton, RNA metabolic process, nucleoplasm, nucleic acid binding, apoptotic process, programmed cell death, positive and negative induction and regulation of apoptosis, of cellular growth, of proliferation; homeostatic process, hydrolase, phospholipase and carboxylesterase activity - **Apoptosis**
	26	active promoters	Reproduction, M-phase, meiosis, DNA replication, growth, nucleolus, regulation of apoptosis, metabolism, gastrulation, hormone biosynthesis, embryonic morphogenesis, developmental process – **Oogenesis, maternal transcripts**			
**6**	27<br>28	silent gene bodies, TSS and TES	MAPK cascade, transmembrane receptor PTK signaling, hormone response, developmental growth, gastrulation, anatomical structure development, fatty acid metabolism, Immune system process, sex differentiation, metabolic processes – **Embryonic development**	7	active gene bodies and TESes	**A:** ATP metabolism, Golgi membrane, MAPK, Ras signaling, , tRNA metabolism, DNA recombination, repair, vesicle-mediated transport, cell-cell signaling, cell growth, ligase activity, ncRNA processing, spermatogenesis, histone modification, NTP synthesis, GTPase regulator activity<br>- **Housekeeping functions and spermatogenesis**
				17	silent gene bodies	ATP binding, DNA binding, repair, proliferation, M-phase, GTPase, cellular metabolism,RNA metabolism, actin, chromatin organization, protein complex assembly, tissue development, embryonic development, organ development, regulation of cellular growth, larval development,<br>- **Mitosis, metabolism, embryonic development**
**7**	3<br>4	silent promoters, TSS	Cell activation, TF binding, endocytosis, ectoderm development, developmental process, tube development, microtubule-based process, cell-cell signaling, tissue morphogenesis, muscle development, gastrulation - **Larval development**	4<br>6	active TSS	Overlap with states #6 and 8
	42	silent gene	Anatomical structure morphogenesis, cellular component biogenesis, protein complex biogenesis, M phase, mitosis,			

	43<br>44	bodies	growth, DNA packaging, gastrulation, chromatin organization, larval development, cell fate commitment, locomotory behavior, cell adhesion – **Larval development**	17	silent TSS	
**8**	22	active genes	DNA binding, DNA replication, embryo development, Golgi vesicle transport, MAPK cascade, Wnt receptor signaling pathway, cellular homeostasis, chromatin organization, negative and positive regulation of many processes, sex differentiation, cytoskeleton organization, nucleosome assembly, embryo development, organogenesis - **Maternal transcripts, Oogenesis**	4	active promoters	ATPase activity, chromatin assembly, muscle contraction, ncRNA metabolism, cilium, heat shock binding, hormone biosynthesis, lipid localization, reproductive process, microtubule-based process, mitosis and meiosis, DNA packaging, protein localization, cellular biosynthesis process, cellular development, centrosome, growth, heat shock, binding, hormone biosynthesis, male gamete generation, mitosis, meiosis, ncRNA metabolism, response to drug, nutrient, stress, ribosome, spliceosome, tissue development – **Spermatogenesis, nutrient response**
	23	silent promoters	DNA recombinations, M-phase, cell motility, chromatin organization, heat shock protein binding, hormone signaling, spermatogenesis, lipid metabolism, negative and positive regulation of many processes, meiosis, apoptosis - **Spermatogenesis**	6	active TSS TES and gene bodies	
**9**	37	silent promoters	M-phase, cell-cell signaling, locomotion, developmental growth, spermatogenesis, Wnt pathway - **Spermatogenesis**	9	silent promoters, TSS and TES	Spermatogenesis, gene regulation, sex differentiation, reproduction, response to hormone, mitotic cell cycle, ATP, GTP binding, actin, response to chemical, induction of apoptosis, organelle fission, phosphorylation, protein ubiquitination, endocyctosis, Ras signaling, vitamin B6 binding, immune system process – **Spermatogenesis, Stress response**
	29	silent promoters	protein serine/threonine kinase signaling pathway, multicellular organism reproduction, response to steroid hormone, regulation of mitosis, protein phosphorylation - **Spermatogenesis**			
**10**	34<br>35	silent genes	Response to radiation, defense response, embryonic organ development, regulation of translation, response to biotic stimulus, small GTPase-mediated signal transduction. polysaccharide metabolid process – **Stress response, larval development**	12<br>13	silent	ectoderm development, cell adhesion, regulation of molecular function, developmental growth, muscle contraction, response to wounding and radiation – **Larval development, Stress response**
**11**	40	silent	RNA-dependent DNA replication, nitrogen compound metabolism	1	silent	RNA-dependent DNA replication
**12**				1	silent	RNA-dependent DNA replication
**13**	2	silent genes	Nuclear division, response to nutrient, hormone, drug, wound healing, transmembrane transport, regulation of hormones, nucleotide biosynthesis, nucleolus, MAPK cascade, negative regulation of cell proliferation, organelle fission – **Stress, nutrient response**	1	silent	RNA-dependent DNA replication
	6		ATP, NTP metabolic process, DNA metabolic process, immune response, response to nutrient, sulfur compound metabolic process, defense response - **Stress, nutrient response**	2	silent	DNA conformation change, NTP metabolic process, organic acid transport, hormone biosynthesis, positive regulation of differentiation, methylation

**14**	10	silent TSS	RNA-dependent DNA replication, DNA integration, response to wounding, neurotransmitter transport, regulation of Ras	3	silent	Reproduction, sex differentiation, spermatogenesis, larval development, sulfur compound metabolism, response to abiotic stimulus, stress, regulation of mitosis
				15	silent TSS and promoters	Wnt signaling, organ development, ectoderm development, anterior-posterior pattern specification, gastrulation, positive and negative regulation of signaling and gene expression, cell growth, tube morphogenesis, regulation of cell proliferation and apoptosis – **Embryonic development**
**15**	8	silent genes	M-phase, cell adhesion, muscle development,Negative regulation of cellular process,wound healing; Wnt signaling, cell growth, chromatin assembly; muscle cell, eye, gland, heart, epithelium development, polysaccharide metabolism, membrane invagination - **Larval development**	11	silent TSS	Wnt signaling, gastrulation, organ development, tube morphogenesis of an epithelium, cell growth, regulation of transcription, microtubule, regulation of cell size, cell migration – **Larval development, TFs**
	19	silent gene bodies	Cellular component biogenesis, dev. process involved in reproduction, sulfur compound metabolism, vesicle mediated transport, cell growth, protein import, regulation of secretion, regulation of cell size	17	silent gene bodies	overlap with states #6 and 7

The cluster numbers are derived from the dendograms in Fig. 3 in the ovary and testis, respectively. Transcriptionally active/silent regions are in green/red font, TF = transcription factor. Putative annotation of each cluster, where possible, is in bold font.

Supplemental Table S6. *Oikopleura dioica* homologs of human histone modifiers.

S6A. Homologs of human histone modification writers in *O. dioica.*

Histone Modifier	Targets	Expression ratio (log_2) ovary/testis	Functions (assigned in literature)
**Histone acetyltransferases**			
**GCN5**	H3K9ac, H3K14ac	NF	Conserved from yeast to humans in locus-specific transcriptional activation (Wang et al. 1997) as a subunit of SAGA and ATAC coactivator complexes [1]. Also acetylates histone H3 in gamma-H2A.X nucleosomes at sites of DNA damage [2].
**PCAF** (p300/CBP-associated factor)	H3K9ac, H4	NF	Paralog of Gcn5 responsible for nuclear receptor-independent co-activation by H3K9ac. Reviewed in [3].
**ELP3**	H3K9ac, H3K18ac, H4	1.66	HAT subunit of RNAP II elongator complex. Involved in transcription, cell cycle regulation and organizing chromatin during mitotic cycles [4].
**CBP**	H3K27ac, H3K18ac, H4ac	2.10	Global transcriptional activator of nuclear receptor-dependent genes [5]. Functional homolog of p300 [6,7].
**p300**	H3K27ac, H3K18ac, H4ac	2.28	Acetylates all 4 core histones and crotonylates histone 3 and 4 for transcriptional co-activation. Binds at promoters and enhancers [6–9].
**Myst1** (hMOF, KAT8)	H4K16ac	0.38	Homolog of *Drosophila* MOF, part of MSL - dosage compensation complex [10] and NSL complex that regulates housekeeping genes [11]. NSL promotes MLL/SET-dependent H3K4me2-marking of some genes [12]. MOF interacts with MLL1 and they jointly establish and spread an active chromatin structure [13].
**Myst2** (HBO1, KAT7)	H3K14ac	11.95	Evolutionarily conserved interaction with origin recognition complex (ORC). HBO1-mediated histone H4 acetylation plays a role in licensing replication origins and gene regulation [14].
**Myst3 or 4** (hMOZ/KAT6A or MORF/KAT6B)	H3 and H4	10.50	Component of the MOZ/MORF complex [15]. Myst3 opposes BMI1 to control Hox gene transcriptional levels along anterior-posterior axis [16]. Myst4 protein is localized in mammalian gametes [17].
**Tip60a** (KAT5)	H3 and H4	10.37	Recruited by E2F transcription factors leading to promoter acetylation of E2F-responsive genes, hence affecting cell cycle progression [18]. Cell-type specific transcriptional activator/repressor [19].
**Tip60b** (KAT5)		0.83	

**MGEA5** (NCOAT)	H4K8ac, H3K14ac	-0.82	Bifunctional protein *O*-GlcNAcase and HAT [20]. E2F1 negatively regulates MGEA5 in Rb1-dependent manner [21].
**SRC1** (Ncoa1)		NF	Facilitates formation of stable pre-initiation complex and transcriptional activation of steroid receptor responsive genes [22].
**ACTR** (Ncoa3)		NF	Associates with other HATs (CBP/p300/PCAF etc.). Chromatin remodelling in response to hormone signalling [23].
**<u>Histone methyltransferases</u>**			
**SETD1B** (dSet1, *Drosophila*; set-2, *C. elegans*)	H3K4	0.22*	Responsible for bulk of H3K4me3 in *Drosophila*. Performs majority of transcription-coupled H3K4me at active genes, presumably through co-transcriptional recruitment [24]. Deficiencies in the H3K4me3 complex of ASH-2, WDR-5 and SET-2 extend lifespan in *C. elegans* [25].
**MLL1**	H3K4me3	NF	TrxG protein activating Hox genes in a cell-specific manner [26]. Interacts with Ash1 in mammals [27]
**MLL3/4** (trr in *Drosophila,* set-1/12/16 in *C. elegans*)	H3K4me1, H3K4me2	1.64	Transcriptional coactivator, required for enhancer activation, cell-type specific gene expression and cell differentiation. MLL4 deletion decreases enhancer H3K4me1/2, H3K27ac, Mediator and RNAPII levels [28]
**MLL5** (trx in *Drosophila*)	H3K4me1,2	1.19	Inhibition of MLL5 results in G1 and G2/M phase cell cycle arrest. Stimulates E2F1 gene expression via binding to HCF1 and facilitates G1/S phase progression [29].
**Ash1** (lin-59 in *C. elegans*)	H3K36me1,2, H3K4me3, K9, H4K20	NF	Maintains retinoic acid-induced transcription of Hox genes by counteracting Polycomb silencing by methylating H3K36 independently of transcriptional elongation during embryonic development in worm, fly and human [27,30–32].
**SETD1A**	H3K4	NF	Induces miRNAs that target downstream effectors of p53, as well as cell cycle regulators in mammals [33]
**SETDB1** (ESET, eggless in *Drosophila*, met-2 in *C. elegans*)	H3K9me1,2	11.97	Part of HP1alpha-CAF1-SETDB1 complex involved in depositing H3K9me in pericentric heterochromatin during DNA replication [34]. Targeted by factor recruited by KRAB zinc-finger proteins to euchromatin, for HP1-mediated silencing of euchromatic genes [35]. Complex with MBD1 and CAF1-HP1 couple DNA methylation and H3K9me3 to propagate heterochromatin during replication[36]. In *C. elegans*, SPR-5 and MET-2 function cooperatively to reestablish an epigenetic ground state required for the continued immortality of the C. elegans germ line [37].
**SETDB2**	H3K9me3	9.68	Contributes to H3K9me3 in interspersed repetitive elements and centromeric repeats. Role in chromosome condensation and segregation during mitosis [38].
**Suv39H1** (set-25 in *C. elegans*)	H3K9me3	0.70	H3K9me3 creates interaction site for HP1 to promote heterochromatin formation in centromeres[39]. Disruption of Suv39H1 leads to aberrant mitosis. SUV39H1 along with SIRT1 mediates silencing of rDNA loci in response to energy deficiency [40].
**Suv39H2**	H3K9me3	11.98	Testis-specific in mouse, accumulates on sex chromosomes (XY body) which undergo transcriptional silencing during meiotic prophase I, may impart an epigenetic imprint to the male germ line [41].

**EHMT1**<br>GLP1	H3K9me2,<br>H3K27me1	10.35	Methyltransferase that targets euchromatic regions. Part of a complex with Suv39h1, GLP1 and SETDB1[42]. Induces DNA methylation and transcriptional repression [43,44].
**EHMT2**<br>G9a	H3K9me1,<br>H3K9me2,<br>H3K27me1	9.82	Catalyzes mono and dimethylation of H3K9 and H3K27 with high specificity [45]. Part of a large multimeric complex of Suv39h1, GLP1 and SETDB1[42].
**EZH1**<br>Enhancer of zeste homolog 1	H3K27me1,<br>H3K27me2,<br>H3K27me3	11.41	Catalytic subunit of Polycomb repressor complex PRC2/EED-EZH1 involved in maintaining transcriptional repressive state of genes over successive cell generations [46]. Low levels of activity, but efficiently represses transcription, compacts chromatin and is ubiquitously expressed in mouse [47].
**EZH2**<br>(mes-2 in *C. elegans*)	H3K27me1,<br>H3K27me2,<br>H3K27me3,<br>H3K9me	9.82	Catalytic subunit of PRC2/EED-EZH2 complex that may serve as recruiting platform for DNA methyltransferases [48]. Expressed in proliferating tissues. Responsible for large-scale changes, onset of differentiation and loss of plasticity in C. elegans [49].
**SETD2**	H3K36me3	11.09	Interacts with hyper-phosphorylated RNAPII [50]. Influences splicing [51]. Prevents cryptic intragenic transcription initiation by recruitment of an HDAC in yeast and FACT complex recruitment in human [52].
**NSD-like**<br>(mes-4 in *C. elegans*, dMes4 in *Drosophila*)	H3K36me1,2	0.87	Regulates gene expression by stimulating transition of RNAPII from initiation to elongation-competent state [53,54]. H3K36me2 peaks adjacent to promoters in *Drosophila* [55]. Responsible for H3K36me2 in *C. elegans* germ line.
**SETMAR**	H3K36me2	nearly silent	Mediates NHEJ repair of DNA damage (dsb). It forms an open chromatin conformation and is responsible for integration of exogenous DNA. This gene aroused from the fusion of a H3 methylase with a mariner family transposase; exists as a fusion gene only in anthropoid primates, other organisms lack mariner transposase domain [56,57].
**SMYD 1 and 3**	H3K4me	silent	Embryonic histone methyltransferases. *C. elegans* homolog set-30 may establish and maintain H3K4me2 on spermatogenesis genes [58].
**SETD3**	H3K4, H3K36	silent	Embryonic histone methyltransferase. Regulates muscle differentiation [59].
**SETD8**<br>(set-1 in *C. elegans*)	H4K20me1	0.60	H4K20me1 helps load pre-RC on origin of replication and plays a role during initiation of DNA replication [60]. Functions with condensin-like *C. elegans* DCC to repress transcription of X-linked genes [61]. Required for further methylations of H4K20 which antagonize H4 acetylation by Myst1 and pauses RNAP II [62].
**Suv4-20H1**<br>(Suppressor of variegation 4-20 homolog 1)	H4K20me2<br>(of<br>H4K20me1)	11.18	Functions in pericentric and telomeric heterochromatin, to establish constitutive heterochromatin [63].
**Suv4-20H2**	H4K20me3<br>(of<br>H4K20me1)	11.33	Stably associates with pericentric heterochromatin via interaction with HP1, resulting in compaction of heterochromatin. Involved in initial loading or maintenance of cohesion subunits and in rDNA antisense lncRNA-mediated global chromatin compaction and establishment of quiescence [64].
**Dot1L**	H3K79me1-3	1.89	Regulates cell cycle progression, may be a specific mark of Wnt target genes. Accumulates on aging histones as a cell aging timer [65,66].

**PRDM9** (set-17 in *C. elegans*)	H3K4me1-3, H3K36me3	-10.55	Meiotic prophase H3K4 and H3K36 tri-methyltransferase essential for meiotic progression and activation of meiotic prophase genes [67–69]. Contributes to transgenerational inheritance in *C. elegans* [70].
**Histone kinases**			
**Aurora B**	H3S10P, H3S28P	-0.15	H3S28P in early prophase required for proper chromosomal condensation [71].
**MSK1_a**	H3S10P (interphase), H3S28P	2.73	H3S28P that initiates methyl-acetyl switch at H3K27 (methylated by Polycomb repressive complex, phosphorylation causes loss of PRC2 interaction) to de-repress target genes [72]. H3S28P also activates stress response genes [73].
**MSK1_b**		2.27	

S6B. Homologs of human histone erasers in *O. dioica.*

Histone modifier	Targets	Expression ratio (log_2) ovary/testis	Functions (assigned in literature)
**Histone deacetylases**			
**SIRT1** (NAD+ - dependent)	H4K16ac, H3K9ac	2.61	Involved in formation of facultative heterochromatin. Mammalian SIRT1 interacts directly with, recruits and deacetylates K266 in SET domain of SUV39H1, and these activities independently elevate SUV39H1 activity to increase H3K9me3 [74]. SIRT1 along with SUV39H1 mediates silencing of rDNA in response to energy deficiency [75].
**SIRT2/3**	H4K16ac, H3K56ac	10.60	Deacetylates H4K16 during mitosis which is crucial for chromatin condensation [76]. Deacetylates BuBR1 which increases lifespan [77].
**SIRT6**	H3K9ac, H3K56ac	10.01	Loss of SIRT6 causes premature lethality and aging-related degeneration. SIRT6 is also involved in DNA repair [78]. Mediates the cell cycle-dependent deacetylation of H3K56 at telomeres [79].
**SIRT7**	H3K18ac	silent	Binds to promoters to promote transcriptional repression. Target genes (linked to tumor suppression) defined in part by interaction with cancer-associated factor ELK4 [80]. Involved in CDK1-cyclin B dependent repression of rDNA transcription at mitosis onset [81].

**HDAC1/2**	H3K56ac, H4?	2.45	Deacetylation of histone tails results in chromatin compaction and the loss of binding sites for transcriptional machinery components that have bromodomains (e.g., TAFII1). Essential for mouse embryogenesis [82].
**HDAC3**	H3, H4	11.05	Plays role in cell cycle progression (required for H3S10P) and DNA damage control. Along with p300, causes deacetylation and repression of the c-myc promoter which leads to transcriptional repression and inhibits premature escape of cells from G1 phase [83].
**<u>Histone demethylases</u>**			
**PHF8**	H3K9me1,2<br>H4K20me1,<br>H3K27me2	0.47	Involved in G1/S transition in conjunction with E2F1, HCF-1 and SETD1A, in part, by removing repressive H4K20me1 mark from a subset of E2F1-regulated promoters [84]. Recruited to promoters via its PHD domain interaction with H3K4me2/3. Coactivator of RNAP I-mediated rDNA transcription [85].
**KDM1A (LSD1)**<br>(spr5 in *C. elegans*)	H3K4me1,2<br>H3K9me1,2	0.13	Belongs to flavin monoamine oxidase family, conserved from *S. pombe* to human [86]. De-represses androgen-receptor-dependent transcription by removing H3K9me [87]. Regulates transgenerational inheritance in *C. elegans* [58].
**KDM1B (LSD2)**	H3K4me1,2,<br>H3K9me2	10.40	Associates with H3K36me3 on transcribed gene bodies and demethylates H3K4me2. Contributes to intragenic dynamics of H3K4me2/H3K9me2. LSD2 depletion results in increased H3K4me2 and decreased H3K9me2 [88].
**KDM2B/2A**	H3K36me2,<br>(H3K4me3)	11.60	Conserved from yeast to humans [89]. Regulates rRNA genes by demethylation of H3K36me2 at promoters under starvation[90]. As part of the PCGF/PRC1 complex bound to non-methylated DNA recruits PRC2 for gene repression [91]
**KDM3A**	H3K9me1,<br>H3K9me2	11.88	Upregulates mammalian Y chromosome sex-determining gene *Sry* [92]. Hormone-dependent recruitment to androgen-receptor target genes, resulting in H3K9 demethylation and transcriptional activation. In spermatogenesis regulates expression of genes such as PRM1 and TMP1, required for sperm chromatin condensation [93].
**KDM4D**	H3K9me3,<br>H3K9me2,<br>H3K4me2	1.98	Has neither a PHD nor TUDOR domain. Catalyzes demethylation of H3K9me3 at enhancer regions leading to their activation [94]. Catalyzes H3K4me2 to H3K4me1. Participates in DNA repair by NHEJ and HR [95].
**KDM5A/B/C/D**<br>(**JARID1 family**: rbr-2 in *C. elegans*, lid in *Drosophila*)	H3K4me3	11.26	Represses homeotic genes by removing H3K4me3[96]. Acts in coordination with Suv39H1 homolog in yeast in establishment of heterochromatin. In euchromatin, associates with Set1 and Lsd1 to function as their structural link and to ensure H3K4 hypermethylation and H3K9 hypomethylation of active genes [97]. Opposes set-2 in regulating the lifespan extension in *C. elegans* [25]. Involved in progression from G2 to M phase. E2F1 is a downstream target in KDM5B pathway [98].
**KDM6A (UTX)**<br>**KDM6B**	H3K27me	11.15<br>12.38	X-chromosomal genes that escape inactivation. H3K27me3 controls the expression of Hox genes by PRC1 recruitment and in germ cells participates in epigenetic reprogramming though KDM6 not required for demethylation of these regions [99,100]. Regulates lifespan in *C. elegans* [101].

**NO66**	H3K4me3, H3K36me3	11.44	Regulates osteoblast differentiation by demethylating H3K4 and H3K36, to inhibit SP7/OSX-mediated promoter activation [102]. Recruited by PHF19 to transcribed regions together with PRC2 during ESC differentiation [103]. A ribosomal oxygenase in nucleolus important for translation regulation [104].
**KDM7_a**<br>**KDM7_b**	H3K9me2, H3K27me2, H4K20me1	silent<br>silent	Removes dimethyl repressive marks and activates transcription[105]. Demethylation of the promoter and further activation of FGF4 gene by KDM7A regulates neuronal differentiation.
**KDM8_a**<br>**KDM8_b**	H3K36me2, H3K36me2	11.88<br>silent	Required for G2/M progression [106]. Specifically demethylates H3K36me2 repressive mark, acting as a transcription activator. Regulates Cdkn1a expression and cell proliferation in embryonic development [107].
**<u>Histone Phosphatases</u>**			
**PP1-alpha catalytic subunit_a**<br>**PP1-alpha catalytic subunit_b**	H3S10P, H3S28P, H3T3P	-12.16<br>0.06	PP1α-mediated dephosphorylation of H3S10P allows deacetylation of H4K5ac/K8ac by HDAC1/2/3, leading to release of chromatin-bound BRD4, and subsequent recruitment of P-TEFb to enhance elongation and expression of inducible genes [108]. LAB-1 may act at the onset of meiosis in a manner akin to Shugoshin, by recruiting PP1 phosphatase to counteract Aurora B kinase, thereby ensuring sister chromatid cohesion [109].

Homologs were identified using human protein sequences from Uniprot and protein BLAST against the *O. dioica* proteome: “NF” indicates that no homolog was found in the *O. dioica* genome using this approach. Homology was validated using InParanoid [110] and/or comparison of functional domain compositions. HAT, histone acetyltransferase; RNAP II, RNA polymerase II; HDAC, histone deacetylase complex; *SET domain spliced out of *O. dioica* SETD1B testis transcript.

References to Supplemental Table 6:

1. Riss A, Scheer E, Joint M, Trowitzsch S, Berger I, Tora L. Subunits of ADA-Two-A-Containing (ATAC) or Spt-Ada-Gcn5-Acetyltrasferase (SAGA) Coactivator Complexes Enhance the Acetyltransferase Activity of GCN5. J. Biol. Chem. 2015;290:28997-9009290:28997-9009.
2. Lee H-S, Park J-H, Kim S-J, Kwon S-J, Kwon J. A cooperative activation loop among SWI/SNF, gamma-H2AX and H3 acetylation for DNA double-strand break repair. EMBO J. 2010;29:1434–45.
3. Nagy Z, Tora L. Distinct GCN5/PCAF-containing complexes function as co-activators and are involved in transcription factor and global histone acetylation. Oncogene. 2007;26:5341–57.
4. Wittschieben BØ, Otero G, de Bizemont T, Fellows J, Erdjument-Bromage H, Ohba R, et al. A Novel Histone Acetyltransferase Is an Integral Subunit of Elongating RNA Polymerase II Holoenzyme. Mol. Cell. 1999;4:123–8.
5. Jin Q, Yu L-R, Wang L, Zhang Z, Kasper LH, Lee J-E, et al. Distinct roles of GCN5/PCAF-mediated H3K9ac and CBP/p300-mediated H3K18/27ac in nuclear receptor transactivation. EMBO J. 2011;30:249–62.

6. Ogryzko V V, Schiltz RL, Russanova V, Howard BH, Nakatani Y. The Transcriptional Coactivators p300 and CBP Are Histone Acetyltransferases. Cell. 1996;87:953–9.

7. Arany Z, Sellers WR, Livingston DM, Eckner R. E1A-associated p300 and CREB-associated CBP belong to a conserved family of coactivators. Cell. 1994;77:799–800.

8. Sabari BR, Tang Z, Huang H, Yong-Gonzalez V, Molina H, Kong HE, et al. Intracellular Crotonyl-CoA Stimulates Transcription through p300-Catalyzed Histone Crotonylation. Mol. Cell. 2015;58:203–15.

9. Rada-Iglesias A, Bajpai R, Swigut T, Brugmann S a, Flynn R a, Wysocka J. A unique chromatin signature uncovers early developmental enhancers in humans. Nature. 2011;470:279–83.

10. Hilfiker A, Hilfiker-Kleiner D, Pannuti A, Lucchesi JC. mof, a putative acetyl transferase gene related to the Tip60 and MOZ human genes and to the SAS genes of yeast, is required for dosage compensation in Drosophila. EMBO J. 1997;16:2054–60.

11. Lam KC, Mühlpfordt F, Vaquerizas JM, Raja SJ, Holz H, Luscombe NM, et al. The NSL complex regulates housekeeping genes in Drosophila. PLoS Genet. 2012;8:e1002736.

12. Zhao X, Su J, Wang F, Liu D, Ding J, Yang Y, et al. Crosstalk between NSL histone acetyltransferase and MLL/SET complexes: NSL complex functions in promoting histone H3K4 di-methylation activity by MLL/SET complexes. PLoS Genet. 2013;9:e1003940.

13. Dou Y, Milne TA, Tackett AJ, Smith ER, Fukuda A, Wysocka J, et al. Physical association and coordinate function of the H3 K4 methyltransferase MLL1 and the H4 K16 acetyltransferase MOF. Cell. 2005;121:873–85.

14. Iizuka M, Stillman B. Histone Acetyltransferase HBO1 Interacts with the ORC1 Subunit of the Human Initiator Protein. J. Biol. Chem. 1999;274:23027–34.

15. Ullah M, Pelletier N, Xiao L, Zhao SP, Wang K, Degerny C, et al. Molecular architecture of quartet MOZ/MORF histone acetyltransferase complexes. Mol. Cell. Biol. 2008;28:6828–43.

16. Sheikh BN, Downer NL, Phipson B, Vanyai HK, Kueh AJ, McCarthy DJ, et al. MOZ and BMI1 play opposing roles during Hox gene activation in ES cells and in body segment identity specification in vivo. Proc. Natl. Acad. Sci. U. S. A. 2015;112:5437–42.

17. McGraw S, Morin G, Vigneault C, Leclerc P, Sirard M-A. Investigation of MYST4 histone acetyltransferase and its involvement in mammalian gametogenesis. BMC Dev. Biol. 2007;7:123.

18. Taubert S, Gorrini C, Frank SR, Parisi T, Fuchs M, Chan H-M, et al. E2F-Dependent Histone Acetylation and Recruitment of the Tip60 Acetyltransferase Complex to Chromatin in Late G1. Mol. Cell. Biol. 2004;24:4546–56.

19. Hlubek F, Löhberg C, Meiler J, Jung a, Kirchner T, Brabletz T. Tip60 is a cell-type-specific transcriptional regulator. J. Biochem. 2001;129:635–41.

20. Toleman C a, Paterson AJ, Kudlow JE. The histone acetyltransferase NCOAT contains a zinc finger-like motif involved in substrate recognition. J. Biol. Chem. 2006;281:3918–25.

21. Muthusamy S, Hong KU, Dassanayaka S, Hamid T, Jones SP. E2F1 Transcription Factor Regulates O-linked N-acetylglucosamine (O-GlcNAc) Transferase and O-GlcNAcase Expression. J. Biol. Chem. 2015;290:31013–24.

22. Spencer TE, Jenster G, Burcin MM, Allis CD, Zhou J, Mizzen CA, et al. Steroid receptor coactivator-1 is a histone acetyltransferase. Nature. 1997;389:194–8.

23. Chen H, Lin RJ, Schiltz RL, Chakravarti D, Nash A, Nagy L, et al. Nuclear Receptor Coactivator ACTR Is a Novel Histone Acetyltransferase and Forms a Multimeric Activation Complex with P/CAF and CBP/p300. Cell. 1997;90:569–80.

24. Lee J-H, Tate CM, You J-S, Skalnik DG. Identification and characterization of the human Set1B histone H3-Lys4 methyltransferase complex. J. Biol. Chem. 2007;282:13419–28.

25. Greer EL, Maures TJ, Hauswirth AG, Green EM, Leeman DS, Maro GS, et al. Members of the H3K4 trimethylation complex regulate lifespan in a germline-dependent manner in C. elegans. Nature. 2010;466:383–7.

26. Guenther MG, Jenner RG, Chevalier B, Nakamura T, Croce CM, Canaani E, et al. Global and Hox-specific roles for the MLL1 methyltransferase. Proc. Natl. Acad. Sci. U. S. A. 2005;102:8603–8.

27. Gregory GD, Vakoc CR, Rozovskaia T, Zheng X, Patel S, Nakamura T, et al. Mammalian ASH1L is a histone methyltransferase that occupies the transcribed region of active genes. Mol. Cell. Biol. 2007;27:8466–79.

28. Lee J-E, Wang C, Xu S, Cho Y-W, Wang L, Feng X, et al. H3K4 mono- and di-methyltransferase MLL4 is required for enhancer activation during cell differentiation. Elife. 2013;2:e01503.

29. Zhou P, Wang Z, Yuan X, Zhou C, Liu L, Wan X, et al. Mixed lineage leukemia 5 (MLL5) protein regulates cell cycle progression and E2F1-responsive gene expression via association with host cell factor-1 (HCF-1). J. Biol. Chem. 2013;288:17532–43.

30. Miyazaki H, Higashimoto K, Yada Y, Endo TA, Sharif J, Komori T, et al. Ash1l Methylates Lys36 of Histone H3 Independently of Transcriptional Elongation to Counteract Polycomb Silencing. PLoS Genet. 2013;9:e1003897.

31. Beisel C, Imhof A, Greene J, Kremmer E, Sauer F. Histone methylation by the Drosophila epigenetic transcriptional regulator Ash1. Nature. 2002;419:857–62.

32. Chamberlin HM, Thomas JH. The bromodomain protein LIN-49 and trithorax-related protein LIN-59 affect development and gene expression in Caenorhabditis elegans. Development. 2000;127:713–23.

33. Tajima K, Yae T, Javaid S, Tam O, Comaills V, Morris R, et al. SETD1A modulates cell cycle progression through a miRNA network that regulates p53 target genes. Nat. Commun. 2015;6:8257.

34. Loyola A, Tagami H, Bonaldi T, Roche D, Quivy JP, Imhof A, et al. The HP1alpha-CAF1-SetDB1-containing complex provides H3K9me1 for Suv39-mediated K9me3 in pericentric heterochromatin. EMBO Rep. 2009;10:769–75.

35. Schultz DC, Ayyanathan K, Negorev D, Maul GG, Rauscher FJ. SETDB1: a novel KAP-1-associated histone H3, lysine 9-specific methyltransferase that contributes to HP1-mediated silencing of euchromatic genes by KRAB zinc-finger proteins. Genes Dev. 2002;16:919–32.

36. Sarraf SA, Stancheva I. Methyl-CpG binding protein MBD1 couples histone H3 methylation at lysine 9 by SETDB1 to DNA replication and chromatin assembly. Mol. Cell. 2004;15:595–605.

37. Kerr SC, Ruppersburg CC, Francis JW, Katz DJ. SPR-5 and MET-2 function cooperatively to reestablish an epigenetic ground state during passage through the germ line. Proc. Natl. Acad. Sci. U. S. A. 2014;111:9509–14.

38. Falandry C, Fourel G, Galy V, Ristriani T, Horard B, Bensimon E, et al. CLLD8/KMT1F Is a Lysine Methyltransferase That Is Important for Chromosome Segregation. J. Biol. Chem. 2010;285:20234–41.

39. Lachner M, O'Carroll D, Rea S, Mechtler K, Jenuwein T. Methylation of histone H3 lysine 9 creates a binding site for HP1 proteins. Nature. 2001;410:116–20.

40. Murayama A, Ohmori K, Fujimura A, Minami H, Yasuzawa-Tanaka K, Kuroda T, et al. Epigenetic control of rDNA loci in response to intracellular energy status. Cell. 2008;133:627–39.

41. O'Carroll D, Scherthan H, Peters AHFM, Opravil S, Haynes AR, Laible G, et al. Isolation and Characterization of Suv39h2, a Second Histone H3 Methyltransferase Gene That Displays Testis-Specific Expression. Mol. Cell. Biol. 2000;20:9423–33.

42. Fritsch L, Robin P, Mathieu JRR, Souidi M, Hinaux H, Rougeulle C, et al. A subset of the histone H3 lysine 9 methyltransferases Suv39h1, G9a, GLP, and SETDB1 participate in a multimeric complex. Mol. Cell. 2010;37:46–56.

43. Tachibana M, Ueda J, Fukuda M, Takeda N, Ohta T, Iwanari H, et al. Histone methyltransferases G9a and GLP form heteromeric complexes and are both crucial for methylation of euchromatin at H3-K9. Genes Dev. 2005;19:815–26.

44. Tachibana M, Matsumura Y, Fukuda M, Kimura H, Shinkai Y. G9a/GLP complexes independently mediate H3K9 and DNA methylation to silence transcription. EMBO J. 2008;27:2681–90.

45. Tachibana M, Sugimoto K, Fukushima T, Shinkai Y. Set domain-containing protein, G9a, is a novel lysine-preferring mammalian histone methyltransferase with hyperactivity and specific selectivity to lysines 9 and 27 of histone H3. J. Biol. Chem. 2001;276:25309–17.

46. Laible G. Mammalian homologues of the Polycomb-group gene Enhancer of zeste mediate gene silencing in Drosophila heterochromatin and at S.cerevisiae telomeres. EMBO J. 1997;16:3219–32.

47. Margueron R, Reinberg D. Chromatin structure and the inheritance of epigenetic information. Nat. Rev. Genet. 2010;11:285–96.

48. Viré E, Brenner C, Deplus R, Blanchon L, Fraga M, Didelot C, et al. The Polycomb group protein EZH2 directly controls DNA methylation. Nature. 2006;439:871–4.

49. Yuzyuk T, Fakhouri THI, Kiefer J, Mango SE. The polycomb complex protein mes-2/E(z) promotes the transition from developmental plasticity to differentiation in C. elegans embryos. Dev. Cell. 2009;16:699–710.

50. Sun X-J, Wei J, Wu X-Y, Hu M, Wang L, Wang H-H, et al. Identification and characterization of a novel human histone H3 lysine 36-specific methyltransferase. J. Biol. Chem. 2005;280:35261–71.

51. de Almeida SF, Carmo-Fonseca M. Design principles of interconnections between chromatin and pre-mRNA splicing. Trends Biochem. Sci. 2012;37:248–53.

52. Carvalho S, Raposo AC, Martins FB, Grosso AR, Sridhara SC, Rino J, et al. Histone methyltransferase SETD2 coordinates FACT recruitment with nucleosome dynamics during transcription. Nucleic Acids Res. 2013;41:2881–93.

53. Li B, Jackson J, Simon MD, Fleharty B, Gogol M, Seidel C, et al. Histone H3 lysine 36 dimethylation (H3K36me2) is sufficient to recruit the Rpd3s histone deacetylase complex and to repress spurious transcription. J. Biol. Chem. 2009;284:7970–6.

54. Lucio-Eterovic AK, Singh MM, Gardner JE, Veerappan CS, Rice JC, Carpenter PB. Role for the nuclear receptor-binding SET domain protein 1 (NSD1) methyltransferase in coordinating lysine 36 methylation at histone 3 with RNA polymerase II function. Proc. Natl. Acad. Sci. U. S. A. 2010;107:16952–7.

55. Bell O, Wirbelauer C, Hild M, Scharf AND, Schwaiger M, MacAlpine DM, et al. Localized H3K36 methylation states define histone H4K16 acetylation

during transcriptional elongation in Drosophila. EMBO J. 2007;26:4974–84.

56. Fnu S, Williamson EA, De Haro LP, Brenneman M, Wray J, Shaheen M, et al. Methylation of histone H3 lysine 36 enhances DNA repair by nonhomologous end-joining. Proc. Natl. Acad. Sci. U. S. A. 2011;108:540–5.

57. Lee S-H, Oshige M, Durant ST, Rasila KK, Williamson EA, Ramsey H, et al. The SET domain protein Metnase mediates foreign DNA integration and links integration to nonhomologous end-joining repair. Proc. Natl. Acad. Sci. U. S. A. 2005;102:18075–80.

58. Greer EL, Beese-Sims SE, Brookes E, Spadafora R, Zhu Y, Rothbart SB, et al. A histone methylation network regulates transgenerational epigenetic memory in C. elegans. Cell Rep. 2014;7:113–26.

59. Eom GH, Kim K-B, Kim JH, Kim J-Y, Kim J-R, Kee HJ, et al. Histone methyltransferase SETD3 regulates muscle differentiation. J. Biol. Chem. 2011;286:34733–42.

60. Beck DB, Burton A, Oda H, Ziegler-Birling C, Torres-Padilla M-E, Reinberg D. The role of PR-Set7 in replication licensing depends on Suv4-20h. Genes Dev. 2012;26:2580–9.

61. Vielle A, Lang J, Dong Y, Ercan S, Kotwaliwale C, Rechtsteiner A, et al. H4K20me1 contributes to downregulation of X-linked genes for C. elegans dosage compensation. PLoS Genet. 2012;8:e1002933.

62. Kapoor-Vazirani P, Kagey JD, Vertino PM. SUV420H2-mediated H4K20 trimethylation enforces RNA polymerase II promoter-proximal pausing by blocking hMOF-dependent H4K16 acetylation. Mol. Cell. Biol. 2011;31:1594–609.

63. Evertts AG, Manning AL, Wang X, Dyson NJ, Garcia BA, Coller HA. H4K20 methylation regulates quiescence and chromatin compaction. Mol. Biol. Cell. 2013;24:3025–37.

64. Bierhoff H, Dammert MA, Brocks D, Dambacher S, Schotta G, Grummt I. Quiescence-Induced LncRNAs Trigger H4K20 Trimethylation and Transcriptional Silencing. Mol. Cell. 2014;54:675–82.

65. Jones B, Su H, Bhat A, Lei H, Bajko J, Hevi S, et al. The histone H3K79 methyltransferase Dot1L is essential for mammalian development and heterochromatin structure. PLoS Genet. 2008;4:e1000190.

66. De Vos D, Frederiks F, Terweij M, van Welsem T, Verzijlbergen KF, Iachina E, et al. Progressive methylation of ageing histones by Dot1 functions as a timer. EMBO Rep. 2011;12:956–62.

67. Hayashi K, Yoshida K, Matsui Y. A histone H3 methyltransferase controls epigenetic events required for meiotic prophase. Nature. 2005;438:374–8.

68. Wu H, Mathioudakis N, Diagouraga B, Dong A, Dombrovski L, Baudat F, et al. Molecular basis for the regulation of the H3K4 methyltransferase activity of PRDM9. Cell Rep. 2013;5:13–20.

69. Powers NR, Parvanov ED, Baker CL, Walker M, Petkov PM, Paigen K. The Meiotic Recombination Activator PRDM9 Trimethylates Both H3K36 and H3K4 at Recombination Hotspots In Vivo. PLoS Genet. 2016;12:e1006146.

70. Greer EL, Beese-Sims SE, Brookes E, Spadafora R, Zhu Y, Rothbart SB, et al. A histone methylation network regulates transgenerational epigenetic memory in C. elegans. Cell Rep. 2014;7:113–26.

71. Goto H, Yasui Y, Nigg EA, Inagaki M. Aurora-B phosphorylates Histone H3 at serine28 with regard to the mitotic chromosome condensation. Genes to Cells. 2002;7:11–7.

72. Lau PNI, Cheung P. Histone code pathway involving H3 S28 phosphorylation and K27 acetylation activates transcription and antagonizes polycomb silencing. Proc. Natl. Acad. Sci. U. S. A. 2011;108:2801–6.

73. Sawicka A, Hartl D, Goiser M, Pusch O, Stocsits RR, Tamir IM, et al. H3S28 phosphorylation is a hallmark of the transcriptional response to cellular stress. Genome Res. 2014;24:1808–20.

74. Vaquero A, Scher M, Erdjument-Bromage H, Tempst P, Serrano L, Reinberg D. SIRT1 regulates the histone methyl-transferase SUV39H1 during heterochromatin formation. Nature. 2007;450:440–4.

75. Murayama A, Ohmori K, Fujimura A, Minami H, Yasuzawa-Tanaka K, Kuroda T, et al. Epigenetic control of rDNA loci in response to intracellular energy status. Cell. 2008;133:627–39.

76. Vaquero A, Scher MB, Lee DH, Sutton A, Cheng H-L, Alt FW, et al. SirT2 is a histone deacetylase with preference for histone H4 Lys 16 during mitosis. Genes Dev. 2006;20:1256–61.

77. North BJ, Rosenberg MA, Jeganathan KB, Hafner A V, Michan S, Dai J, et al. SIRT2 induces the checkpoint kinase BubR1 to increase lifespan. EMBO J. 2014;33:1438–53.

78. Kugel S, Mostoslavsky R. Chromatin and beyond: the multitasking roles for SIRT6. Trends Biochem. Sci. 2014;39:72–81.

79. Michishita E, McCord RA, Boxer LD, Barber MF, Hong T, Gozani O, et al. Cell cycle-dependent deacetylation of telomeric histone H3 lysine K56 by human SIRT6. Cell Cycle. 2014;8:2664–6.

80. Barber MF, Michishita-Kioi E, Xi Y, Tasselli L, Kioi M, Moqtaderi Z, et al. SIRT7 links H3K18 deacetylation to maintenance of oncogenic transformation. Nature. 2012;487:114–8.

81. Grob A, Roussel P, Wright JE, McStay B, Hernandez-Verdun D, Sirri V. Involvement of SIRT7 in resumption of rDNA transcription at the exit from mitosis. J. Cell Sci. 2009;122:489–98.

82. Dovey OM, Foster CT, Cowley SM. Histone deacetylase 1 (HDAC1), but not HDAC2, controls embryonic stem cell differentiation. Proc. Natl. Acad. Sci. U. S. A. 2010;107:8242–7.

83. Sankar N, Baluchamy S, Kadeppagari R-K, Singhal G, Weitzman S, Thimmapaya B. p300 provides a corepressor function by cooperating with YY1 and HDAC3 to repress c-Myc. Oncogene. 2008;27:5717–28.

84. Liu WWT, Tanasa B, Tyurina O V, Zhou TY, Gassmann R, Ohgi KA, et al. PHF8 mediates histone H4 lysine 20 demethylation events involved in cell cycle progression. Nature. 2010;466:508–12.

85. Feng W, Yonezawa M, Ye J, Jenuwein T, Grummt I. PHF8 activates transcription of rRNA genes through H3K4me3 binding and H3K9me1/2 demethylation. Nat. Struct. Mol. Biol. 2010;17:445–50.

86. Shi Y, Lan F, Matson C, Mulligan P, Whetstine JR, Cole PA, et al. Histone demethylation mediated by the nuclear amine oxidase homolog LSD1. Cell. 2004;119:941–53.

87. Metzger E, Wissmann M, Yin N, Müller JM, Schneider R, Peters AHFM, et al. LSD1 demethylates repressive histone marks to promote androgen-receptor-dependent transcription. Nature. 2005;437:436–9.

88. Fang R, Barbera AJ, Xu Y, Rutenberg M, Leonor T, Bi Q, et al. Human LSD2/KDM1b/AOF1 regulates gene transcription by modulating intragenic

H3K4me2 methylation. Mol. Cell. 2010;39:222–33.

89. Tsukada Y, Fang J, Erdjument-Bromage H, Warren ME, Borchers CH, Tempst P, et al. Histone demethylation by a family of JmjC domain-containing proteins. Nature. 2006;439:811–6.

90. Tanaka Y, Okamoto K, Teye K, Umata T, Yamagiwa N, Suto Y, et al. JmjC enzyme KDM2A is a regulator of rRNA transcription in response to starvation. EMBO J. 2010;29:1510–22.

91. Blackledge NP, Farcas AM, Kondo T, King HW, McGouran JF, Hanssen LLP, et al. Variant PRC1 complex-dependent H2A ubiquitylation drives PRC2 recruitment and polycomb domain formation. Cell. 2014;157:1445–59.

92. Kuroki S, Matoba S, Akiyoshi M, Matsumura Y, Miyachi H, Mise N, et al. Epigenetic regulation of mouse sex determination by the histone demethylase Jmjd1a. Science. 2013;341:1106–9.

93. Yamane K, Toumazou C, Tsukada Y, Erdjument-Bromage H, Tempst P, Wong J, et al. JHDM2A, a JmjC-containing H3K9 demethylase, facilitates transcription activation by androgen receptor. Cell. 2006;125:483–95.

94. Zhu Y, van Essen D, Saccani S. Cell-type-specific control of enhancer activity by H3K9 trimethylation. Mol. Cell. 2012;46:408–23.

95. Khoury-Haddad H, Guttmann-Raviv N, Ipenberg I, Huggins D, Jeyasekharan AD, Ayoub N. PARP1-dependent recruitment of KDM4D histone demethylase to DNA damage sites promotes double-strand break repair. Proc. Natl. Acad. Sci. U. S. A. 2014;111:E728-37.

96. Christensen J, Agger K, Cloos PAC, Pasini D, Rose S, Sennels L, et al. RBP2 belongs to a family of demethylases, specific for tri-and dimethylated lysine 4 on histone 3. Cell. 2007;128:1063–76.

97. Li F, Huarte M, Zaratiegui M, Vaughn MW, Shi Y, Martienssen R, et al. Lid2 is required for coordinating H3K4 and H3K9 methylation of heterochromatin and euchromatin. Cell. 2008;135:272–83.

98. Rizwani W, Schaal C, Kunigal S, Coppola D, Chellappan S. Mammalian lysine histone demethylase KDM2A regulates E2F1-mediated gene transcription in breast cancer cells. PLoS One. 2014;9:e100888.

99. Mansour AA, Gafni O, Weinberger L, Zviran A, Ayyash M, Rais Y, et al. The H3K27 demethylase Utx regulates somatic and germ cell epigenetic reprogramming. Nature. 2012;488:409–13.

100. Shpargel KB, Starmer J, Yee D, Pohlers M, Magnuson T. KDM6 demethylase independent loss of histone H3 lysine 27 trimethylation during early embryonic development. PLoS Genet. 2014;10:e1004507.

101. Jin C, Li J, Green CD, Yu X, Tang X, Han D, et al. Histone demethylase UTX-1 regulates C. elegans life span by targeting the insulin/IGF-1 signaling pathway. Cell Metab. 2011;14:161–72.

102. Sinha KM, Yasuda H, Coombes MM, Dent SYR, de Crombrugghe B. Regulation of the osteoblast-specific transcription factor Osterix by NO66, a Jumonji family histone demethylase. EMBO J. 2010;29:68–79.

103. Brien GL, Gambero G, O'Connell DJ, Jerman E, Turner SA, Egan CM, et al. Polycomb PHF19 binds H3K36me3 and recruits PRC2 and demethylase NO66 to embryonic stem cell genes during differentiation. Nat. Struct. Mol. Biol. 2012;19:1273–81.

104. Chowdhury R, Sekirnik R, Brissett NC, Krojer T, Ho C-H, Ng SS, et al. Ribosomal oxygenases are structurally conserved from prokaryotes to humans. Nature. 2014;510:422–6.

105. Yokoyama A, Okuno Y, Chikanishi T, Hashiba W, Sekine H, Fujiki R, et al. KIAA1718 is a histone demethylase that erases repressive histone methyl marks. Genes Cells. 2010;15:867–73.

106. Hsia DA, Tepper CG, Pochampalli MR, Hsia EYC, Izumiya C, Huerta SB, et al. KDM8, a H3K36me2 histone demethylase that acts in the cyclin A1 coding region to regulate cancer cell proliferation. Proc. Natl. Acad. Sci. U. S. A. 2010;107:9671–6.

107. Ishimura A, Minehata K, Terashima M, Kondoh G, Hara T, Suzuki T. Jmjd5, an H3K36me2 histone demethylase, modulates embryonic cell proliferation through the regulation of Cdkn1a expression. Development. 2012;139:749–59.

108. Hu X, Lu X, Liu R, Ai N, Cao Z, Li Y, et al. Histone cross-talk connects protein phosphatase 1α (PP1α) and histone deacetylase (HDAC) pathways to regulate the functional transition of bromodomain-containing 4 (BRD4) for inducible gene expression. J. Biol. Chem. 2014;289:23154–67.

109. Tzur YB, Egydio de Carvalho C, Nadarajan S, Van Bostelen I, Gu Y, Chu DS, et al. LAB-1 targets PP1 and restricts Aurora B kinase upon entrance into meiosis to promote sister chromatid cohesion. PLoS Biol. 2012;10:e1001378.

110. Sonnhammer ELL, Östlund G. InParanoid 8: orthology analysis between 273 proteomes, mostly eukaryotic. Nucleic Acids Res. 2015;43:D234–9.
